# Comprehensive analyses of the annexin gene family in wheat

**DOI:** 10.1186/s12864-016-2750-y

**Published:** 2016-05-28

**Authors:** Lei Xu, Yimiao Tang, Shiqing Gao, Shichao Su, Lin Hong, Weiwei Wang, Zhaofeng Fang, Xueyin Li, Jinxiu Ma, Wei Quan, Hui Sun, Xia Li, Yongbo Wang, Xiangzheng Liao, Jiangang Gao, Fengting Zhang, Lei Li, Changping Zhao

**Affiliations:** Beijing Engineering Research Center for Hybrid Wheat, The Municipal Key Laboratory of the Molecular Genetics of Hybrid Wheat, Beijing Academy of Agriculture and Forestry Sciences, Beijing, 100097 China; State Key Laboratory of Protein and Plant Gene Research, Peking-Tsinghua Center for Life Sciences, School of Advanced Agricultural Sciences and School of Life Sciences, Peking University, Beijing, 100871 China; College of Life Science, Capital Normal University, Beijing, 100048 China; College of Life Science, Hebei Normal University of Science and Technology, Qinhuangdao, 066600 China

**Keywords:** Hybrid wheat, Annexin, Phylogenetic analysis, Calcium signaling, Cold induction, Thermosensitive genic male sterile (TGMS)

## Abstract

**Background:**

Annexins are an evolutionarily conserved multigene family of calcium-dependent phospholipid binding proteins that play important roles in stress resistance and plant development. They have been relatively well characterized in model plants Arabidopsis (*Arabidopsis thaliana*) and rice (*Oryza sativa*), but nothing has been reported in hexaploid bread wheat (*Triticum aestivum*) and barely (*Hordeum vulgare*), which are the two most economically important plants.

**Results:**

Based on available genomic and transcriptomic data, 25 and 11 putative annexin genes were found through *in silico* analysis in wheat and barley, respectively. Additionally, eight and 11 annexin genes were identified from the draft genome sequences of *Triticum urartu* and *Aegilops tauschii*, progenitor for the A and D genome of wheat, respectively. By phylogenetic analysis, annexins in these four species together with other monocots and eudicots were classified into six different orthologous groups. Pi values of each of *Ann1–12* genes among *T. aestivum*, *T. urartu*, *A. tauschii* and *H. vulgare* species was very low, with the exception of *Ann2* and *Ann5* genes. *Ann2* gene has been under positive selection, but *Ann6* and *Ann7* have been under purifying selection among the four species in their evolutionary histories. The nucleotide diversities of *Ann1–12* genes in the four species were 0.52065, 0.59239, 0.60691 and 0.53421, respectively. No selective pressure was operated on annexin genes in the same species. Gene expression patterns obtained by real-time PCR and re-analyzing the public microarray data revealed differential temporal and spatial regulation of annexin genes in wheat under different abiotic stress conditions such as salinity, drought, cold and abscisic acid. Among those genes, *TaAnn10* is specifically expressed in the anther but fails to be induced by low temperature in thermosensitive genic male sterile lines, suggesting that specific down-regulation of *TaAnn10* is associated with conditional male sterility in wheat.

**Conclusions:**

This study analyzed the size and composition of the annexin gene family in wheat and barley, and investigated differential tissue-specific and stress responsive expression profiles of the gene family in wheat. These results provided significant information for understanding the diverse roles of plant annexins and opened a new avenue for functional studies of cold induced male sterility in wheat.

**Electronic supplementary material:**

The online version of this article (doi:10.1186/s12864-016-2750-y) contains supplementary material, which is available to authorized users.

## Background

Annexins are an evolutionarily conserved multigene family with a broad taxonomic distribution ranging from prokaryotes, protists, fungi, plants, to vertebrates [1]. Annexins are multifunctional proteins that contain the characteristic annexin repeat. The C-terminal core of a typical mammalian annexin consists of four annexin repeats, and each repeat is approximately 70-amino-acid-long. The annexin repeat comprises five short α-helices and usually contains a characteristic “type II” motif (with the sequence GxGT-[38 residues]-D/E) for binding calcium ions [2]. Different annexins are thus distinguished by their highly variable N-terminal regions which are important bases for determining functional differences of family members [2, 3].

Functionally, annexins are capable of binding negatively charged phospholipids in a calcium dependent manner and also possess protein domains implicated in diverse cellular functions such as exocytosis, actin binding, peroxidase activity, callose synthase regulation and ion transport [1, 2, 4]. Furthermore, their association or insertion into membranes may be governed by a range of cellular states [2]. Unique roles of plant annexins include pH-mediated cellular response to environmental stimuli, fiber elongation, abscisic acid (ABA) signal transduction, and osmotic stress tolerance [2]. Therefore, annexins appear capable of dynamically linking calcium, redox and lipid signaling to coordinate development in response to the changing environment [2, 5].

In plants, annexins have been found in eudicot species including Arabidopsis [6], pea [7], cotton [8, 9], potato [10] and tobacco [11, 12], as well as monocot species including rice [13], maize [14], and wheat [15, 16]. Although evolved from a common ancestor, plant annexins are structurally different from their animal counterparts. Plant annexins have only one or two conserved annexin repeats and have a shorter N-terminal region [17]. Structurally, plant annexins have larger surface area due to extra grooves and clefts in contrasting to mammalian annexins, suggesting that plant annexins may have wider range of interaction partners and hence a broad range of cellular roles. In addition to calcium channel and actin binding activities, plant annexins have some motifs or residues suggestive of peroxidase and ATPase/GTPase activit [2, 4]. Moreover, plant annexins also possess a variety of post-translational modification sites, at least some of which may act as important regulators for their function in calcium dependent signaling [8].

As shown by many transcriptional analyses, many plant annexin genes are transcriptionally activated in response to various abiotic stresses and plant hormones. An alfalfa annexin gene (*MsAnn2*), which can be activated by osmotic stress, drought and ABA, was first reported [18]. Subsequently, transcriptional induction of annexin genes by stress conditions such as drought, salinity, cold, heat, heavy metal and oxidative stresses or by phytohormones such as ABA, jasmonic acid, ethylene, salicylic acid and auxin has been widely reported in many plants, including Arabidopsis [19], leaf mustard [20], tobacco [11], rice [13], wheat [15, 16] and maize [14].

Known plant annexins are often differentially expressed at different developmental stages. Specific cases were observed for annexin gene families in dicots such as Arabidopsis [6, 19], leaf mustard [20, 21], tomato [22] and bell pepper [17], in monocots such as rice [13] and maize [14, 23]. Of particular interest is that plant annexins are usually prominent at apical cells undergoing polar elongation, such as root hairs, pollen tubes and fern rhizoids [23–26].

Several plant annexin genes have been genetically studied. For example, two annexin genes in *Arabidopsis*, termed *AtANN1* and *AtANN4*, regulate stress responses. While the *AtAnn1* and *AtAnn4* single mutants showed tolerance to drought and salinity, which was further enhanced in the double mutant, constitutively expressing *AnnAt4* sensitized plants to stress treatments [27–29]. *AtAnn5* was specifically expressed in mature pollen, and silencing *AtAnn5* resulted in abnormal pollen grain and severe male sterility [30]. Ectopic expression of *BjAnn1*, a *B. juncea* annexin gene, in tobacco and cotton enhanced tolerance to various abiotic stresses and fungal pathogen attack [21, 31]. Down-regulating *GhAnn2* in cotton inhibited cotton fiber elongation, presumably through decreasing calcium influx at apical cells [32].

Wheat is a cereal crop of immense agricultural importance. In contrast to rice, corn, cotton and other crops in which heterosis has been extensively exploited in breeding programs, utilization of heterosis in wheat is still situated in an initial stage [33]. Recently, efforts to exploit wheat heterosis have made promising progress by combining recovery lines with thermosensitive genic male sterile (TGMS) lines. This two-line system is advantageous over the cytoplasmic male sterility system for its broad donor of restoring ability, easy maintenance and multiplication [33]. Transcriptome profiling studies revealed that genes associated with calcium signaling and cytoskeleton dynamics were compromised upon cold stress in the anther of the TGMS lines [34, 35].

Based on previous knowledge, we are interested in determining whether annexins are involved in regulating male fertility in wheat. Thus far, comprehensive information about the annexin gene family in wheat and related species still remains unclear. In this study, we annotated and phylogenetically analyzed annexin genes in wheat and barley, which have probably diverged from the *Triticum* lineage about 11 million years ago (MYA) [36], and in *T. urartu* and *A. tauschii* which are progenitors for the A and D genome of wheat, respectively. Based on such information, we further performed detailed transcriptomic analyses of annexin genes in wheat. Together, the results provided significant information for understanding the diverse roles of plant annexins and opened a new avenue for functional studies of cold induced male sterility in wheat.

## Results

### Identification of annexins from *Triticum aestivum, Triticum urartu*, *Aegilops tauschii* and *Hordeum vulgare*

We searched annexin genes systematically from available genomic and transcriptomic data of *T. aestivum*, *T. urartu*, *A. tauschii* and *H. vulgare*. In the hexaploid *T. aestivum*, 24 putative full-length annexin genes were identified based on incomplete draft genome sequences, expressed sequence tags (ESTs), and whole genome survey sequences (WSS) [37]. An additional annexin-coding sequence, *TaAnn10-A* (GenBank accession: KT198661), was assembled independently from Hiseq2000 (Illumina Solexa) cDNA sequences (Additional file 1). Putative open reading frames and protein sequences were deduced from the 25 annexin-coding sequences, searched against the Pfam database [38], and found to contain at least two annexin domains. Finally, these 25 annexin genes were assigned to the A, B, and D subgenome. It was observed that there are 12 members of an annexin gene family in each haploid genome in wheat. Eight annexin genes in *T. urartu* and elven in *A. tauschii* were identified from their complete genome sequences (Additional file 1). All assembled coding sequences were verified as full-length by comparison to the wheat sequences. Similarly, eleven full-length cDNA sequences of annexin genes were identified in *H. vulgare* (Additional file 1), of which two were obtained from GenBank while the other eight tentatively predicted from the second generation barley genomic sequences.

At deduced amino acid level, the average size of encoded polypeptides of the identified annexin genes from wheat, *T. urartu, A. tauschii* and barely was approximately 36 kD, similar to that of annotated annexins in *Arabidopsis thaliana*, *Oryza sativa* and *Brachypodium distachyon*. Their predicted pI points of the identified annexin proteins were very divergent, ranging from 5.28 to 9.4, 6.15 to 9.82, 6.36 to 9.07, and 6.47 to 9.82 in wheat, *T. urartu, A. tauschii* and barley, respectively. Aliphatic index showed a range of 74.96 to 96.83. The grand average of hydropathicity showed similar pattern, suggesting that above identified putative annexins are hydrophobic proteins (Additional file 2). Similar to other plant annexins [13, 22], the deduced amino acid sequences of wheat annexins contain several conserved residues/motifs in addition to the annexin repeat, such as endonexin fold, a cysteine-rich and a zinc finger type (C2H2) domain signature sequence, the His40 residue for peroxidase activity, S3 cluster putatively involved in redox reactions, salt bridges involved in the channel function of animal annexins, and IRI actin-binding motif (Additional file 3).

### Structure and phylogeny of annexin gene family

**Fig. 1:**
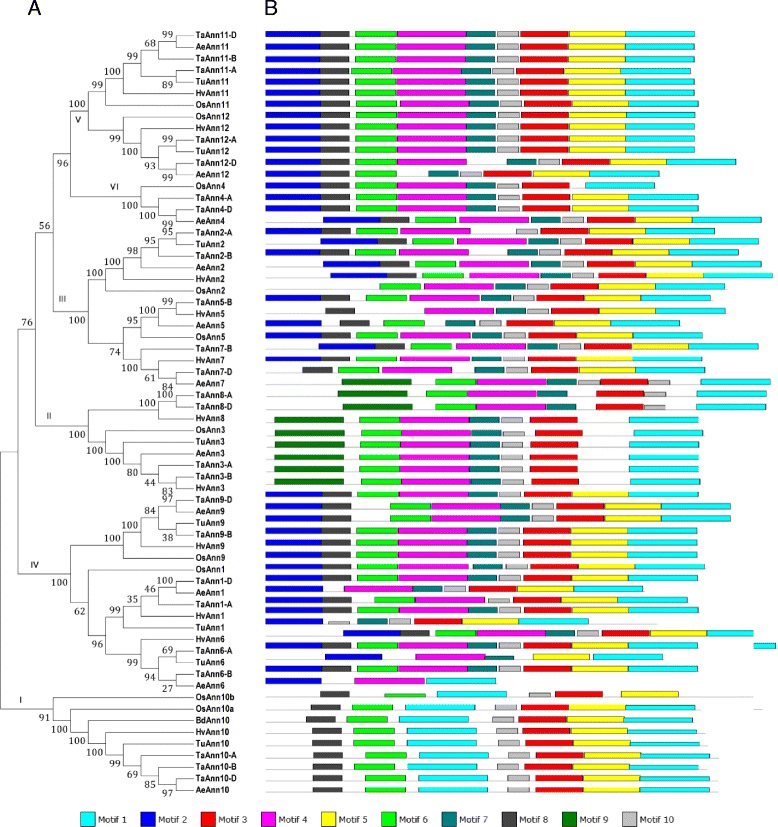
Phylogenetic analysis and predicted structure of annexin proteins in *T. aestivum*, *T. urartu*, *A. tauschii*, *H. vulgare* and *O. sativa* **a** Phylogenetic analysis of annexin protein sequences from *T. aestivum*, *T. urartu*, *A. tauschii*, *H. vulgare* and *O. sativa.* The tree was classified into six groups represented by roman letters (in black boxes). **b** Conserved motifs of anexin proteins from *T. aestivum*, *T. urartu*, *A. tauschii*, *H. vulgare* and *O. sativa* obtained by the MEME 4.6.1 software. The 1, 2, 3 and 4 motifs were found to be functionally associated with calcium-dependent phospholipid binding/calcium ion binding

The primary sequences of annexins in *T. aestivum*, *T. urartu*, *A. tauschii* and *H. vulgare* fairly diverged from each other. In these four species, identities of the annexins were 19–83 %, 19–78 %, 17–78 % and 19–83 %, respectively. To facilitate comparison of the annexin families in different species, we analyzed conserved motifs of 65 annexins from *T. aestivum*, *T. urartu*, *A. tauschii*, *H. vulgare* and *O. sativa*. The results showed that these 65 annexins were classified into 6 groups (Fig. 1a). Meanwhile, a total of 10 motifs containing 6 to 50 residues were identified and each of these motifs was annotated by InterProScan and SMART databases (Fig. 1b and Additional file 4). The members of each group shared similar motif organizations. Four motifs named 1, 2, 3, 4 were found to be associated with calcium-dependent phospholipid binding/calcium ion binding function. The diversity of motif patterns based on combinations of motifs 1, 2, 3, 4 was in agreement with the phylogenetic analysis (Additional file 5), besides, these motif patterns were conserved and specific to each group (Fig. 1 and Additional file 4).

**Fig. 2:**
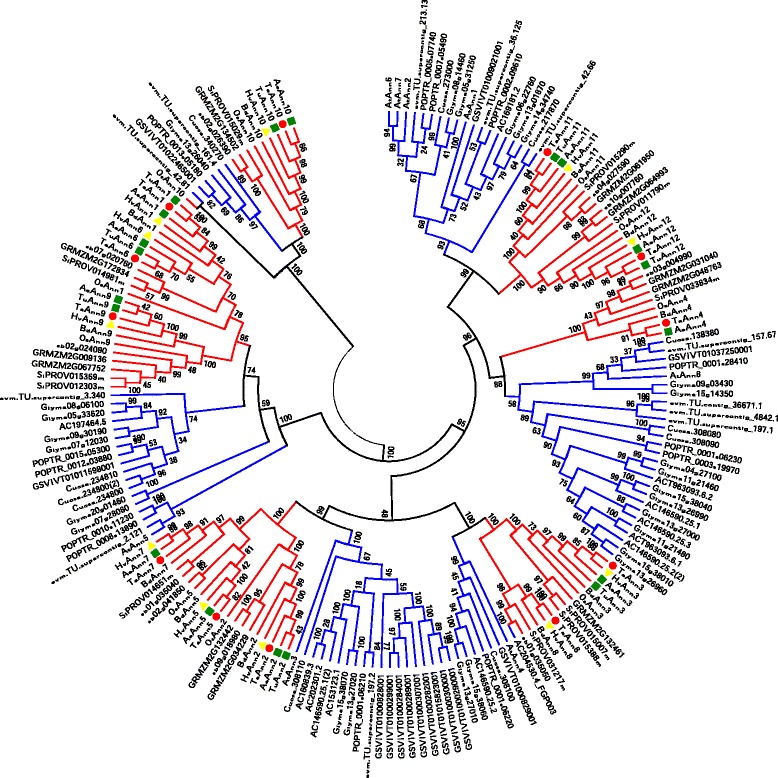
Phylogenetic analysis of annexin protein sequences from 16 plant species. Unrooted phylogenetic tree using 169 full-length amino acid sequences from nine monocot species and seven dicots species. Numbers on the tree represent bootstrap values. The roman letters represent the six groups. The different branch color represents monocots (pink) and dicots (blue), respectively. Members of annexin proteins from wheat were denoted in red and barley in green

To analyze the evolutionary relationship of annexins, an unrooted tree was constructed using 169 full-length annexin protein sequences from nine monocot species and seven eudicots. The tree showed that all annexin homologs were clustered into six groups (I–VI) with high statistical support (Fig. 2). In each clade, it can be separated into a monocots subgroup and a eudicots subgroup, except for *OsAnn10b* (Os09g20330.1) [13]. As the annexin multigene family appeared to expand possibly by duplication events [39], phylogenetic analyses suggest that the six groups have their own ancestors after their divergence from angiosperm flowering plants and then each subgroup have parallel evolution in similar way diverging into monocots and dicots. Some annexins from the same species were clustered into one subgroup, such as most of grape vine annexins were present in group III. We also observed the orthologous annexins of wheat in other monocots (Additional file 6 and Additional file 7).

**Fig. 3:**
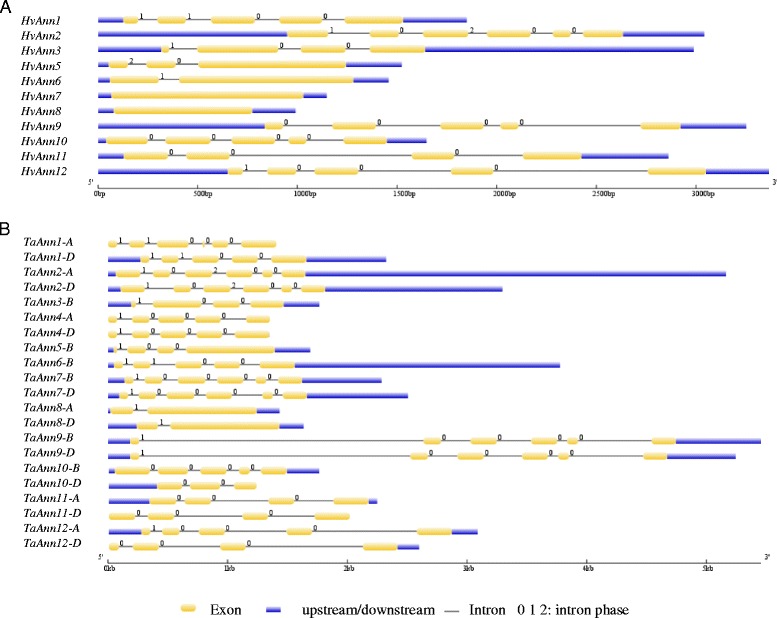
Gene structure of annexin genes in barley and wheat. **a** barley; **b** wheat. Gene structures were generated from GSDS (http://gsds.cbi.pku.edu.cn/). Exons, UTRs, introns and intron phases are shown

Exon-intron organization structure analysis of annexin genes in wheat and barley revealed that the number of introns per gene varied from 1 to 5. The intron positions of orthologous annexin genes (except for *TaAnn5*, *HvAnn5*, *TaAnn6*, *HvAnn6*) in wheat and barley and their phases with symmetric exons are well conserved indicating that all these annexin genes might have a common ancestor (Fig. 3).

### Comparative analysis of annexins between monocots and eudicots

Six distinct clusters of annexins were revealed by phylogenetic analysis of annexin proteins from three monocots (wheat, barley and rice) and two eudicots (*A. thaliana* and *G. max*) (Additional file 8). Within in each subgroup, two distinct clusters corresponding to monocots and eudicots were formed except for *OsAnn10a*. Within every subgroup, monocots (rice, barley and wheat) showed less divergence as indicated by a shorter branch length, which marks sequence changes in species relative to its common ancestor, compared to the examined dicots (Arabidopsis and soybean) (Additional file 8). Regarding protein structure, we also found the four domains of annexins in monocots were relatively more conserved than that in eudicots by alignment analysis of amino acid sequence (Additional file 9). Interestingly, we observed that the IRI motif for binding actin in the third domain was only conserved in the IV subfamily in monocots. In addition, several highly conserved charged residues were only found in annexin domains of monocots, such as domain-2 (DL; aa 24 and 25) (Additional file 9). These data showed that the structure of annexin proteins in monocots was more stable than that in eudicots, suggesting that annexins probably played important roles in the evolution history of monocots.

Although the sizes of predicted annexin proteins were highly similar in eudicots and monocots tested, comparison of the genomic sequences revealed a novel pattern of intron evolution. Interestingly, the total sizes of exomes in eudicots and monocots were not much different, but large differences in intron sizes of some annexin genes were observed between eudicots (50 bp-800 bp) and monocots (50 bp–3,500 bp) (Additional file 10). Changes in the sizes of introns without significantly affecting the protein sequence or structure suggest that at least for annexin genes, the evolution of introns is different from that of extrons.

### Phylogenetic analysis of annexin genes in *T. aestivum*, *T. urartu*, *A. tauschii* and *H. vulgare*

To further analyze the evolutionary relationships of annexin genes in *T. aestivum*, *T. urartu*, *A. tauschii* and *H. vulgare* species, the genetic variation and selective pressure of each of *Ann1*–*12* genes among the four species were measured by the software of DnaSP 5.0 (Tables 1, 2 and 3). The results showed that the nucleotide diversities (Pi value) of most annexin genes among different species were very low, except for *Ann2* and *Ann5* genes with Pi values of 0.20876 and 0.35632, respectively (Table 1). To detect whether annexin genes have been under selective pressures among the four species, we first made the neutral model test for every annexin gene by Tajima's D analysis. Only *Ann2*, *Ann6*, *Ann7* genes exhibited significant departures from neutral expectations, suggesting that these genes have been under selective pressures in their evolutionary histories. The NonSyn/Syn (Nonsynonymous substitutions/synonymous substitutions) ratios of *Ann2*, *Ann6* and *Ann7* genes were 1.97715, 0.89736 and 0.37358, respectively, indicating that *Ann2* gene has been under positive selection, but *Ann6* and *Ann7* have been under purifying selection (Table 2).

**Table 1:** The evolution analysis of *Ann1*–*12* genes among and within *T. aestivum*, *T. urartu*, *A. tauschii* and *H. vulgare* species by DnaSP 5.0 software

Gene	Nucleotide diversity (Pi)
*Ann1*	0.05427
*Ann2*	0.20876
*Ann3*	0.04861
*Ann4*	0.04598
*Ann5*	0.35632
*Ann6*	0.12454
*Ann7*	0.05773
*Ann8*	0.03352
*Ann9*	0.04115
*Ann10*	0.04904
*Ann11*	0.13112
*Ann12*	0.17971

A. The nucleotide diversity of each annexin gene among the four species

The significance threshold is *P* < 0.05

**Table 2:** The evolution analysis of *Ann1*–*12* genes among and within *T. aestivum*, *T. urartu*, *A. tauschii* and *H. vulgare* species by DnaSP 5.0 software

Gene	Tajima's D	*P* value of Tajima's D	NonSyn/Syn
*Ann2*	−0.93395	*P* < 0.001	1.97715
*Ann6*	−1.01556	*P* < 0.001	0.89736
*Ann7*	−1.22115	*P* < 0.001	0.37358

B. The annexin genes that have been under selective pressures among different species

The significance threshold is *P* < 0.05

**Table 3:** The evolution analysis of *Ann1*–*12* genes among and within *T. aestivum*, *T. urartu*, *A. tauschii* and *H. vulgare* species by DnaSP 5.0 software

Species	Nucleotide diversity (Pi)	Tajima's D	*P* value of Tajima's D
*T.aestivum*	0.52065	−0.35165	*P* > 0.10
*T.urartu*	0.59239	−1.24132	*P* > 0.10
*A.tauschii*	0.60691	−1.3584	*P* > 0.10
*H. vulgare*	0.53421	−1.04232	*P* > 0.10

C. The nucleotide diversity and neutrality test of *Ann1*–*12* genes in *T. aestivum*, *T. urartu*, *A. tauschii* and *H. vulgare* species, respectively

The significance threshold is *P* < 0.05

By analyzing the genetic variations and selective pressures of *Ann1*–*12* genes within each of the four species (Table 3), we found the nucleotide diversities of *Ann1*–*12* genes in *T. aestivum*, *T. urartu*, *A. tauschii* and *H. vulgare* species were 0.52065, 0.59239, 0.60691, 0.53421, respectively, which were obviously higher than that of each *Ann* gene among different species as shown in Table 1. These results indicated that the conservation level of each *Ann* gene among different species was higher than that of *Ann1*–*12* genes in the same species. In addition, by the Tajima's D analysis, we found *Ann1*–*12* genes in each species did not exhibit significant deviation from neutral expectations, indicating that no selective pressure was operated on annexin genes in the same species.

### Expression analysis of annexin genes in response to abiotic stresses

**Fig. 4:**
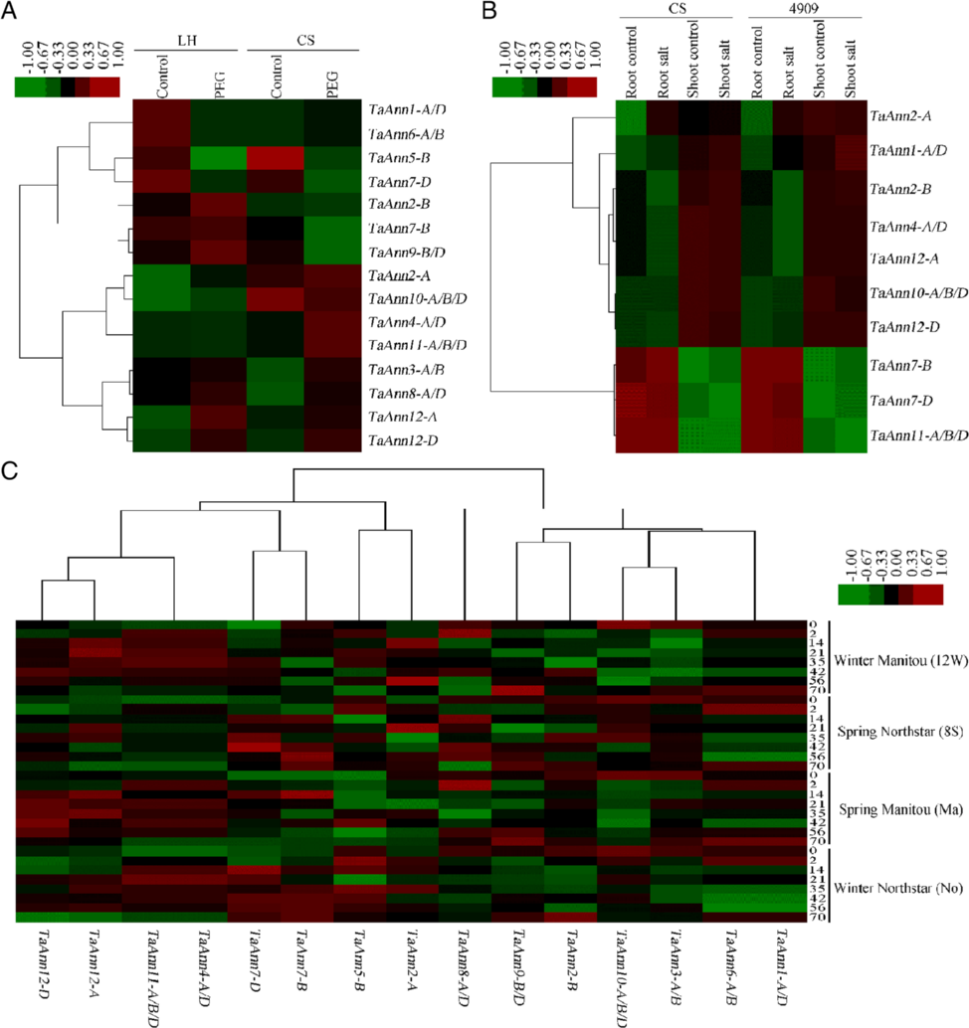
Expression patterns of 15 *TaAnn* genes under drought, salt and cold conditions. Heat maps were made based on the published microarray data to represent expression patterns. **a** The heat map for expression patterns under drought condition. Expression data were obtained from TA43 (GSE30872) experiment. The plant materials used are roots of Luohan (LH) and Chinese Spring (CS) seedlings. **b** The heat map for expression patterns under salt condition. Expression data were obtained from E-MEXP-971 experiment. Roots and shoots of a highly salt-tolerant wheat line 4909 and a salt-sensitive line CS were used. **c** The heat map for expression patterns under cold treatment. Expression data were got from TA42 (GSE23889) experiment

Accumulating evidences from various plant species, including Arabidopsis, tomato and rice, have shown the up- and down-regulation of annexin genes in response to abiotic stress and ABA [13, 22, 40]. To examine the expression patterns of 12 wheat annexin genes (15 transcripts) under various abiotic stress conditions, we took advantage of the available data on transcriptional profiling (http://www.plexdb.org/). The analysis of microarray data indicated that some of the annexin genes were regulated by various abiotic stress conditions (Fig. 4). The expression of *TaAnn12-A* and *TaAnn12-D* were induced, reversely, *TaAnn5-B* and *TaAnn7-D* were down-regulated by drought both in the drought tolerant 'Luohan No.2' (LH) and drought susceptible 'Chinese Spring' (CS). Meanwhile, *TaAnn1-A/D* and *TaAnn6-A/B* were down-regulated in 'Luohan No.2' (LH) but up-regulated in 'Chinese Spring' (CS) under PEG treatment (Fig. 4a). *TaAnn4-A/D* and *TaAnn12-A* were reduced by salt in the root and shoot samples of salt tolerant wheat germplasm lines W4909 and salt susceptible 'Chinese Spring' (CS) (Fig. 4b). *TaAnn12-A* and *TaAnn12-D* were strongly induced, reversely, *TaAnn1-A/D* and *TaAnn2-B* were down-regulated by cold stress in Winter Manitou (12 W), Spring Northstar (8S), Spring Manitou (Ma), and Winter Northstar (No) (Fig. 4c). These microarray data indicate that *TaAnn1*, *TaAnn2, TaAnn4*, *TaAnn5*, *TaAnn6*, *TaAnn7* and *TaAnn12* probably play important roles in abiotic stress response.

**Fig. 5:**
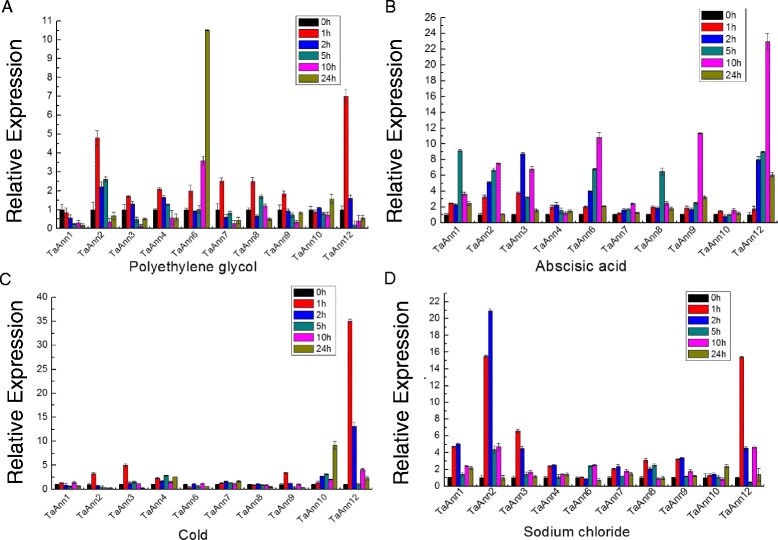
Expression of wheat annexin genes in response to abiotic stress and ABA by real-time PCR. Wheat Jinghua 9 seedlings grown for 14 days on MS medium were treated with PEG-6000 (25 % w/v) (**a**) 200 ummol · L^−1^ ABA (**b**) 4 °C (**c**) and 250 mM NaCl (**d**) for 0, 1, 2, 5, 10 and 24 h, respectively. Total RNA was extracted from Jinghua 9 seedlings and used for real-time PCR. The expression level of wheat actin was used as the internal control to standardize the RNA samples for each reaction, and the expression in the treated 0 h samples was set as 1. Each data point is the average of three biological repeats, and error bars represent the standard error

To confirm the results of microarray data, and identify the wheat annexin genes that respond to abiotic stresses and ABA, we checked the transcription levels of *TaAnn1*–*12* genes (except for *TaAnn5* and *TaAnn11*) in seedling shoots challenged with salt, drought, cold and ABA. Some wheat annexin genes such as *TaAnn1*, *TaAnn2*, *TaAnn6* and *TaAnn12* were consistent with the gene chip data, (Figs. 4 and 5). Most wheat annexin genes were induced by single or several stresses,but the expression levels of *TaAnn4* and *TaAnn7* were not obviously changed under different stress conditions. All wheat annexin genes, except for *TaAnn4*, *TaAnn7* and *TaAnn10*, were induced by ABA (Fig. 5b). Interestingly, *TaAnn9*, which showed no obvious change under salt, drought and cold, was induced by ABA, implying its important role in ABA response (Fig. 5b). *TaAnn1* was down-regulated to the control level after the drought treatment for 5 h, but was obviously up-regulated under salt and ABA, which is consist with the microarray data in Fig. 4 (Fig. 5a). The expression level of *TaAnn8* was nearly unchanged under cold, but was increased under drought, salt and ABA conditions. *TaAnn2* and *TaAnn12* were significantly induced by salt, drought, cold and ABA, indicating that the two genes play key roles in responses to abiotic stresses. Interestingly, *TaAnn12*, which is an exciting candidate drought, cold, salt tolerance gene in crop breeding, has no orthologs in *A. thaliana*.

We also analyzed the putative cis-elements within the 2 Kb region of genomic sequences upstream of the 5′-UTR of wheat annexin genes except for *TaAnn1-D* (1,768 bp) and *TaAnn6-B* (1,302 bp) to search for stress-response related cis-elements in PLACE database (http://www.dna.affrc.go.jp/PLACE/signalscan.html). The results showed that 15 annexin genes contain the ABRE (ACGTG), DRE/CRT (G/ACCGCC) and LTRE (CCGAC) putative cis-elements motifs (Additional file 11). In addition,the promoters of 14 annexin genes, 11 annexin and 12 annexin genes only contain ABRE, DRE/CRT or LTRE elements, respectively. ABRE and DRE/CRT motifs are responsible for salinity, dehydration, heat and cold stresses, and LTRE motif is responsible for cold stress [40].

### Tissue-specific and developmental expression of wheat annexin genes

**Fig. 6:**
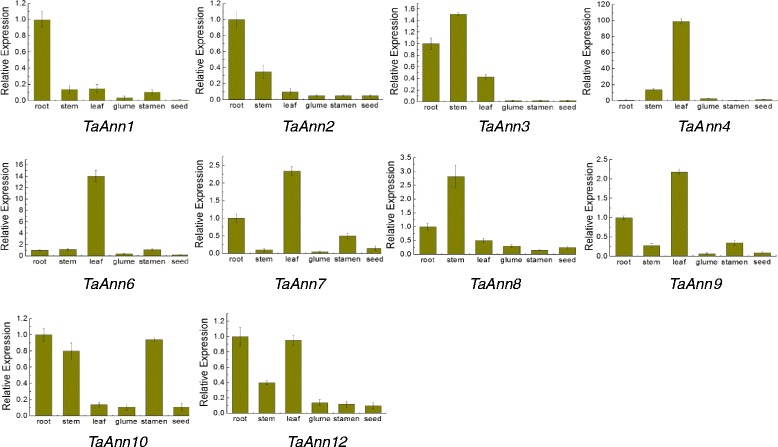
Real-time PCR analysis of wheat annexin genes in pivotal organs. The expression level of wheat actin was used as the internal control to standardize the RNA samples for each reaction, and the expression in the root was set as 1. The data are from three biological replicates, and error bars represent the standard error

**Fig. 7:**
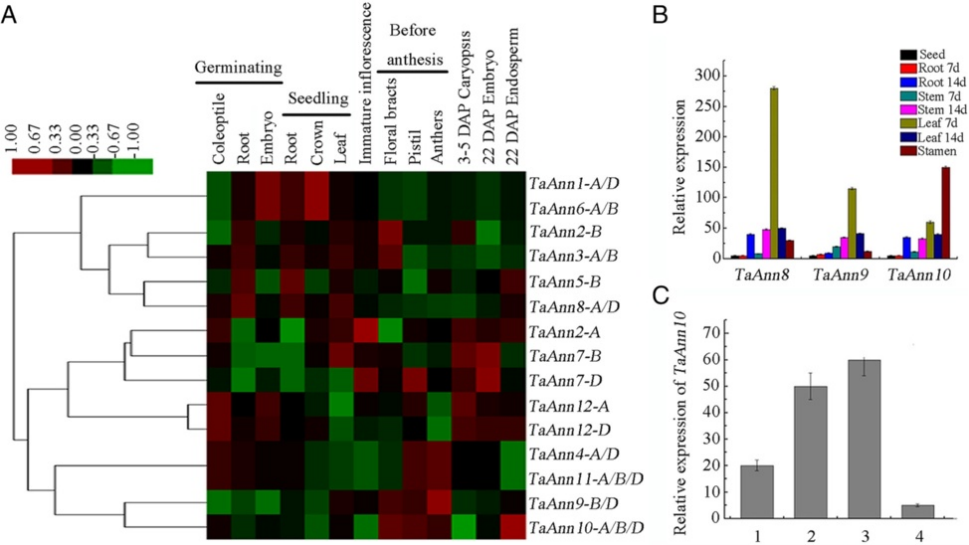
Tissue-specific expression patterns of *TaAnn* genes. **a** Tissue-specific expression patterns of 15 *TaAnn* genes detected in the microarray data (GSE12508). The heat map was generated by hierarchical clustering based on Pearson’s correlation, and log2 ratios of expression were used to make the map. **b** Expression levels of *TaAnn8*, *TaAnn9* and *TaAnn10* genes in wheat seedings and stamen before flowering. Total RNA extracted from the seed, 7-day and 14-day-old young root, stem, leaf tissues and stamen before flowering of wheat Jinghua 9 was used for real-time PCR. The expression in the seed sample was set as 1. **c** The expression pattern of *TaAnn10* gene in the taiyuan806 recovery line. Total RNA extracted from the spike at stamen and pistil initiation stage, anther at anther separation stage, anther at meiosis stage, and spike not including anther at meiosis stage of wheat taiyuan806 was used for real-time PCR. 1 is spike at stamen and pistil initiation stage; 2 is anther at anther seperation stage; 3 is anther at meiosis stage; 4 is spike not including anther at meiosis stage. The expression at the spike not including anther at meiosis stage sample was set as 1. The expression level of wheat actin was used as the internal control to standardize the RNA samples for each reaction. The data are from three biological replicates, and error bars represent the standard error

To profile the developmental expression pattern of annexin genes in wheat, we analyzed the expression levels of ten *TaAnn* genes in six organs by real-time PCR (Fig. 6). *TaAnn1* and *TaAnn2* were mainly expressed in root, but *TaAnn4*, *TaAnn6*, *TaAnn7* and *TaAnn9* were mainly expressed in leaf. The expression levels of *TaAnn3* and *TaAnn8* were highest in stem followed by root, and were very low in glume, stamen and seed tissues. The expression levels of *TaAnn10* in root, stem and stamen were similar, and significantly higher than that in leaf, glume and seed. The higher expression of *TaAnn12* was observed in root and leaf. To complement the real-time PCR results, we analyzed the microarray data covering genome-wide gene expression of wheat development [41]. The result was an expression atlas of 12 wheat annexin genes (15 transcripts) covering major developmental stages (13 individual tissues) during the life cycle of wheat (Fig. 7a). The results also showed that *TaAnn1* and *TaAnn6* were mainly expressed in germinating embryo and seedling crown. The highest expression levels of *TaAnn2* and *TaAnn3* were observed in floral bracts before anthesis. For *TaAnn5* and *TaAnn8*, expression levels were higher in the root of germinating seed and the root as well as the leaf of seedling. The highest expression level of *TaAnn2* was detected in immature inflorescence, by contrast, that of *TaAnn7* was observed in the immature embryo. *TaAnn12* was mainly expressed in the coleoptile of germinating seed and the immature caryopsis, however, the highest expression levels of *TaAnn4*, *TaAnn9* and *TaAnn11* were detected in anthers before anthesis. *TaAnn10* was highly expressed in floral bracts, pistil, anthers and immature endosperm which were consistent with the gene chip data (Figs. 6 and 7a). To confirm the results of microarray data, we checked the transcription levels of *TaAnn8*, *TaAnn9*, and *TaAnn10* in 7-day-old root, 7-day-old stem, 7-day-old leaf, 14-day-old root, 14-day-old stem, 14-day-old leaf, stamen before flowering and 12 DAP seed by real-time PCR. We found the highest expression levels of *TaAnn8* and *TaAnn9* in 7-day-old young leaf followed by 14-day-old leaf and14-day-old stem, and the expression level of *TaAnn10* was highest in anther followed by 7-day-old young leaf (Fig. 7b). These results were in accordance with the microarray data (Fig. 7a). To further confirm *TaAnn10* expression pattern in flower phase, we also analyzed the expression level of *TaAnn10* in the spike at stamen and pistil initiation stage, anther at anther separation stage, anther at meiosis stage, and spike not including anther at meiosis stage. The results showed *TaAnn10* was highly expressed in anther, and its expression levels in the pistil and floral bracts were relatively low (Fig. 7c). Also the highest expression level of *TaAnn10* in anther at meiosis stage was 12-fold and 5-fold higher than that in spike not including anther at meiosis stage and spike at stamen and pistil initiation stage, respectively (Fig. 7c). These results suggested that *TaAnn10* was possible to be related to plant anther development.

### *TaAnn10* is involved in cold induced male sterility

**Fig. 8:**
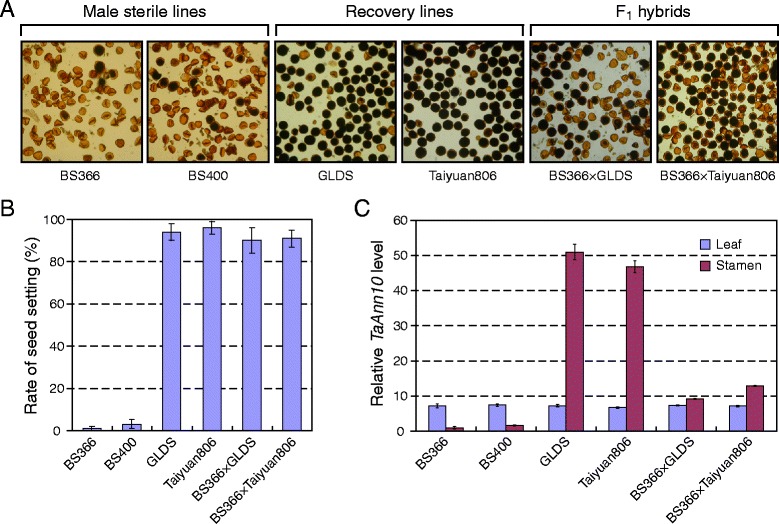
Down-regulation of *TaAnn10* is associated with the cold-induced male fertility. **a** Under cold stress (Cold treatment, comprising 10 °C with a 12 h photoperiod), pollen grains from two male sterile lines BS366 and BS400, two recovery lines GLDS and taiyuan806, and BS366 × GLDS and BS366 × Taiyuan806 hybrids lines anthers were devoid of starch (stained with I2-KI). **b** Rate of seed setting from two male sterile lines BS366 and BS400, two recovery lines GLDS and taiyuan806, and BS366 × GLDS and BS366 × Taiyuan806 hybrids lines. Data presented as means ± SD from three independent experiments, each with fifty plants per line per experiment. **c** The expression level of *TaAnn10* gene in leaf (blue) and anther (red) at meiosis stage under cold treatment (comprising 10 °C with a 12 h photoperiod, sterile conditions), in two male sterile lines BS366 and BS400, two recovery lines GLDS and taiyuan806, and two hybrid lines BS366 × GLDS and BS366 × Taiyuan806. The expression level of wheat actin was used as the internal control to standardize the RNA samples for each reaction. The real-time PCR data are from three biological replicates, and error bars represent the standard error

Development-dependent and temperature sensitive expression patterns of *TaAnn10* in the anther prompted us to investigate whether it is involved in male reproductive development. To this end, we employed the two-line hybrid wheat system, examined pollen vitality and profiled expression pattern of *TaAnn10* in six wheat lines. BS366 and BS400 are two thermosensitive genic male sterile lines extensively used for the production of two-line hybrid wheat [33]. Pollens from both lines displayed vastly reduced vitality when the plants were grown under non-permissive temperature (cold treatment at 10 °C during meiosis) (Fig. 8a). GLDS and Taiyuan806 are two independent recovery lines that have the ability to generate fully fertile F1 hybrid lines when crossed with the sterile lines as the pollen donor. Indeed, pollens from GLSD and Taiyuan806 exhibited superior vitality (Fig. 8a). In BS366 × GLDS and BS366 × Taiyuan806 hybrid lines, pollen vitality appeared comparable to that of the recovery lines. Consistent with pollen vitality, the two sterile lines have vastly reduced rate of seed setting, in contrast to the other four lines (Fig. 8b).

By real-time PCR, we profiled the expression of *TaAnn10* in the leaf and stamen of the six wheat lines (Fig. 8c). The expression of *TaAnn10* in the leaf across the six lines remained constant. Consistent with tissue specificity reported in Fig. 7b, in the two recovery lines, the expression level of *TaAnn10* in the stamen greatly exceeded that in the leaf. By contrast, in the two conditional sterile lines BS366 and BS400, *TaAnn10* levels in the stamen were markedly lower than that in the leaf. Importantly, the expression level of *TaAnn10* in the stamen was partially restored in the two F1 hybrids. Therefore, the relative expression levels of *TaAnn10* in the stamen strongly correlate with male fertility in the six examined lines, suggesting that specific down-regulation of *TaAnn10* is associated with conditional male sterility in wheat.

## Discussion

### The Annexin family genes and their structures

Only two monocot plant annexins families, rice and maize, have been characterized based on the genome sequence data [13, 14]. Now, only three annexin genes in wheat (p39, p22.5 and P35) have been characterized [15, 16]. In this study, six of the 25 wheat annexin genes identified in the EST databases at GenBank were also identified in the WSS. *TaAnn1-D*, *TaAnn2-B*, *TaAnn3-B*, *TaAnn4-A*, *TaAnn4-D*, *TaAnn5-B*, *TaAnn6-B*, *TaAnn7-B*, *TaAnn7-D*, *TaAnn9-B*, *TaAnn10-B*, *TaAnn11-A*, *TaAnn11-D* and *TaAnn12-D* which were not represented in *T. aestivum* EST database at GenBank were identified in the WSS. *Brachypodium distachyon*, the most closely wheat related monocot species with an available completed and annotated genome sequence, has 11 annexin genes. Rice (*Oryza sativa*) has 10 annexin genes. Nearly all annexin genes in *O. sativa* and *B. distachyon* except for *OsAnn1* and *BdAnn7* have orthologs in *T. aestivuma*, *H. vulgare*, *T. urartu* and *A. tauschii*, as judged by OrthoMCL (v1.4) software (Additional file 6 and Additional file 7).

His residue was thought to be essential for peroxidase activity [42], and we found 18 wheat annexins contain conserved His residue at the N-terminal region (Additional file 3). Certain wheat annexins also contain the predicted IRI motif involved in F-actin and GTP-binding regions (GXXXXGKT and DXXG) for phosphodiesterase activity [43]. The deduced proteins also contain residues for the formation of salt bridges that are hypothesized to be involved in the formation of ion channel activity [5] (Additional file 3). Wheat annexins also contain conserved residues that are involved in the potential formation of an unusual sulfur cluster (S3) functioning in oxidative stress response [44]. In addition, the N-terminal regions of *TaAnn2-A* and *TaAnn2-B* both contain a cysteine-rich and a zinc finger type (C2H2) domain signature sequence. The C2H2 zinc finger proteins are involved in transcriptional regulation by binding DNA or RNA [45]. These results suggest that the wheat annexin gene family probably participate in many different biological processes.

All annexins are speculated to have evolved from a common ancestor, and increased gene number may have resulted from gene duplication events, which can be seen from amino acid sequence similarities and their genome locations [46]. Since these distantly related species have gene family members which share branches with sequences, from wheat throughout the tree, it seems that the representation of annexin from wheat is likely complete (Fig. 2). The larger number of annexin genes in Triticeae, supported by identified sequences from wheat, *T. urartu, A. tauschii* and barley, and the representation of genes from *O. sativa* on the major sub-branches in the phylogram indicate that the Triticeae had two gene duplications after the evolutionary separation from rice, and before the separation of the three wheat progenitor species from each other. These duplications in the Triticeae lineage after the separation from rice are the *TaAnn3* and *TaAnn8* duplication, and subsequently the *TaAnn1* and *TaAnn6* duplication (Fig. 2). The localization of these putative gene duplications on the same chromosomes supports this notion, since gene duplications often occur as tandem duplications [40], though further investigation would be required to demonstrate that the duplications were in tandem. *TuAnn8* and *AeAnn8* were not identified in *T. urartu* and *A. tauschii* data sets in GenBank or WSS. This may represent gene loss, loss of gene expression or may be due to incomplete transcriptome or genome sequence from these species in the current databases. The lengths of branches were scaled according to amino acid differences between sequences, thus providing an estimate of evolutionary distance. Grape annexin genes were largely clustered together in the phylogenetic tree, suggesting that gene duplication events occurred independently in the monocotyledonous and dicotyledonous branches of the tree (Fig. 2). The structural and phylogenetic analyses indicate that orthologous annexin genes within a group might have functional redundancy (Additional file 5). However, the gene expression study with respect to different tissues, developmental stages and abiotic stressors suggests that many members of the wheat annexins gene family have non-overlapping functions (Figs. 5 and 6).

### Implication of cis-elements in annexin gene expression

Cis-elements are important molecular switches involved in the transcriptional regulation of genes during abiotic stress responses and may be induced through ABA-dependent and ABA-independent signal transduction pathways [40]. For ABA responsiveness, all annexin genes contain ABRE-motif elements within their promoter sequences, with the exception of *TaAnn6-B* (Additional file 11). However, *TaAnn6* gene was up-regulated under ABA treatment, while the expression levels of *TaAnn4*, *TaAnn7* and *TaAnn10* were not significantly changed (Fig. 5b). The promoters of seven annexin genes (*TaAnn1*, *TaAnn7*–*12*) contain DRE/CRT motif responsible for activation during salinity, dehydration, heat and cold stress following ABA-independent signal transduction pathways (Additional file 11). Only *TaAnn2* and *TaAnn12* genes were significantly induced by drought and salinity stresses (Fig. 5a, d). *TaAnn2* was probably induced through ABA-independent signal pathway, as its promoter does not contain DRE/CRT cis-element. The induction of *TaAnn12* could be due to the presence of ABRE and DRE/CRT motifs and the cross-talk between ABA-dependent and ABA-independent pathways.

As LTRE motifs containing genes, *TaAnn3*, *TaAnn10* and *TaAnn12* were up-regulated but *TaAnn1*, *TaAnn4*, *TaAnn6*, *TaAnn7* and *TaAnn8* expressions were nearly not affected by cold stress (Fig. 5c), indicating that the gene expression level under cold condition was not only dependent on the existence of LTRE element.

### Tissue-preferential expression of annexin genes

Previous studies have demonstrated that annexin genes are expressed in various plant tissues [13, 14, 43]. Comprehensive expression profiles of the annexin gene family throughout the body and lifespan of wheat have not been previously reported. In this study, the expression profiles of ten *TaAnn* genes in different wheat tissues were investigated using real-time PCR (Fig. 6). The results provided valuable information for elucidating the functions of the annexin gene family in wheat. Different annexin genes exhibited diverse expression patterns in wheat. Several annexin genes showed organ-preferential expression patterns, suggesting specific or significant roles *in vivo*. Annexins may play important roles in roots. Transcripts of *Medicago truncatula* and maize annexins have been detected in the root elongation zone [47, 48]. In this study, *TaAnn1* and *TaAnn2* were preferentially expressed in root. The expression pattern of *TaAnn3* in root, stem and leaves was in general agreement with the expression data of *AnnAt1* [43]. The functions of annexins in mature stem, root and flag leaf, which are organs involved in water and nutrient conductance and grain filling, remain unclear. However, annexins in phloem sap were proposed to be involved in the long-distance signal transduction via sensing Ca^2+^ [49]. *TaAnn4*, *TaAnn6*, *TaAnn7* and *TaAnn9* were mainly expressed in the flag leaf (Fig. 6). The flag leaf is the major organ for transitory starch metabolism in this species and its assimilates are the most important contributor to dry weight accumulation in the grains [50]. The function of annexins expressed in the flag leaf is not yet clear. *AtAnn5* related to pollen grains development was specifically expressed in anther [30, 51]. In this study, *TaAnn10* was preferentially expressed in stamen after anthesis and displayed a low level in the spike not including anther, suggesting it may be involved in the process of stamen development or maturation (Fig. 7c).

### Annexin genes expression regulation by abiotic stress and ABA

Accumulating evidences from various plant species including Arabidopsis, rice and tomato have shown the up-regulation of annexin genes in response to abiotic stresses [13, 22, 43]. However, no such information about wheat annexin genes is available. In this research, most of wheat annexin genes showed the induced expression pattern under stress treatments (Fig. 5), which was similar to other plant annexin genes reported. ABA is a phytohormone critical for plant growth and stress response. It plays important roles in integrating various stress signals and controlling downstream stress responses. The mechanisms by which plants respond to stresses include ABA-dependent and ABA-independent processes [40]. In this study, many annexin genes were induced both by ABA and some aboitic stresses, suggesting these genes probably function in stress responses through ABA-dependent pathway (Fig. 5).

### *TaAnn10*, calcium signaling, and cold-induced male sterility in wheat

TGMS breeding lines of wheat are hypersensitive to low temperature during the meiosis stage [34] . Upon stressed by cold, separation of dyads was abnormally, the phragmoplast defectively formed, and the cell plate irregularly assembled during male meiosis I in these lines. Transcriptome profiling revealed that genes playing key roles in the dynamic organization of cytoskeletons were dramatically repressed in cold stressed anther of the TGMS lines [34]. For example, genes encoding profilin and ADF (actin-depolymerizing factor), which act synergistically to regulate actin dynamics [52], were drastically repressed in cold stressed anthers [34]. ADF activity can be regulated by phosphorylation, and a calmodulin-like domain protein kinase (CDPK) was known to phosphorylate ADF in plants [53, 54]. Thus, we speculate that calcium signaling is probably involved in cold induced male sterility of TGMS lines.

Ca^2+^ is a secondary messenger in signaling pathways for a diverse array of stimuli in plants [55, 56]. When exposed to low temperature, plants increase their cytosolic calcium levels, which are mainly mediated by Ca^2+^ influx from the intercellular space [57–59]. The calcium signals are sensed by calcium sensors such as CaMs/CMLs, CDPKs/CCaMKs and CBLs [60]. Annexins act primarily as a putative “linker” between calcium signaling, the actin cytoskeleton, and the membrane. It is intriguing to note in this regard that the expression of *TaAnn10* was preferentially in the stamen and strongly induced by cold treatment in the recovery lines and the F_1_ hybrids but not the TGMS lines (Fig. 8). Further, the transcript level of a specific *CDPK* of wheat, *TaCDPK15* (GenBank accession: EU181186.1), showed no obvious change under salinity, drought and ABA treatments, but was strongly induced by cold stress, which was in consistent with that of *TaAnn10*. We found a burst of *TaCDPK15* transcript abundance prior to that of *TaAnn10* under normal condition in BS366 (Additional file 12). This pattern was enhanced upon cold stress for *TaCDPK15* while *TaAnn10* expression level remained flat. Genetic studies in Arabidopsis has revealed that *AtAnn5* is involved in pollen development, germination and pollen tube growth through the promotion of endomembrane trafficking modulated by calcium [30, 51]. It is possible that *TaAnn10*, which is not orthologous to *AtAnn5* based on sequence similarity (Fig. 2), also participates in relaying cold-induced calcium signals to modulating cytoskeleton dynamics. Together, these results indicate that the strengthened calcium signaling upon cold stress due to failed *TaAnn10* induction could be one of mechanisms underlying temperature sensitive male sterility of TGMS lines. In summary, the current work has opened a new avenue for functional studies of calcium dependent annexin genes in male reproductive development of wheat.

## Conclusions

In this study, 25 and 11 putative annexin genes were identified in wheat and barley, respectively. 8 and 11 annexin genes were identified in *T. urartu* and *A. tauschii*, respectively. Annexins from these four species and some other monocots and eudicots were classified into six different orthologous groups that share similar motif organizations. The nucleotide diversity of each of *Ann1*–*12* genes among *T. aestivum*, *T. urartu*, *A. tauschii* and *H. vulgare* species was very low. *Ann2* gene has been under positive selection, but *Ann6* and *Ann7* have been under purifying selection among the four species in their evolutionary histories.

We found that wheat annexin genes showed differential tissue-specific and abiotic stresses responsive expression patterns. Importantly, *TaAnn10* is preferentially expressed in the anther but fails to be induced by low temperature in thermosensitive genic male sterile lines, suggesting that the specific down-regulation of *TaAnn10* is associated with the conditional male sterility in wheat. In a word, these results provided significant information for understanding the diverse roles of plant annexins and opened a new avenue for functional studies of cold induced male sterility in wheat.

## Methods

### Identification of annexin genes from *T. aestivum*, *H. vulgare*, *T. urartu* and *A. tauschii*

The annexins in Arabidopsis and rice were obtained by searching the corresponding plant genome sequence resources with ‘annexin’ as the keyword. The complete sets of annexin amino acid sequences from Arabidopsis and rice were used to search, with tBLASTn (e-value <10^−4^), for related sequences in the GenBank EST database for *T. aestivum* (Ta.seq.all.gz). The EST sequences were assembled into contigs using CAP3 [61] under high stringency parameters of a minimum sequence identity of 98 %; minimum overlap length of 40 nt; gap penalty, 6; match value, 2; mismatch penalty (−5). An initial set of contigs was used to search the Wheat Survey Sequences (WSS) of the International Wheat Genome Sequencing Consortium [37] in order to identify these full-length annexin sequences or chromosome arm on which the gene was located. Open reading frames and translation of the contigs were carried out with the DNASTAR software (http://www.dnastar.com/) and confirmed by BLASTx with the GenBank NR database. The cDNA of *TaAnn10-A*, not represented in the *T. aestivum* GenBank EST database, was assembled independently from Hiseq2000 (Illumina Solexa) cDNA sequences of *T. aestivum* (unpublished data) and from matches of the homologous, *TaAnn10-B*, to the WSS genomic wheat sequence database (Additional file 13).

Wheat annexins protein sequences were used to search the *H. vulgare*, *T. urartu* and *Aegilops speltoides* database by tBLASTn. The complete genomic sequences and relative coding sequences of annexins gene were acquired in FASTA format and were translated with the DNASTAR software (http://www.dnastar.com/) (Additional file 13). In cases where the original annotation appears to have misidentified the exon/intron junctions or start codons, the flanking sequence for the annotated gene region was reanalyzed and annotated manually by searching for extended ORFs and sequence similarity to the *T. aestivum* protein sequences.

### Conserved motif analysis, subcelluar location and intron-exon structure prediction

Conserved protein motifs of all annexin protein sequences from wheat and barley were analyzed using the MEME 4.6.1 software [62] (http://meme.sdsc.edu/meme/cgi-bin/meme.cgi) with the number of different motifs as 10, minimum motif and maximum motif window set to 6 and 50, respectively. The functional annotations of these motifs were analyzed by InterProScan (http://www.ebi.ac.uk/Tools/pfa/iprscan/) [38] and SMART databases (http://smart.embl-heidelberg.de) [63, 64]. The prediction on subcellular localization of each wheat and barley annexin protein was carried out using the CELLO v2.5 server (http://cello.life.nctu.edu.tw/). The intron/exon structures of wheat annexin genes were determined using GSDS (http://gsds.cbi.pku.edu.cn/) by comparing the full length cDNA sequences to the genomic sequences in the IWGSC’s WSS database (http://www.wheatgenome.org/Tools-and-Resources).

### Putative cis-element analysis in promoter regions

The annexin gene sequences were further used as query sequences for the BLASTN search against SGN wheat whole genome scaffolds data (version 2.40). 2 kb of genomic sequences upstream of 5′-UTR of each annexin gene was obtained for putative cis-element analysis in the PLACE database (http:// www.dna.affrc.go.jp/ PLACE/ signalscan.html).

### Sequence alignment and phylogenetic analysis

Multiple sequence alignments of full-length protein sequences were generated using the Clustal X software (version 1.81). The phylogenetic tree was constructed by the maximum likelihood method using the MEGA 5 software with the following parameters: Poisson model, pairwise deletion and 1000-replicates bootstrap [65]. The annexin sequences from five monocots (*Oryza sativa*, *Zea mays*, *Sorghum bicolor*, *Brachypodium distachyon* and *Setaria italica*) and seven dicots (*Arabidopsis thaliana*, *Medicago truncatula*, *Populus trichocarpa*, *Vitis vinifera*, *Carica papaya*, *Glycine max* and *Cucumis sativus*) were obtained from SUPERFAMILY (http://supfam.cs.bris.ac.uk/SUPERFAMILY/). The orthologs of *Ann1* − *12* genes among *T. aestivuma*, *T. urartu*, *A. tauschii*, *H. vulgare*, *O. sativa* and *B. distachyon* were identified by OrthoMCL (v1.4) software with the *P* value of 1e-20. The genetic variations and selective pressures of *Ann1-12* genes among and within *T. aestivum*, *T. urartu*, *A. tauschii* and *H. vulgare* species were measured by the software of DnaSP 5.0.

### Expression analysis using published microarray data

The expression behaviors of wheat annexin genes were examined in a set of wheat microarray data downloaded from GEO at NCBI (http://www.ncbi.nlm.nih.gov/geo) and the transcriptome data at PLEXdb (http://www.plexdb.org) (Probe Set ID see for Additional file 14). The microarray data of wheat gene expression under drought, obtained from experiment TA43 (GSE30872), were generated by the hybridization of RNA from the root samples of drought tolerant 'Luohan No.2' (LH) and drought susceptible 'Chinese Spring' (CS) after the control and PEG treatments for 6 h. The microarray data of wheat gene expression under salt, which data from experiment E-MEXP-971, were generated by the hybridization of RNA from the root and shoot samples of salt tolerant wheat germplasm lines W4909 and salt susceptible 'Chinese Spring' (CS) after the control and salt treatments for 24 h [66]. The microarray data of wheat gene expression under low temperature, which data from experiment TA42 (GSE23889), were generated by the hybridization of RNA from the samples of Winter Manitou (12 W), Spring Northstar (8S), Spring Manitou (Ma), and Winter Northstar (No) after the cold treatment for 0, 2 14, 21, 35, 42, 56 and 70 days, respectively [67]. For experiment TA43 (GSE12508), the microarray data of selected genes in various tissues such as germinating seed coleoptile, germinating seed root, germinating seed embryo, seedling root, seedling crown, seedling leaf, immature inflorescence, floral bracts before anthesis, pistil before anthesis, anthers before anthesis, 3–5 DAP caryopsis, 22 DAP embryo and 22 DAP endosperm were retrieved from experiments including TA3 experiment [41].

For microarray data analysis, the image (cel) files were imported into the online tool PLEXdb (http://www.plexdb.org). Data normalization was carried out by a script using Bioconductor, and R. Bioconductor is an open source project for microarray data analysis, visualization and annotation. It started in fall of 2001 and quickly evolved into a high-profile project. It provides packages for analyzing both Affymetrix GeneChip data (*affy*, *affydata* and *affycomp* packages) and cDNA spotted microarrays. PLEXdb uses its *affy* package for estimation of expression values by Robust Multi-chip Average (RMA) methods. Heat maps were used to present the expression levels of wheat annexin genes. The maps were generated using Gene Cluster 3.0 (http://softadvice.informer.com/Gene_Cluster_3.0.html). The data were adjusted by median centering of the genes and were clustered by Centroid linkage hierarchical method, and an uncentered Pearson correlation algorithm was used. The result was visualized as a heat map generated by TreeView (http://www.treeview.net/tv/download.asp).

### Plant materials and treatments

Seeds of wheat line Jinghua 9 were used in this study. Tissue-specific expression patterns of annexin genes were analyzed in 7- and 14-day-old root, stem, leaf and stamen before flowering. To analyze the abiotic stress responses, seeds of Jinghua 9 were germinated and well planted in greenhouse at 25 °C with a photoperiod of 16 h light/8 h dark. For salt treatment, 14-day-old seedlings were treated with 250 mM NaCl solution. For drought stress, the seedlings were incubated in 25 % (w/v) PEG-6000 solution. The seedlings were grown at 4 °C for cold stress. For ABA treatment, the seedlings were sprayed with 0.1 mM ABA solution. For each treatment mentioned above, seedlings were treated for 0, 1, 2, 10 and 24 h. Control seedlings were exposed to none of these treatments. After treatment, all samples were immediately frozen in liquid nitrogen and stored at - 80 °C.

Two wheat TGMS lines BS366 (BS366-2-8) and BS400 (BS400-640), two recovery lines GLSD and Taiyuan806, and the two hybrid lines BS366 × GLDS and BS366 × Taiyuan806 were provided and stored by our labs. The sterilities of BS366 and BS400 were affected by temperature and photoperiod. Plants of BS366, BS400, GLSD, Taiyuan806, BS366 × GLDS and BS366 × Taiyuan806 were cultivated in fields under fertile (high-temperature and long-photoperiod zone, comprising 20 °C with a 12 h photoperiod) and sterile (low-temperature and short-photoperiod zone, comprising 10 °C with a 10 h photoperiod) conditions. Their various organs such as root, stem, leaf, anther, spike (not including anther) at the stamen and pistil initiation stage, anther separation stage, meiosis stage, uninucleate stage and 12DAP immature seed were collected. The anthers of these plants were stained with I2-KI.

### RNA isolation and real-time PCR

Real-time PCR was performed to confirm the differential expression of representative wheat annexin genes using gene-specific primers (Additional file 15). Total RNA was isolated using TRIzol reagent (Bio Basic Inc, Canada). First-strand cDNA was synthesized using reverse transcriptase (Takara, China) according to the manufacturer’s instructions. Real-time PCR analysis was conducted using an Eco Real-Time PCR system (Illumina, San Diego,CA, USA) with TaKaRa SYBR® Premix Ex Taq™ (Tli RNase H Plus) (TaKaRa, Dalian, China). The expression level of wheat actin gene (GenBank accession: 542814) was used as the internal control. Real-time PCR was performed in a 48-well plate using Eco Real-Time PCR Technology (Illumina). The thermal cycling conditions of 95 °C for 30 s followed by 40 cycles of 95 °C for 5 s, 58 °C for 30 s were used. Then melting curve of 95 °C for 15 s, 60 °C for 60 s and 95 °C 15 s were used. The relative gene expression levels were analyzed according to the 2^−ΔΔCT^ method [68].

## Abbreviations

ABA, abscisic acid; ESTs, expressed sequence tags; Real-time PCR, quantitative real-time PCR; TGMS, thermosensitive genic male sterile; WSS, whole genome survey sequences

## Additional files

Additional file 1: Table S1. Information on annexin sequences identified in the *T.aestivum*, *T.urartu*, *A.tauschii* and *H.vulgare*. (PDF 40 kb) Additional file 2: Table S2. Description of *T.aestivum*, *T.urartu*, *A.tauschii* and *H.vulgare* annexin genes. (PDF 46 kb) Additional file 3: Figure S1. Multiple sequence alignment of deduced amino acid sequences of 25 wheat annexin genes with rice annexin *OsAnn2* (Os05g31760) obtained by ClustalW. (PDF 212 kb) Additional file 4: Table S3. Conserved sequence motifs identified in the amino acid sequences of in wheat, *T.urartu*, *A.tauschii*, barley and rice as analyzed by MEME tools. (PDF 7 kb) Additional file 5: Figure S2. Phylogenetic analysis and predicted structure of annexin proteins of 9 monocots plant and 7 discots plant. (PDF 484 kb) Additional file 6: Table S4. Orthologous groups of annexin genes in *T.aestivuma*, *T.urartu*, *A.tauschii*, *H. vulgare*, *O.sativa* and *B.distachyon*. (PDF 115 kb) Additional file 7: Table S5 Orthologous groups of annexin genes in *T.aestivuma*, *T.urartu*, *A.tauschii*, *H. vulgare*, *O.sativa* and *B.distachyon*. (PDF 101 kb) Additional file 8: Figure S3. Phylogenetic analysis of annexin protein sequences from three monocots (wheat, barely and rice) and two dicots (*Arabidopsis thaliana* and *Glycine max*). (PDF 65 kb) Additional file 9: Figure S4. Sequence aligment and logos of four annexin domains in the dicots and monocots. (PDF 1110 kb) Additional file 10: Figure S5. Gene Structure analysis of Arabidopsis, rice, barley and wheat annexin genes. (PDF 65 kb) Additional file 11: Table S6. Putative cis-elements in the 2 kb upstream promoter region of translation start site in wheat annexin genes. (PDF 13 kb) Additional file 12: Figure S6. Expression of *TaAnn10* and *TaCDPK15* in male sterile line BS366 under sterile and fertile condition. (PDF 129 kb) Additional file 13: Full set of annexin sequences from wheat (*T. aestivum*), barely (*H.vulgare*), *T.urartu* and *A.tauschii*. (PDF 208 kb) Additional file 14: Table S7. Identifiers for annexin genes on Microarrays in Wheat. (PDF 81 kb) Additional file 15: Table S8. Primer pairs used for the expression analysis of wheat annexin genes by quantitative RT-PCR. (PDF 18 kb)

## References

[1] Laohavisit A, Davies JM (2011). Annexins. New Phytol.

[2] Mortimer JC, Laohavisit A, Macpherson N, Webb A, Brownlee C, Battey NH, Davies JM (2008). Annexins: multifunctional components of growth and adaptation. J Exp Bot.

[3] Gerke V, Moss SE (2002). Annexins: from structure to function. Physiol Rev.

[4] Konopka-Postupolska D, Clark G, Hofmann A (2011). Structure, function and membrane interactions of plant annexins: an update. Plant Sci Int J Exp Plant Biol.

[5] Laohavisit A, Brown AT, Cicuta P, Davies JM (2010). Annexins: components of the calcium and reactive oxygen signaling network. Plant Physiol.

[6] Lee S, Lee EJ, Yang EJ, Lee JE, Park AR, Song WH, Park OK (2004). Proteomic identification of annexins, calcium-dependent membrane binding proteins that mediate osmotic stress and abscisic acid signal transduction in Arabidopsis. Plant Cell.

[7] Clark GB, Dauwalder M, Roux SJ (1998). Immunological and biochemical evidence for nuclear localization of annexin in peas. Plant Physiol Biochem.

[8] Huang Y, Wang J, Zhang L, Zuo K (2013). A Cotton Annexin Protein AnxGb6 Regulates Fiber Elongation through Its Interaction with Actin 1. PLoS One.

[9] Huang Y, Deng T, Zuo K (2013). Cotton annexin proteins participate in the establishment of fiber cell elongation scaffold. Plant Signal Behav.

[10] Riewe D, Grosman L, Zauber H, Wucke C, Fernie AR, Geigenberger P (2008). Metabolic and Developmental Adaptations of Growing Potato Tubers in Response to Specific Manipulations of the Adenylate Energy Status. Plant Physiol.

[11] Baucher M, Oukouomi Lowe Y, Vandeputte OM, Mukoko Bopopi J, Moussawi J, Vermeersch M, Mol A, El Jaziri M, Homblé F, Pérez-Morga D (2011). Ntann12 annexin expression is induced by auxin in tobacco roots. J Exp Bot.

[12] Seals DF, Randall SK (1997). A Vacuole-Associated Annexin Protein, VCaB42, Correlates with the Expansion of Tobacco Cells. Plant Physiol.

[13] Jami S, Clark G, Ayele B, Roux S, Kirti PB (2012). Identification and characterization of annexin gene family in rice. Plant Cell Rep.

[14] Zhou M-L, Yang X-B, Zhang Q, Zhou M, Zhao E-Z, Tang Y-X, Zhu X-M, Shao J-R, Wu Y-M (2013). Induction of annexin by heavy metals and jasmonic acid in Zea mays. Funct Integr Genomics.

[15] Breton G, Vazquez-Tello A, Danyluk J, Sarhan F (2000). Two novel intrinsic annexins accumulate in wheat membranes in response to low temperature. Plant Cell Physiol.

[16] Peng Z, Wang M, Li F, Lv H, Li C, Xia G (2009). A Proteomic Study of the Response to Salinity and Drought Stress in an Introgression Strain of Bread Wheat. Mol Cell Proteomics.

[17] Hofmann A, Proust J, Dorowski A, Schantz R, Huber R (2000). Annexin 24 from Capsicum annuum: X-ray structure and biochemical characterization. J Biol Chem.

[18] Kovacs I, Ayaydin F, Oberschall A, Ipacs I, Bottka S, Pongor S, Dudits D, Toth EC (1998). Immunolocalization of a novel annexin-like protein encoded by a stress and abscisic acid responsive gene in alfalfa. Plant J.

[19] Cantero A, Barthakur S, Bushart TJ, Chou S, Morgan RO, Fernandez MP, Clark GB, Roux SJ (2006). Expression profiling of the Arabidopsis annexin gene family during germination, de-etiolation and abiotic stress. Plant Physiol Biochem.

[20] Jami SK, Dalal A, Divya K, Kirti PB (2009). Molecular cloning and characterization of five annexin genes from Indian mustard (Brassica juncea L. Czern and Coss). Plant Physiol Biochem.

[21] Jami SK, Clark GB, Turlapati SA, Handley C, Roux SJ, Kirti PB (2008). Ectopic expression of an annexin from Brassica juncea confers tolerance to abiotic and biotic stress treatments in transgenic tobacco. Plant Physiol Biochem.

[22] Lu Y, Ouyang B, Zhang J, Wang T, Lu C, Han Q, Zhao S, Ye Z, Li H (2012). Genomic organization, phylogenetic comparison and expression profiles of annexin gene family in tomato (Solanum lycopersicum). Gene.

[23] Carroll AD, Moyen C, Van Kesteren P, Tooke F, Battey NH, Brownlee C (1998). Ca2+, annexins, and GTP modulate exocytosis from maize root cap protoplasts. Plant Cell.

[24] Shin H, Brown RM (1999). GTPase activity and biochemical characterization of a recombinant cotton fiber annexin. Plant Physiol.

[25] Clark GB, Turnwald S, Tirlapur UK, Haas CJ, von der Mark K, Roux SJ, Scheuerlein R (1995). Polar distribution of annexin-like proteins during phytochrome-mediated initiation and growth of rhizoids in the ferns Dryopteris and Anemia. Planta.

[26] Dai S, Li L, Chen T, Chong K, Xue Y, Wang T (2006). Proteomic analyses of Oryza sativa mature pollen reveal novel proteins associated with pollen germination and tube growth. Proteomics.

[27] Konopka-Postupolska D, Clark G, Goch G, Debski J, Floras K, Cantero A, Fijolek B, Roux S, Hennig J (2009). The role of annexin 1 in drought stress in Arabidopsis. Plant Physiol.

[28] Clark G, Konopka-Postupolska D, Hennig J, Roux S (2010). Is annexin 1 a multifunctional protein during stress responses?. Plant Signal Behav.

[29] Huh SM, Noh EK, Kim HG, Jeon BW, Bae K, Hu HC, Kwak JM, Park OK (2010). Arabidopsis annexins AnnAt1 and AnnAt4 interact with each other and regulate drought and salt stress responses. Plant Cell Physiol.

[30] Zhu J, Wu X, Yuan S, Qian D, Nan Q, An L, Xiang Y (2014). Annexin5 plays a vital role in Arabidopsis pollen development via Ca2 + −dependent membrane trafficking. PLoS One.

[31] Divya K, Jami SK, Kirti PB (2010). Constitutive expression of mustard annexin, AnnBj1 enhances abiotic stress tolerance and fiber quality in cotton under stress. Plant Mol Biol.

[32] Tang W, He Y, Tu L, Wang M, Li Y, Ruan YL, Zhang X (2014). Down-regulating annexin gene GhAnn2 inhibits cotton fiber elongation and decreases Ca2+ influx at the cell apex. Plant Mol Biol.

[33] Zhao C (2013). Research and application of hybrid wheat in China. Eng Sci.

[34] Tang Z, Zhang L, Yang D, Zhao C, Zheng Y (2011). Cold stress contributes to aberrant cytokinesis during male meiosis I in a wheat thermosensitive genic male sterile line. Plant Cell Environ.

[35] Xu C, Liu Z, Zhang L, Zhao C, Yuan S, Zhang F (2013). Organization of actin cytoskeleton during meiosis I in a wheat thermo-sensitive genic male sterile line. Protoplasma.

[36] Huang S, Sirikhachornkit A, Su X, Faris J, Gill B, Haselkorn R, Gornicki P (2002). Genes encoding plastid acetyl-CoA carboxylase and 3-phosphoglycerate kinase of the Triticum/Aegilops complex and the evolutionary history of polyploid wheat. Proc Natl Acad Sci U S A.

[37] Gill BS, Appels R, Botha-Oberholster AM, Buell CR, Bennetzen JL, Chalhoub B, Chumley F, Dvorak J, Iwanaga M, Keller B (2004). A workshop report on wheat genome sequencing: International Genome Research on Wheat Consortium. Genetics.

[38] Finn RD, Bateman A, Clements J, Coggill P, Eberhardt RY, Eddy SR, Heger A, Hetherington K, Holm L, Mistry J (2014). Pfam: the protein families database. Nucleic Acids Res.

[39] Vogel C, Chothia C (2006). Protein family expansions and biological complexity. PLoS Comput Biol.

[40] Nakashima K, Ito Y, Yamaguchi-Shinozaki K (2009). Transcriptional regulatory networks in response to abiotic stresses in Arabidopsis and grasses. Plant Physiol.

[41] Schreiber AW, Sutton T, Caldo RA, Kalashyan E, Lovell B, Mayo G, Muehlbauer GJ, Druka A, Waugh R, Wise RP (2009). Comparative transcriptomics in the Triticeae. BMC Genomics.

[42] Gorecka KM, Konopka-Postupolska D, Hennig J, Buchet R, Pikula S (2005). Peroxidase activity of annexin 1 from Arabidopsis thaliana. Biochem Biophys Res Commun.

[43] Clark GB, Sessions A, Eastburn DJ, Roux SJ (2001). Differential expression of members of the annexin multigene family in Arabidopsis. Plant Physiol.

[44] Hofmann A, Delmer DP, Wlodawer A (2003). The crystal structure of annexin Gh1 from Gossypium hirsutum reveals an unusual S3 cluster. Eur J Biochem.

[45] Englbrecht CC, Schoof H, Bohm S (2004). Conservation, diversification and expansion of C2H2 zinc finger proteins in the Arabidopsis thaliana genome. BMC Genomics.

[46] Clark GB, Morgan RO, Fernandez MP, Roux SJ (2012). Evolutionary adaptation of plant annexins has diversified their molecular structures, interactions and functional roles. New Phytol.

[47] Bassani M, Neumann PM, Gepstein S (2004). Differential expression profiles of growth-related genes in the elongation zone of maize primary roots. Plant Mol Biol.

[48] De Carvalho-Niebel F, Timmers AC, Chabaud M, Defaux-Petras A, Barker DG (2002). The Nod factor-elicited annexin MtAnn1 is preferentially localised at the nuclear periphery in symbiotically activated root tissues of Medicago truncatula. Plant J.

[49] Giavalisco P, Kapitza K, Kolasa A, Buhtz A, Kehr J (2006). Towards the proteome of Brassica napus phloem sap. Proteomics.

[50] Serrago RA, Alzueta I, Savin R, Slafer GA (2013). Understanding grain yield responses to source–sink ratios during grain filling in wheat and barley under contrasting environments. Field Crop Res.

[51] Zhu J, Yuan S, Wei G, Qian D, Wu X, Jia H, Gui M, Liu W, An L, Xiang Y (2014). Annexin5 is essential for pollen development in Arabidopsis. Mol Plant.

[52] Staiger CJ, Blanchoin L (2006). Actin dynamics: old friends with new stories. Curr Opin Plant Biol.

[53] Smertenko AP, Jiang CJ, Simmons NJ, Weeds AG, Davies DR, Hussey PJ (1998). Ser6 in the maize actin-depolymerizing factor, ZmADF3, is phosphorylated by a calcium-stimulated protein kinase and is essential for the control of functional activity. Plant J.

[54] Allwood EG, Smertenko AP, Hussey PJ (2001). Phosphorylation of plant actin-depolymerising factor by calmodulin-like domain protein kinase. FEBS Lett.

[55] Pineros M, Tester M (1997). Calcium channels in higher plant cells: selectivity, regulation and pharmacology. J Exp Bot.

[56] Sanders D, Pelloux J, Brownlee C, Harper JF (2002). Calcium at the Crossroads of Signaling. Plant Cell.

[57] Knight MR, Campbell AK, Smith SM, Trewavas AJ (1991). Transgenic plant aequorin reports the effects of touch and cold-shock and elicitors on cytoplasmic calcium. Nature.

[58] Knight H, Trewavas AJ, Knight MR (1996). Cold calcium signaling in Arabidopsis involves two cellular pools and a change in calcium signature after acclimation. Plant Cell.

[59] Knight H, Trewavas AJ, Knight MR (1997). Calcium signalling in Arabidopsis thaliana responding to drought and salinity. Plant J.

[60] Kudla J, Batistič O, Hashimoto K (2010). Calcium Signals: The Lead Currency of Plant Information Processing. Plant Cell.

[61] Huang X, Madan A (1999). CAP3: A DNA sequence assembly program. Genome Res.

[62] Bailey TL, Johnson J, Grant CE, Noble WS (2015). The MEME Suite. Nucleic Acids Res.

[63] Schultz J, Copley RR, Doerks T, Ponting CP, Bork P (2000). SMART: a web-based tool for the study of genetically mobile domains. Nucleic Acids Res.

[64] Letunic I, Goodstadt L, Dickens NJ, Doerks T, Schultz J, Mott R, Ciccarelli F, Copley RR, Ponting CP, Bork P (2002). Recent improvements to the SMART domain-based sequence annotation resource. Nucleic Acids Res.

[65] Tamura K, Peterson D, Peterson N, Stecher G, Nei M, Kumar S (2011). MEGA5: molecular evolutionary genetics analysis using maximum likelihood, evolutionary distance, and maximum parsimony methods. Mol Biol Evol.

[66] Mott IW, Wang RRC (2007). Comparative transcriptome analysis of salt-tolerant wheat germplasm lines using wheat genome arrays. Plant Sci.

[67] Laudencia-Chingcuanco D, Ganeshan S, You F, Fowler B, Chibbar R, Anderson O (2011). Genome-wide gene expression analysis supports a developmental model of low temperature tolerance gene regulation in wheat (Triticum aestivum L.). BMC Genomics.

[68] Livak KJ, Schmittgen TD (2001). Analysis of relative gene expression data using real-time quantitative PCR and the 2(−Delta Delta C(T)) Method. Methods.

