# Supplementary material for: Comprehensive analyses of the annexin gene family in wheat
Source: BMC Genomics. 2016 May 28;17:415. doi: 10.1186/s12864-016-2750-y (PMC4884362; doi:10.1186/s12864-016-2750-y)
Supplement: Additional file 1: Table S1. — Information on annexin sequences identified in the T.aestivum, T.urartu, A.tauschii and H.vulgare. (PDF 40 kb) [file 12864_2016_2750_MOESM1_ESM.pdf]

Additional file 1: Table S1. Information on annexin sequences identified in the *T.aestivum*, *T.urartu A.tauschii* and *H.vulgare.*

			T.aestivum			*T.urartu*		*A.tauschii*		*H.vulgare*		
**Gene**		**IWGSC Chromosome Localization**	**Unigene**	**GenBank**	**IWGSC**	**Gene**	**Gramene**	**Gene**	**Gramene**	**Gene**	**Gramene**	**GenBank**
TaAnn1	*TaAnn1-A*	1AS	Ta.9484	AY462115.1	Traes_1AS_3264B32DD1.1	*TuAnn1*	TRIUR3_22191	*AeAnn1*	AEGTA07604	*HvAnn1*	MLOC_15770.2	
	TaAnn1-D	1DS			Traes_1DS_DD0F4640E.1							
TaAnn2	*TaAnn2-A*	1AL	Ta.37581		Traes_1AL_A5FB19E3C.1	*TuAnn2*	TRIUR3_11010			*HvAnn2*	MLOC_54933.3	
	TaAnn2-B	1BL			Traes_1BL_E0E972134.1							
TaAnn3	*TaAnn3-A*	1?	Ta.13303			*TuAnn3*	TRIUR3_11009	*AeAnn3*	AEGTA29052	*HvAnn3*	MLOC_54932.1	
	TaAnn3-B	1BL			Traes_1BL_04EBF6A19.1							
TaAnn4	*TaAnn4-A*	2AL			Traes_2AL_788CA6B69.1		-	*AeAnn4*	AEGTA21287		-	
	TaAnn4-D	2DL			Traes_2DL_227971FCE.1							
TaAnn5	*TaAnn5-B*	2BS			Traes_2BS_879851C74.1			*AeAnn5*	AEGTA42363	*HvAnn5*	MLOC_15543.3	
TaAnn6	*TaAnn6-A*	3AL		AAR25142.1	Traes_3AL_A966AD43D.2	*TuAnn6*	TRIUR3_18622	*AeAnn6*	AEGTA16509	*HvAnn6*	MLOC_55134.1	
	TaAnn6-B	3B?			Traes_3B_93504D484.1							
TaAnn7	*TaAnn7-B*	4BL			Traes_4BL_8BD1B62C8.2			*AeAnn7*	AEGTA11977			
	TaAnn7-D	4DL			Traes_4DL_D0B60B7B5.2							
TaAnn8	*TaAnn8-A*	4AS	Ta.37581		Traes_4AS_63C8858E9.1					*HvAnn8*		AK370408
	TaAnn8-D	4DL	Ta.37581		Traes_4DL_87FDF914B.1							
TaAnn9	*TaAnn9-B*	5BL			Traes_5BL_56B9E776F.1			*AeAnn9*	AEGTA10144			
	TaAnn9-D	5DL	Ta.13966	AK332757.1	Traes_5DL_E1D06F29F.1							
TaAnn10	*TaAnn10-A*	5?		KT198661		*TuAnn10*	TRIUR3_04012	*AeAnn10*	AEGTA28110	*HvAnn10*	MLOC_54650.4	

	TaAnn10-B	5BL			Traes_5BL_23101F4FA.2							
	TaAnn10-D	5DL	Ta.63246		Traes_5DL_7A3A1FDF9.2							
	TaAnn11-A	6AL			Traes_6AL_CFE60316E.1							
TaAnn11	*TaAnn11-B*	6?	Ta.1577	AK331181		*TuAnn11*	TRIUR3_16577	*AeAnn11*	AEGTA31729	*HvAnn11*	MLOC_51591.2	
	TaAnn11-D	6DL			Traes_6DL_6F7AB41471.1							
TaAnn12	*TaAnn12-A*	7AL	Ta.40948	AK331881.1	Traes_7AS_FFB7CAFC3.1	*TuAnn12*	TRIUR3_03306	*AeAnn12*	AEGTA27331	*HvAnn12*		AK248515
	TaAnn12-D	7DL			Traes_7DS_3F6DCEAA8.1							

Spaces: not found
