# Supplementary material for: Comprehensive analyses of the annexin gene family in wheat
Source: BMC Genomics. 2016 May 28;17:415. doi: 10.1186/s12864-016-2750-y (PMC4884362; doi:10.1186/s12864-016-2750-y)
Supplement: Additional file 2: Table S2. — Description of T.aestivum, T.urartu, A.tauschii and H.vulgare annexin genes. (PDF 46 kb) [file 12864_2016_2750_MOESM2_ESM.pdf]

Additional file 2: **Table S2.** Description of *T.aestivum*, *T.urartu*, *A.tauschii* and *H.vulgare annexin* genes.

Species	Gene	AA	pI/MW	Aliphatic index	Grand average of hydropathicity	Subcellular localization
	TaAnn1-A	308	9.16/34828.8	86.88	-0.286	Cytoplasmic
	TaAnn1-D	316	9.01/35404.3	86.23	-0.343	Cytoplasmic
	TaAnn2-A	362	6.68/39734.3	89.01	-0.278	Cytoplasmic
	TaAnn2-B	361	6.64/39688.2	88.7	-0.283	Cytoplasmic
	TaAnn3-A	315	6.60/34774.7	84.98	-0.261	Cytoplasmic
	TaAnn3-B	315	6.47/34837.7	84.03	-0.283	Cytoplasmic
	TaAnn4-A	286	5.28/31565.7	92.83	-0.24	Cytoplasmic
T.aestivum	*TaAnn4-D*	315	5.75/34656.3	92.41	-0.236	Cytoplasmic
	TaAnn5-B	308	6.2/34549.4	91.46	-0.269	Cytoplasmic
	TaAnn6-A	316	9.31/35654.7	89.02	-0.307	Cytoplasmic
	TaAnn6-A	316	9.01/35438.3	83.77	-0.359	Cytoplasmic
	TaAnn6-B	316	9.4/35660.8	89.65	-0.304	Cytoplasmic
	TaAnn7-B	319	6.68/35671.6	90.6	-0.321	Cytoplasmic
	TaAnn7-D	319	7.06/35449.4	90.63	-0.288	Cytoplasmic
	TaAnn8-A	367	9.22/40442.0	77.06	-0.443	Cytoplasmic

	TaAnn8-D	363	9.22/40169.6	75.21	-0.5	Cytoplasmic
	TaAnn9-B	315	8.95/35436.5	81.21	-0.435	Periplasmic
	TaAnn9-D	315	8.95434.4	82.13	-0.437	Periplasmic
	TaAnn10-A	322	8.9/36719.9	81.96	-0.47	Cytoplasmic
	TaAnn10-B	330	9.09/37585.9	80.55	-0.501	Cytoplasmic
	TaAnn10-D	330	8.9/37509.8	82.33	-0.465	Cytoplasmic
	TaAnn11-A	315	6.15/35388.1	88.38	-0.395	Cytoplasmic
	TaAnn11-B	315	6.36/35372.1	88.73	-0.39	Cytoplasmic
	TaAnn11-D	315	6.36/35332.1	87.46	-0.398	Cytoplasmic
	TaAnn12-A	314	6.81/35327.1	90.16	-0.428	Cytoplasmic
	TaAnn12-D	314	6.81/35239	90.19	-0.425	Cytoplasmic
T.urartu	*TuAnn1*	285	6.72/31261.5	82.21	-0.249	Cytoplasmic
	TuAnn2	328	7.67/35713.8	91.4	-0.211	Cytoplasmic
	TuAnn3	315	6.93/34894.8	85.3	-0.267	Cytoplasmic
	TuAnn6	291	9.82/32494.6	96.63	-0.095	Cytoplasmic
	TuAnn9	339	8.75/38234.7	84.37	-0.411	Cytoplasmic
	TuAnn10	323	9.09/36876.0	78.39	-0.525	Cytoplasmic
	TuAnn11	312	6.15/34999.7	89.23	-0.397	Cytoplasmic
	TuAnn12	314	6.81/35327.1	90.16	-0.428	Cytoplasmic
	AeAnn1	276	9.07/30764.1	84.2	-0.322	Cytoplasmic

A.tauschii	*AeAnn2*	346	7.20/38236.7	90.55	-0.234	Cytoplasmic
	AeAnn3	315	6.58/34814.7	84.35	-0.263	Cytoplasmic
	AeAnn4	317	5.62/34874.5	91.51	-0.246	Cytoplasmic
	AeAnn5	338	9.21/38360.5	69.59	-0.572	Cytoplasmic
	AeAnn6	170	9.01/19146.9	94.18	-0.231	Cytoplasmic
	AeAnn7	320	6.48/35633.6	87.88	-0.304	Cytoplasmic
	AeAnn9	339	8.75/38206.7	84.37	-0.412	Cytoplasmic
	AeAnn10	330	8.9/37509.8	82.33	-0.465	Cytoplasmic
	AeAnn11	315	6.36/35332.1	87.46	-0.398	Cytoplasmic
	AeAnn12	344	6.43/38657.0	92.53	-0.331	Cytoplasmic
H.vulgare	*HvAnn1*	316	8.87/35582.7	91.11	-0.292	Cytoplasmic
	HvAnn2	362	6.47/39842.6	90.86	-0.227	Cytoplasmic
	HvAnn3	316	6.47/ 34985.8	82.53	-0.31	Cytoplasmic
	HvAnn5	327	7.60/ 36977.1	87.68	-0.319	Cytoplasmic
	HvAnn6	373	9.82/41824.8	86.14	-0.353	Cytoplasmic
	HvAnn7	359	7.66/39782.4	85.96	-0.351	Cytoplasmic
	HvAnn8	363	9.22/39935.3	74.96	-0.455	Cytoplasmic
	HvAnn9	315	8.95/35421.5	80.89	-0.434	Periplasmic
	HvAnn10	321	8.9/36641.7	79.13	-0.504	Cytoplasmic
	HvAnn11	315	6.57/35498.3	89.62	-0.371	Cytoplasmic
	HvAnn12	314	6.52/35246.0	92.36	-0.395	Cytoplasmic

AA, protein length (number of amino acid); pI, theoretical isoelectric point; MW, molecular weight
