# Supplementary material for: Comprehensive analyses of the annexin gene family in wheat
Source: BMC Genomics. 2016 May 28;17:415. doi: 10.1186/s12864-016-2750-y (PMC4884362; doi:10.1186/s12864-016-2750-y)
Supplement: Additional file 3: Figure S1. — Multiple sequence alignment of deduced amino acid sequences of 25 wheat annexin genes with rice annexin OsAnn2 (Os05g31760) obtained by ClustalW. (PDF 212 kb) [file 12864_2016_2750_MOESM3_ESM.pdf]

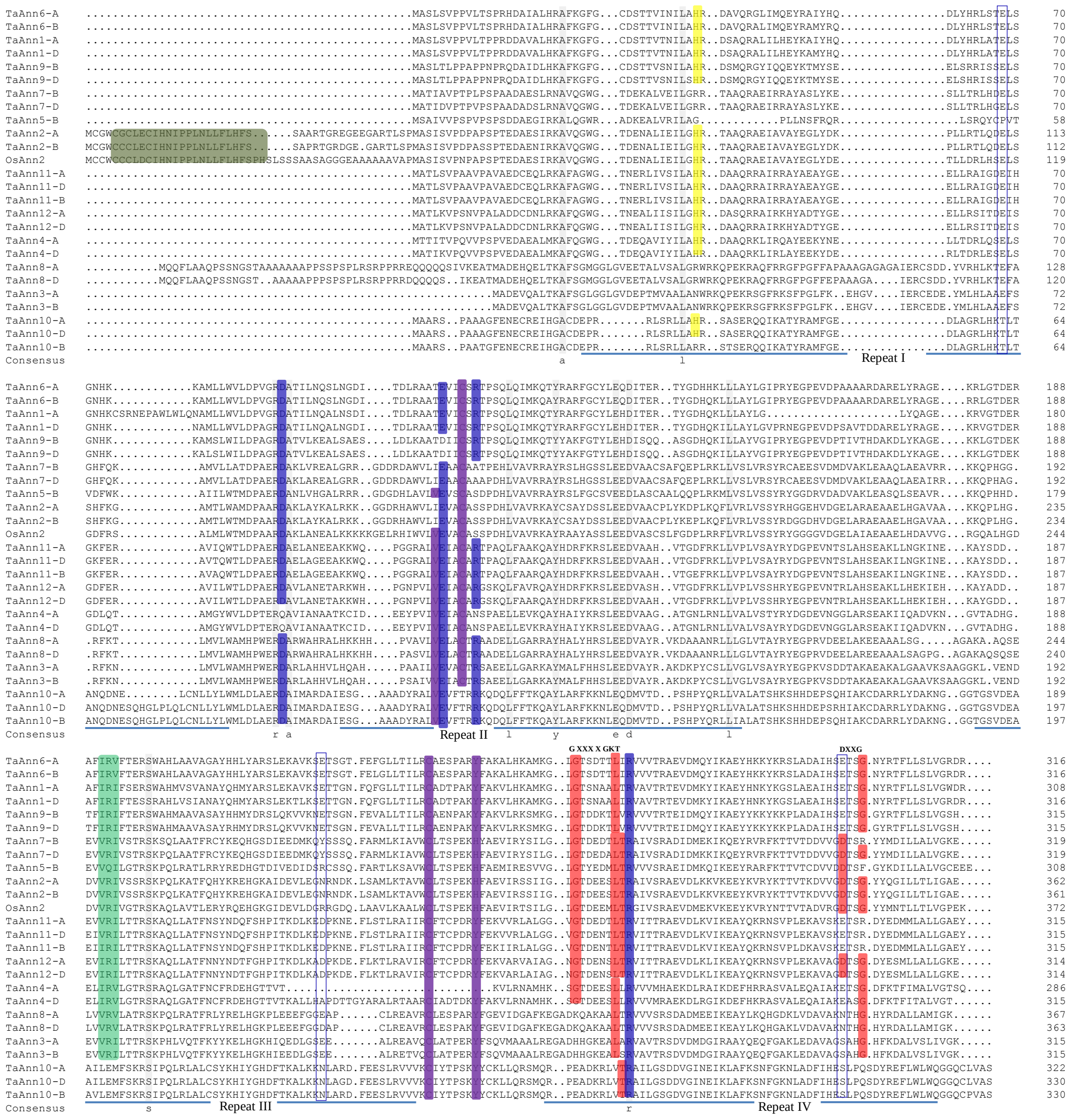

Additional file 3: Figure S1. Multiple sequence alignment of deduced amino acid sequences of 25 wheat annexin genes with rice annexin *OsAnn2* (Os05g31760) obtained by ClustalW. The conserved annexin repeats (Repeat 1 to Repeat 4) are shown beneath the sequence alignments. The sequences highlighted are as follows; olive the two Cys and two His residues represented in bold in Zinc finger C2H2 type domain signature in *OsAnn2*, *TaAnn2-A* and *TaAnn2-B*; yellow conserved His residue; blue salt bridges suggested to be involved in ion channel function; pink conserved residues in S3 cluster that are involved in regulating redox reactions; green IRI motif involved in F-actin binding; red putative GTP binding motifs; the type-II calcium binding motifs are boxed in blue; Light grey and letters indicates the conserved

residues in all the 25 deduced sequences. Point indicate the sequence gaps to optimize the alignment (color figure online).
