# Supplementary material for: Comprehensive analyses of the annexin gene family in wheat
Source: BMC Genomics. 2016 May 28;17:415. doi: 10.1186/s12864-016-2750-y (PMC4884362; doi:10.1186/s12864-016-2750-y)
Supplement: Additional file 4: Table S3. — Conserved sequence motifs identified in the amino acid sequences of in wheat, T.urartu, A.tauschii, barley and rice as analyzed by MEME tools. (PDF 7 kb) [file 12864_2016_2750_MOESM4_ESM.pdf]

Additional file 4: Table S3. Conserved sequence motifs identified in the amino acid sequences of in wheat, *T.urartu*, *A.tauschii*, barley and rice as analyzed by MEME tools. The motif numbers correspond to the colored boxes in Figure 1.

Motif	Width	Multilevel consensus sequence
1	50	LTRVIVTRAEVDMKYIKQEYQKRFKVPLEDDVHGDTSGNYQDFLLSLVGS
2	41	MATISVPPPVPSPREDCINLHKAFQGWGCNETTIINILGHR
3	34	EFIRIFTTRSWPQLRATFNCYNHMHGHPITKDLK
4	50	GWHPDNWVLVEIICSRTPSQLFIMRQAYHARFGCSLEEDVAYCVTGDHQK
5	41	NPSGNFEFMLRTIVWCFTCPDKYFCKVIRKSMKGLGTDENT
6	29	EISGNFCNAMILWMMDPWERDATMANEAL
7	21	LLVPLVSSYRYEGPEVNPDLA
8	21	DAAQRQQIRQHYATMYGEDLL
9	50	ELTKAFSGMGGLGVDEPTMVSALANWRKQPEKRSGFRKSFPGFFKPHGVI
10	15	EAKQLYEAINKKKGP
