# Supplementary material for: Comprehensive analyses of the annexin gene family in wheat
Source: BMC Genomics. 2016 May 28;17:415. doi: 10.1186/s12864-016-2750-y (PMC4884362; doi:10.1186/s12864-016-2750-y)
Supplement: Additional file 5: Figure S2. — Phylogenetic analysis and predicted structure of annexin proteins of 9 monocots plant and 7 discots plant. (PDF 484 kb) [file 12864_2016_2750_MOESM5_ESM.pdf]

A

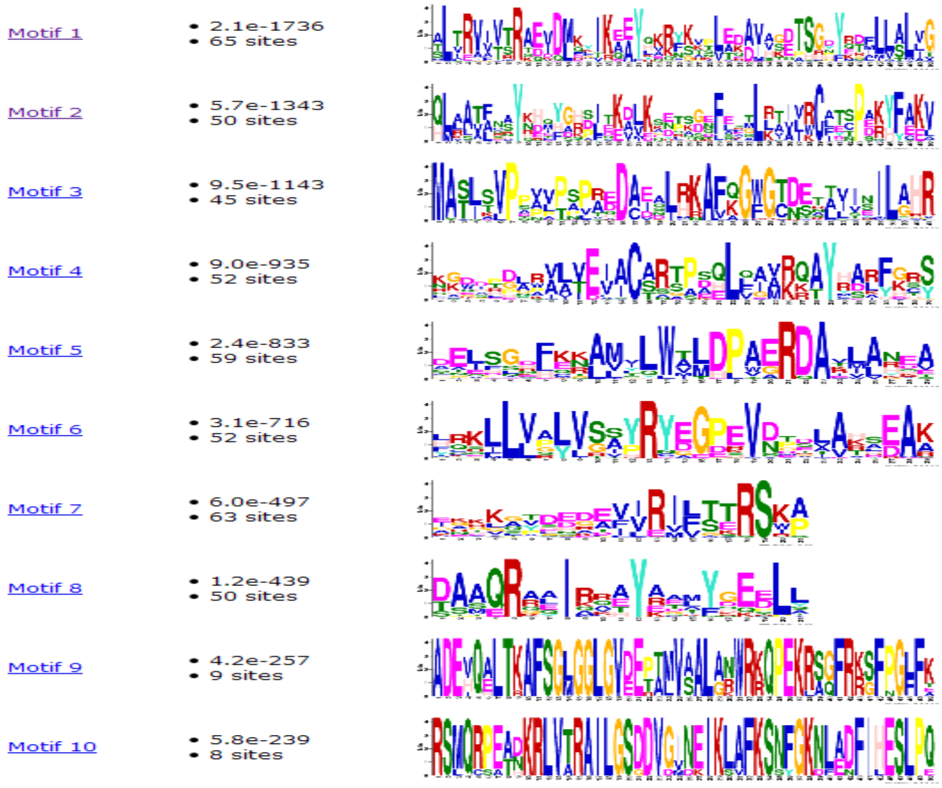
Motif 1
2.1e-1736
65 sites
Motif 2
5.7e-1343
50 sites
Motif 3
9.5e-1143
45 sites
Motif 4
9.0e-935
52 sites
Motif 5
2.4e-833
59 sites
Motif 6
3.1e-716
52 sites
Motif 7
6.0e-497
63 sites
Motif 8
1.2e-439
50 sites
Motif 9
4.2e-257
9 sites
Motif 10
5.8e-239
8 sites

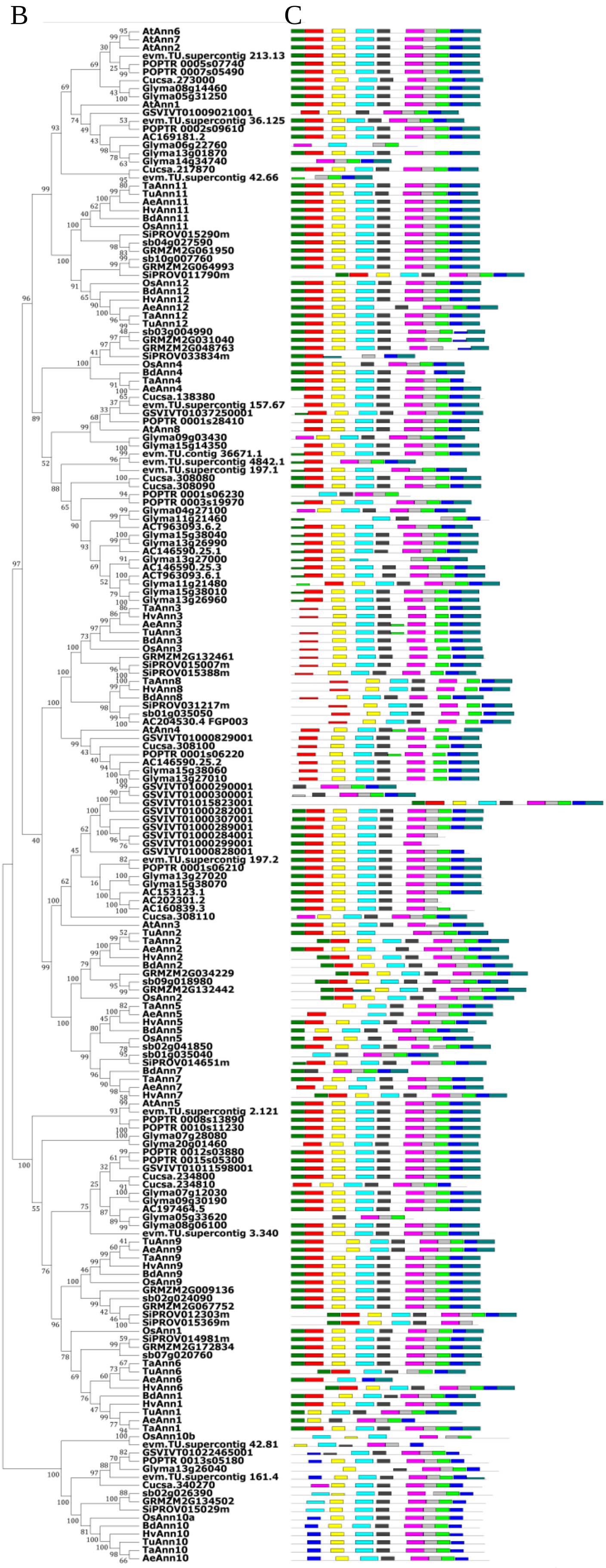
B
C
Motif 1
Motif 2
Motif 3
Motif 4
Motif 5
Motif 6
Motif 7
Motif 8
Motif 9
Motif 10

Additional file 5: Figure S2. Phylogenetic analysis and predicted structure of annexin proteins of 9 monocots plant and 7 discots plant. A. Ten Conserved sequence motifs identified in the amino acid sequences of 9 monocots plant (wheat, barley, *T.urartu*, *A.tauschii*, rice, maize, sorghum, *Brachypodium*, and *Setaria*)and 7 discots plant (*Vitis vinifera*, *Populus trichocarpa*, *Medicago truncatula*, *Cucumis Carica*, *papaya*, *Arabidopsis*, *Glycine_max*) as analyzed by MEME tools. The 1, 2, 3 and 4 motifs were found to be functionally associated with calcium-dependent phospho-lipid binding/calcium ion binding. B. Phylogenetic analysis of annexin protein sequences from 9 monocots plant and 7 discots plant. The tree was classified into five groups represented by roman letters (in black boxes) adjacent to the tree branches. C. The distribution of ten conserved motifs represented in the colored boxes was identified in the monocot and discots annexin protein sequences using the MEME 4.6.1 software. The order of the motif corresponds to their position in the individual protein sequences.
