# Supplementary material for: Comprehensive analyses of the annexin gene family in wheat
Source: BMC Genomics. 2016 May 28;17:415. doi: 10.1186/s12864-016-2750-y (PMC4884362; doi:10.1186/s12864-016-2750-y)
Supplement: Additional file 6: Table S4. — Orthologous groups of annexin genes in T.aestivuma, T.urartu, A.tauschii, H. vulgare, O.sativa and B.distachyon. (PDF 115 kb) [file 12864_2016_2750_MOESM6_ESM.pdf]

Additional file 6: Table S4. Orthologous groups of annexin genes in *T.aestivuma*, *T.urartu*, *A.tauschii*, *H. vulgare*, *O.sativa* and *B.distachyon*.

wheat	Homologues									
Gene	barley		*T.urartu*		*A.tauschii*		*B.distachyon*		*O.sativa*	
	Gene	Accession	Gene	Accession	Gene	Accession	Gene	Accession	Gene	Accession
TaAnn1	*HvAnn1*	MLOC_15770.2	*TuAnn1*	TRIUR3_22191	*AeAnn1*	AEGTA07604	*BdAnn1*	BRADI3G36240	*OsAnn1*	Os08g32970.1
TaAnn2	*HvAnn2*	MLOC_54933.3	*TuAnn2*	TRIUR3_11010	*AeAnn2*	AEGTA15612	*BdAnn2*	BRADI2G26770	*OsAnn2*	Os05g31760.1
TaAnn3	*HvAnn3*	MLOC_54932.1	*TuAnn3*	TRIUR3_11009	*AeAnn3*	AEGTA29052	*BdAnn3*	BRADI2G26760	*OsAnn3*	Os05g31750.1
TaAnn4	-	-	-	-	*AeAnn4*	AEGTA21287	*BdAnn4*	BRADI2G13620	*OsAnn4*	Os01g31270.1
TaAnn5	*HvAnn5*	MLOC_15543.3	-	-	*AeAnn5*	AEGTA42363	*BdAnn5*	BRADI1G18990	*OsAnn5*	Os07g46550.1
TaAnn6	*HvAnn6*	MLOC_55134.1	*TuAnn6*	TRIUR3_18622	*AeAnn6*	AEGTA16509	-	-	-	-
TaAnn7	*HvAnn7*	MLOC_3056.1	-	-	*AeAnn7*	AEGTA11977	*BdAnn7*	BRADI1G62120	-	-
TaAnn8	*HvAnn8*	AK370408	-	-	-	-	*BdAnn8*	BRADI1G62130	-	-
TaAnn9	*HvAnn9*	AK249691.1	*TuAnn9*	TRIUR3_32496	*AeAnn9*	AEGTA10144	*BdAnn9*	BRADI4G29680	*OsAnn9a*	Os09g23160.1
TaAnn10	*HvAnn10*	MLOC_54650.4	*TuAnn10*	TRIUR3_04012	*AeAnn10*	AEGTA28110	*BdAnn10*	BRADI4G31920	*OsAnn10*	Os09g27990.1<br>Os09g27990.2
TaAnn11	*HvAnn11*	MLOC_51591.2	*TuAnn11*	TRIUR3_16577	*AeAnn11*	AEGTA31729	*BdAnn11*	BRADI3G58830	*OsAnn11*	Os02g51750.1
TaAnn12	*HvAnn12*	AK248515	*TuAnn12*	TRIUR3_03306	*AeAnn12*	AEGTA27331	*BdAnn12*	BRADI1G45487	*OsAnn12*	Os06g11800.1

-: Not found
