# Supplementary material for: Comprehensive analyses of the annexin gene family in wheat
Source: BMC Genomics. 2016 May 28;17:415. doi: 10.1186/s12864-016-2750-y (PMC4884362; doi:10.1186/s12864-016-2750-y)
Supplement: Additional file 7: Table S5 — Orthologous groups of annexin genes in T.aestivuma, T.urartu, A.tauschii, H. vulgare, O.sativa and B.distachyon. (PDF 101 kb) [file 12864_2016_2750_MOESM7_ESM.pdf]

Additional file 7: Table S5. Orthologous groups of annexin genes in *T.aestivuma*, *T.urartu*, *A.tauschii*, *H. vulgare*, *O.sativa* and *B.distachyon*.

Orthologous Groups	Genes	Missing data
OrthoMCL1	*AeAnn1*, *BdAnn1*, *HvAnn1*, *TaAnn1*, *HvAnn6*, *TaAnn6*, *TuAnn6*	*OsAnn6*, *BdAnn6*
OrthoMCL 2	*AeAnn2*, *BdAnn2*, *HvAnn2*, *OsAnn2*, *TaAnn2*, *TuAnn2*	
OrthoMCL 3	*AeAnn3*, *BdAnn3*, *HvAnn3*, *OsAnn3*, *TaAnn3*, *TuAnn3*	
OrthoMCL 4	*AeAnn4*, *BdAnn4*, *OsAnn4*, *TaAnn4*	*TuAnn4*, *HvAnn4*
OrthoMCL 5	*AeAnn5*, *BdAnn5*, *HvAnn5*, *OsAnn5*, *TaAnn5*	*TuAnn5*,
OrthoMCL 6	*AeAnn7*, *HvAnn7*, *TaAnn7*	*TuAnn7*, *OsAnn7*
OrthoMCL 7	*BdAnn8*, *HvAnn8*, *TaAnn8*	*TuAnn8*, *AeAnn8*, *OsAnn8*
OrthoMCL 8	*AeAnn9*, *BdAnn9*, *HvAnn9*, *OsAnn9*, *TaAnn9*, *TuAnn9*	
OrthoMCL 9	*AeAnn10*, *BdAnn10*, *HvAnn10*, *OsAnn10*, *TaAnn10*, *TuAnn10*	
OrthoMCL 10	*AeAnn11*, *BdAnn11*, *HvAnn11*, *OsAnn11*, *TaAnn11*, *TuAnn11*, *AeAnn12*, *BdAnn12*, *HvAnn12*, *OsAnn12*, *TaAnn12*, *TuAnn12*	

The orthologs of *Ann1-12* genes among *T.aestivuma*, *T.urartu*, *A.tauschii*, *H. vulgare*, *O.sativa* and *B.distachyon* were identified by OrthoMCL (v1.4) software with the P value of 1e-20.
