# Supplementary material for: Comprehensive analyses of the annexin gene family in wheat
Source: BMC Genomics. 2016 May 28;17:415. doi: 10.1186/s12864-016-2750-y (PMC4884362; doi:10.1186/s12864-016-2750-y)
Supplement: Additional file 8: Figure S3. — Phylogenetic analysis of annexin protein sequences from three monocots (wheat, barely and rice) and two dicots (Arabidopsis thaliana and Glycine max). (PDF 65 kb) [file 12864_2016_2750_MOESM8_ESM.pdf]

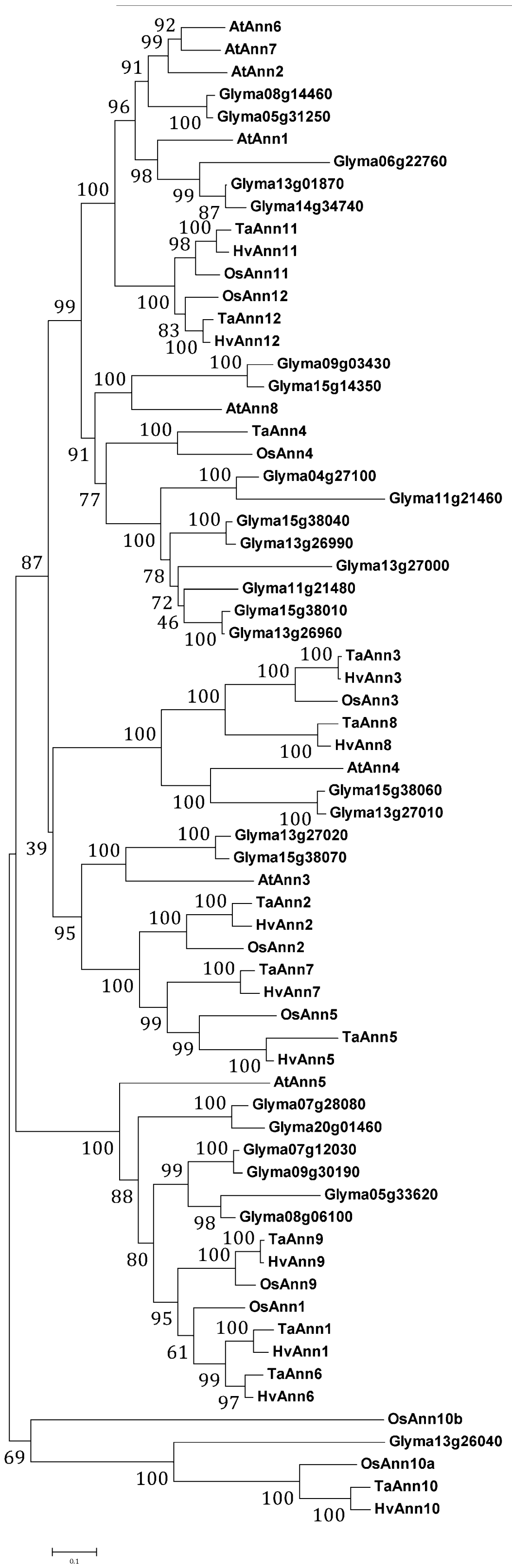

Additional file 8: Figure S3. Phylogenetic analysis of annexin protein sequences from three monocots (wheat, barely and rice) and two dicots (*Arabidopsis thaliana* and *Glycine max*)**.**
