# Supplementary material for: Comprehensive analyses of the annexin gene family in wheat
Source: BMC Genomics. 2016 May 28;17:415. doi: 10.1186/s12864-016-2750-y (PMC4884362; doi:10.1186/s12864-016-2750-y)
Supplement: Additional file 9: Figure S4. — Sequence aligment and logos of four annexin domains in the dicots and monocots. (PDF 1110 kb) [file 12864_2016_2750_MOESM9_ESM.pdf]

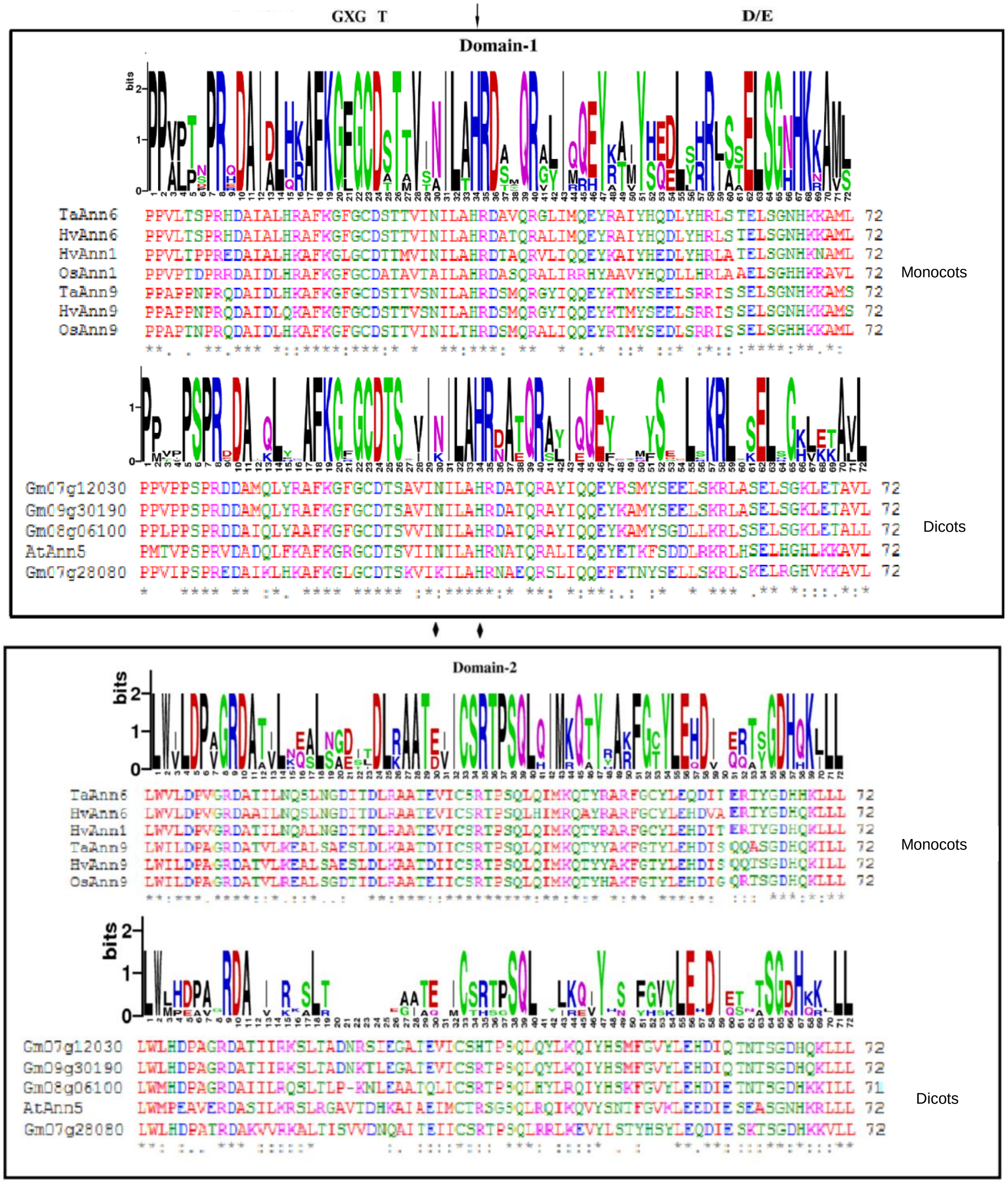
GXG T
D/E
Domain-1
bits
TaAnn6
HvAnn6
HvAnn1
OsAnn1
TaAnn9
HvAnn9
OsAnn9
Monocots
Gm07g12030
Gm09g30190
Gm08g06100
AtAnn5
Gm07g28080
Dicots
Domain-2
bits
TaAnn6
HvAnn6
HvAnn1
TaAnn9
HvAnn9
OsAnn9
Monocots
bits
Gm07g12030
Gm09g30190
Gm08g06100
AtAnn5
Gm07g28080
Dicots

I R I

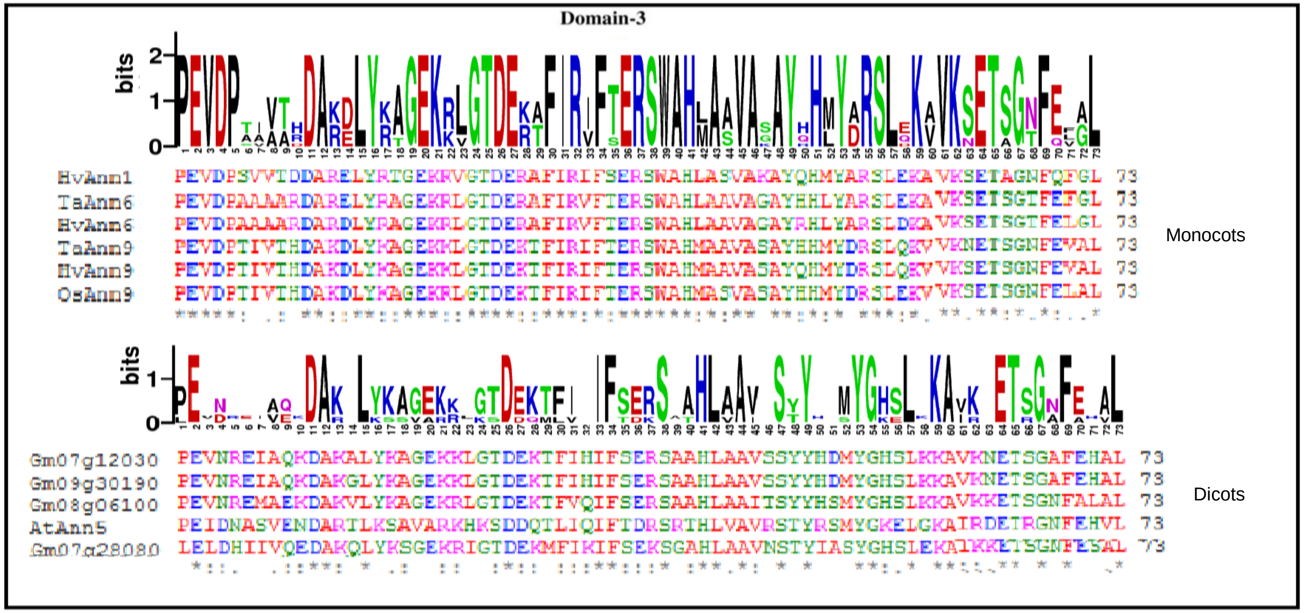

DXXG

GXG T

D/E

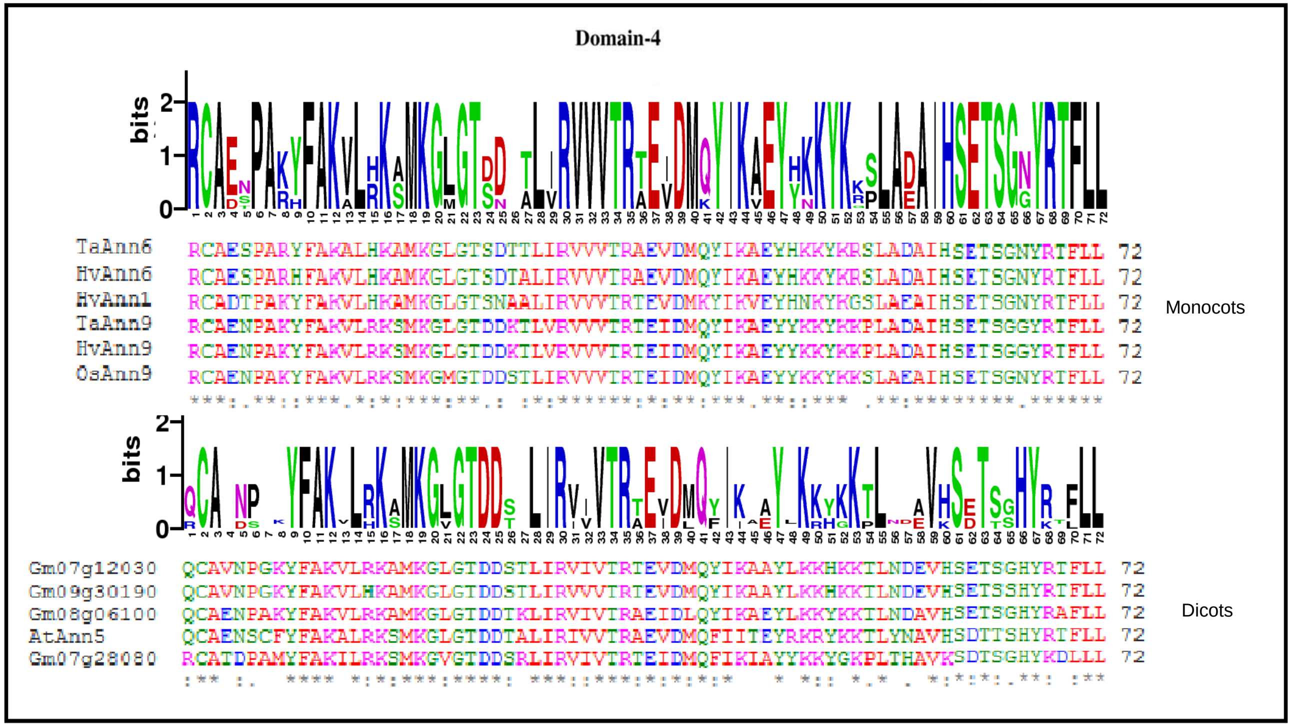

Additional file 9: Figure S4. Sequence aligment and logos of four annexin domains in the dicots and monocots. The four domain of the 11 plant annexins from IV subfamily of three monocots (wheat, barley and rice) and two dicots (*Arabidopsis* and *Glycine max*) were performed based on the structural alignment obtained through Clustal W, The sequence logos were generated by amino acid alignment of individual domains from 11 annexin gene in IV subfamily using WebLogo. The taxon-specific indels were removed to optimize the alignments. The height of letter designating the amino acid residue at each position represents the degree of conservation. The GXGT and D/E, IRI and DXXG motifs are represented on the top of each plot. The conserved His residue in the heme motif is indicated by an arrow . The residues thought to be involved in ion channel activity are represented as diamonds. Asterisks (*) indicated the conserved residues observed in the alignment of 11 annexins. The numbers on the x-axis represent the sequence positions in annexin domains. The y-axis represents the information content measured in bits.
