# Supplementary material for: Comprehensive analyses of the annexin gene family in wheat
Source: BMC Genomics. 2016 May 28;17:415. doi: 10.1186/s12864-016-2750-y (PMC4884362; doi:10.1186/s12864-016-2750-y)
Supplement: Additional file 10: Figure S5. — Gene Structure analysis of Arabidopsis, rice, barley and wheat annexin genes. (PDF 65 kb) [file 12864_2016_2750_MOESM10_ESM.pdf]

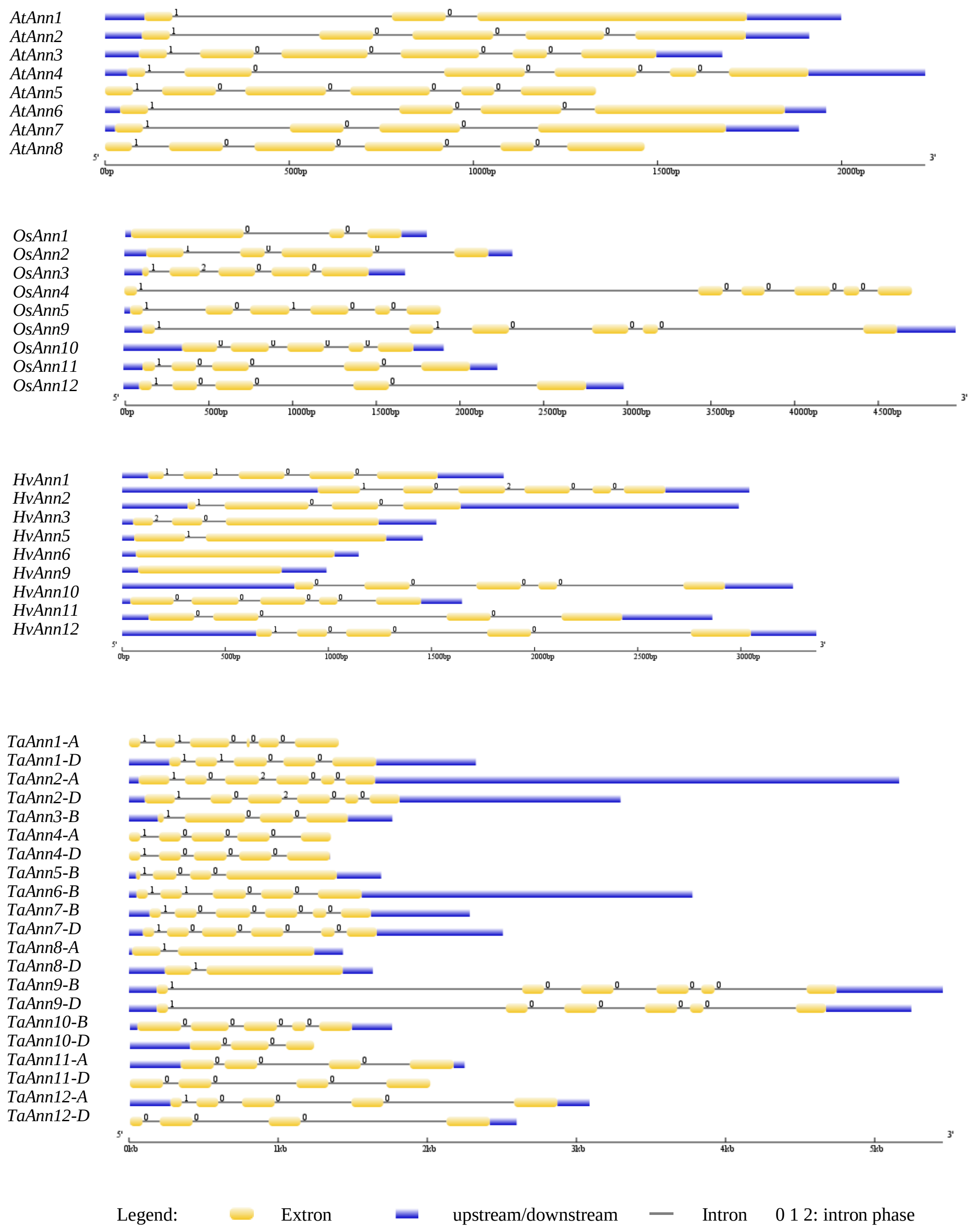

Additional file 10: Figure S5. Gene Structure analysis of Arabidopsis, rice, barley and wheat annexin genes. Gene structures were generated from GSDS (http://gsds.cbi.pku.edu.cn/chinese.php). Exons, UTRs, introns and intron phases are shown.
