# Supplementary material for: Comprehensive analyses of the annexin gene family in wheat
Source: BMC Genomics. 2016 May 28;17:415. doi: 10.1186/s12864-016-2750-y (PMC4884362; doi:10.1186/s12864-016-2750-y)
Supplement: Additional file 11: Table S6. — Putative cis-elements in the 2 kb upstream promoter region of translation start site in wheat annexin genes. (PDF 13 kb) [file 12864_2016_2750_MOESM11_ESM.pdf]

Additional file 11: Table S6. Putative cis-elements in the 2 kb upstream promoter region of translation start site in wheat annexin genes.

Gene name	ABRE	DRE/CRT	LTRE
TaAnn1-D	1	3	4
TaAnn2-A	4	0	0
TaAnn2-D	3	0	0
TaAnn3-B	3	0	2
TaAnn5-B	2	0	2
TaAnn6-B	0	1	1
TaAnn7-B	3	6	8
TaAnn7-D	1	2	1
TaAnn8-A	3	2	3
TaAnn8-D	2	2	1
TaAnn9-B	7	7	0
TaAnn10-B	5	5	3
TaAnn10-D	3	1	4
TaAnn11-A	1	1	3
TaAnn12-A	4	5	10
