# Supplementary material for: Comprehensive analyses of the annexin gene family in wheat
Source: BMC Genomics. 2016 May 28;17:415. doi: 10.1186/s12864-016-2750-y (PMC4884362; doi:10.1186/s12864-016-2750-y)
Supplement: Additional file 12: Figure S6. — Expression of TaAnn10 and TaCDPK15 in male sterile line BS366 under sterile and fertile condition. (PDF 129 kb) [file 12864_2016_2750_MOESM12_ESM.pdf]

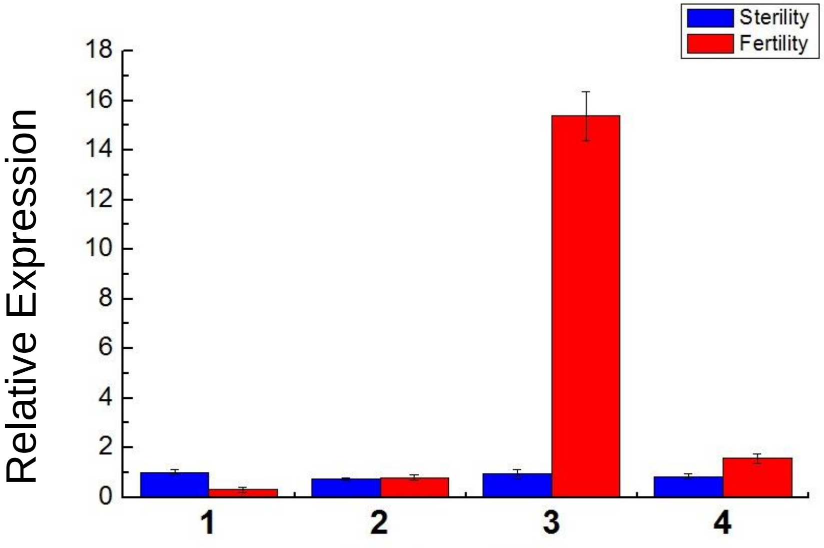

TaAnn10

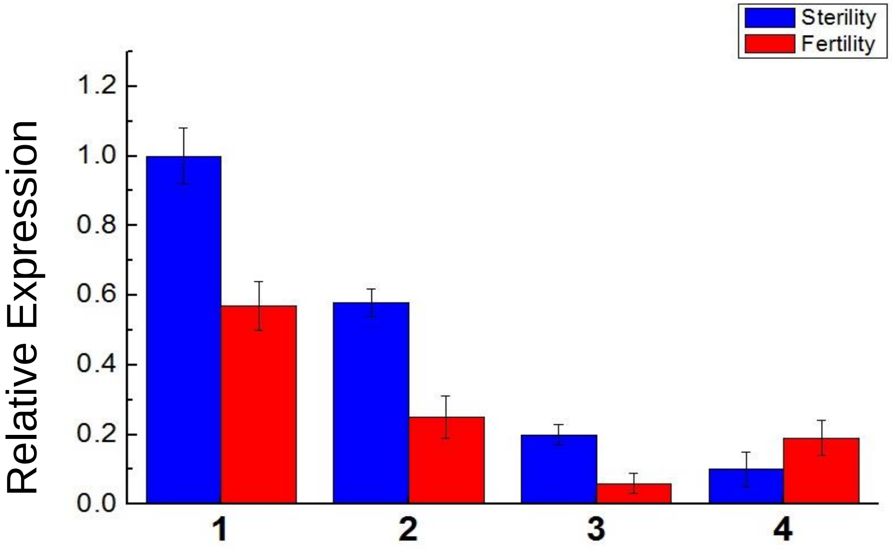

TaCDPK15

Additional file 12: Figure S6. Expression of *TaAnn10* and *TaCDPK15* in BS366 under sterile and fertile condition. A. *TaAnn10* down-regulated expression levels of Ca^{2+} sensors in male fertility induced by cold (Cold treatment, comprising 10 ℃ with a 12 h photoperiod). B. *TaCDPK15* Up-regulated expression in response to cold stress (Cold treatment, comprising 10 ℃ with a 12 h photoperiod) in spikes of the wheat TGMS line BS366 by RT-PCR. The expression level of *actin* was used as the internal control to standardize the RNA samples for each reaction. Error bars are the SDs of three technical replicates 1: Spike at stamen and pistil initiation stage; 2: anther at anther seperation stage; 3: anther at meiosis stage 3.0-mm anthers at the meiotic division stage (Tang et al., 2011); 4 anther at uninucleate stage. Black color: Low-temperature and short-photoperiod zone, sterile condition. Whiter color: High-temperature and long-photoperiod zone, fertile condition. RT-PCR primer (F: 5’ atcgacttcggcctctccatcttc 3’; R: 5’ caatcccgctcttcaactcttcat 3’) is from Li et al., 2008.
