# Supplementary material for: Comprehensive analyses of the annexin gene family in wheat
Source: BMC Genomics. 2016 May 28;17:415. doi: 10.1186/s12864-016-2750-y (PMC4884362; doi:10.1186/s12864-016-2750-y)
Supplement: Additional file 13: — Full set of annexin sequences from wheat (T. aestivum), barely (H.vulgare), T.urartu and A.tauschii. (PDF 208 kb) [file 12864_2016_2750_MOESM13_ESM.pdf]

Additional file 13: Full set of annexin sequences from wheat (*T. aestivum*), barely (*H.vulgare*), *T.urartu* and *A.tauschii*.

The sequences below are the complete collection of full length cDNA/mRNA sequences for annexin genes from barely (*H.vulgare*), *T.urartu* and *A.tauschii*. Sequences derived solely from GB ESTs may not be eligible for submission to GB as third party annotations. Sequences derived from Triticale hiseq2000 EST libraries which are identical to *T. aestivum* annexin are eligible for submission and this is in progress.

(1). Wheat (*T. aestivum*)

>TaAnn1-A
MASLSVPPVLTPPRDDAVALHKAFKGFGCDSTTVTNILAHRDSAQRALILHEYKAIYHQDLYHRLATELSG
NHKCSRNEPAWLWLQNAMLLWVLDPVGRDATILNQALNSDITDLRAATEVICSRTPSQLQIMKQTYRARF
GCYLEHDITERTYGDHQKLLLAYLGLYQAGEKRVGTDERAFIRIFSERSWAHMVSVANAYQHMYARSLEK
AVKSETTGNFQFGLLTILRCADTPAKYFAKVLHKAMKGLGTSNAALTRVAVTRTEVDMKYIKAEYHNKYK
GSLAEAIHSETSGNYRTFLLSLVGWDR

>TaAnn1-D
MASLSVPPVLTPPRDDAVALHKAFKGFGCDSTTVTNILAHRDSAQRALILHEYKAMYHQDLYHRLATELS
GNHKNAMLLWVLDPAGRDATILNQALNGDITDLRAATEVICSRTPSQLQIMKQTYRARFGCYLEHDITERT
YGDHQKILLAYLGVPRNEGPEVDPSAVTDDARELYRAGEKRVGTDERAFIRIFTESSRAHLVSIANAYQHM
YARSLEKTLKSETTGNFQFGLLTILRCADTPAKYFAKVLHKAMKGLGTSNAALIRVAVTRTEVDMKYIKAE
YHNKYKGSLAEAIHSETSGNYRTFLLSLVGRDR

>TaAnn2-A
MCGWCGCLECIHNIPPLNLLFLHFSSAARTGREGEEGARTLSPMASISVPDPAPSPTEDAESIRKAVQGWGT
DENALIEILGHRTAAQRAEIAVAYEGLYDKPLLRTLQDELSSHFKGAMTLWAMDPAARDAKLAYKALRKK
GGDRHAWVLIEVACASSPDHLVAVRKAYCSAYDSSLEEDVAACPLYKDPLKQFLVRLVSSYRHGGEHVDG
ELARAEAAELHGAVAAKKQPLHGDVVRIVSSRSKPQLKATFQHYKREHGKAIDEVLEGNRNDKLSAMLK
TAVWCLTSPEKHFAEVIRSSIIGLGTDEESLTRAIVSRAEVDLKKVKEEYKVRYKTTVTKDVVGDTSGYYQ
GILLTLIGAE

>TaAnn2-B
MCGWCCCLECIHNIPPLNLLFLHFSSAPRTGRDGEGARTLSPMASISVPDPASSPTEDAENIRKAVQGWGT
DENALIEILGHRTAAQRAEIAVAYEGLYDKPLLRTLQDELSSHFKGAMTLWTMDPAARDAKLAYKALRKK
GGDRHAWVLIEVACASSPDHLVAVRKAYCSAYDSSLEEDVAACPLYKEPLKQFLVRLVSSYRDGGDHVDG
ELARAEAAELHGAVAAKKQPLHGDVVRIVSSRSKPQLKATFQHYKREHGKAIDEVLEGNRNDKLSAMLK
TAVWCLTSPEKHFAEVIRSSIIGLGTDEESLTRAIVSRAEVDLKKVKEEYKVRYKTTVTKDVVGDTSGYYQ
GILLTLIGAE

>TaAnn3-A
MADEVQALTKAFSGLGGLGVDEPTMVAALANWRKQPEKRSGFRKSFPGLFKEHGVIERCEDEYMLHLA
AEFSRFKNLMVLWAMHPWERDARLAHHVLHQAHPAAILVEVACTRSAEELLGARKAYMALFHHSLEEDV
AYRAKDKPYCSLLVGLVSAYRYEGPKVSDDTAKAEAKALGAAVKSAAGGKLVENDEVVRILTTRSKPHLV
QTFKYYKELHGKHIQEDLGSEEALREAVQCLATPERYFSQVMAAALREGADHHGKEALARVAVTRSDVD
MDGIRAAYQEQFGAKLEDAVAGSAHGHFKDALVSLIVGK

>TaAnn3-B
MADEVQALTKAFSGLGGLGVDEPTMVAALANWRKQPEKRSGFRKSFPGLFKEHGVIERCEDEYMLHLA
AEFSRFKNLMVLWAMHPWERDARLAHHVLHQAHPSAIVVEIACTRSAEELLGARKAYMALFHHSLEEDV
AYRAKDKPYCSLLVGLVSAYRYEGPKVSDDTAKAEAKALGAAVKSAAGGKLVENDEVVRILTTRSKPHLV
QTFKYYKELHGKHIEEDLGSEEALRETVQCLATPERYFSQVMAAALREGADHHGKEALSRVAVTRSDVD
MDGIRAAYQEQFGAKLEDAVAGSAHGHFKDALVSLIVGK
>TaAnn4-A
MTTITVPQVVPSPVEDAEALMKAFQGWGTDEQAVIYILAHRDAAQRKLIRQAYEEKYNELLTDRLQSELS
GDLQTAMGYWVLDPTERQAVIANAATKCIDEEYPVIVEIACANSPAELLEVKQAYHAIYKRSLEEDVAAGA
TGNLRNLLVALVSTYRYDGDEVNGGLARSEAKIIQADVKNGVTADHGELIRVLGTRSRAQLGATFNCFRD
EHGTTVTKVLRNAMHKSGTDEESLLRVVVMHAEKDLRAIKDEFHRRASVALEQAIAKETSGDFKTFIMAL
VGTSQ
>TaAnn4-D
MATIKVPQVVPSPVEDAEALMKAFQGWGTDEQAVIYILAHRDAAQRKLIRLAYEEKYDER
LTDRLESELSGDLQTAMGYWVLDPTERQAVIANAATKCIDEEYPVIVEIACANSPAELLEVKRAYHAIYKR
SLEEDVAAGATGNLRNLLVALVSAYRYDGDEVNGGLARSEAKIIQADVKNGVTADHGELIRVLGTRSRAQ
LGATFNCFRDEHGTTVTKALLHAPDTTGYARALRTAARCIADTDKYFAKVLRNAMHKSGTDEESLLRVV
VMRAEKDLRGIKDEFHKRASVALEQAIAKE
ASGDFKTFITALVGT
>TaAnn5-B
MSAIVVPSPVPSPSDDAEGIRKALQGWRADKEALVRILAGPLLNSFRQRLSRQYCPVTVDFWKAIILWTM
DPAERDANLVHGALRRRGDGDHLAVLVEVSCASDPDHLVAVRRAYRSLFGCSVEDLASCAALQQPLRKM
LVSLVSSYRYGGDRVDADVAKLEASQLSEAVRKKQPHHDEVVQILGTRSKPQLRATLRRYREDHGTDIVE
DIDSRCSSQFARTLKSAVWCLTSPEKHFAEMIRESVVGLGTYEDMLTRVVVSRAEIDMKQIKEEYRARFKT
TVTCDVVDDTSFGYKDILLALVGCEEE
>TaAnn6-A
MASLSVPPVLTSPRHDAIALHRAFKGFGCDSTTVINILAHRDAVQRGLIMQEYRAIYHQDLYHRLSTELSG
NHKKAMLLWVLDPVGRDATILNQSLNGDITDLRAATEVICSRTPSQLQIMKQTYRARFGCYLEQDITERTY
GDHHKLLLAYLGIPRYEGPEVDPAAAARDARELYRAGEKRLGTDERAFIRVFTERSWAHLAAVAGAYHHL
YARSLEKAVKSETSGTFEFGLLTILRCAESPARYFAKALHKAMKGLGTSDTTLIRVVVTRAEVDMQYIKAE
YHKKYKRSLADAIHSETSGNYRTFLLSLVGRDR
>TaAnn6-A
MASLSVPPVLTPPRDDAVALHKAFKGFGCDSTTVTNILAHRDSAQRALILHEYKAMYHQDLYHRLATELS
GNHKNAMLLWVLDPAGRDATILNQALNGDITDLRAATEVICSRTPSQLQIMKQAYRARFGCYLEHDITERT
YGDHQKLLLAYLGVRRNEGPEVDPSAVTDDARELYQAGEKRVGTDERAFIRIFSERSWAHMVSVANAYQ
HMYARSLEKAVKSETTGNFQFGLLTILRCADTPAKYFAKVLHKAMKGLGTSNAALTRVAVTRTEVDMKYI
KAEYHNKYKGSLAEAIHSETSGNYRTFLLSLVGRDR
>TaAnn6-B
MASLSVPPVLTSPRHDAIALHRAFKGFGCDSTTVINILAHRDAVQRALIMQEYRAMYRQDLYHRLSTELSG
NHKKAMLLWVLDPVGRDATILNQSLNGDITDLRAATEVICSRTPSQLQIMKQAYRARFGCYLEQDITERTY
GDHQKLLLAYLGIPRYEGPEVDPAAAARDARELYRAGERRLGTDERAFIRVFTERSWAHLAAVAGAYHHL
YARSLEKAVKSETSGTFELGLLTILRCAESPARYFAKALHKAMKGLGTSDTTLIRVVVTRAEVDMQYIKAE
YHKKYKRSLADAIHSETSGNYRTFLLSLVGRDR
>TaAnn7-B
MATIAVPTPLPSPAADAESLRNAVQGWGTDEKALVEILGRRTAAQRAEIRRAYASLYKESLLTRLHDELSGH

FQKAMVLLATDPAERDAKLVREALGRRGDDRDAWVLIEAACAATPEHLVAVRRAYRSLHGSSLEEDVAA
CSAFQEPLRKLLVSLVRSYRCAEESVDMDVAKLEAAQLAEAVRRKKQPHGGEVVRIVSTRSKSQLAATFR
CYKEQHGSDIEEDMKQYSSSQFARMLKIAVWCLTSPEKHYAEVIRYSILGLGTDEDTLTRAIVSRADIDME
KIKQEYRVRFKTTVTDDVVGDTSRYYMDILLALVGKE
>TaAnn7-D
MATIDVPTPVPSPAADADSLRNAVQGWGTDEKALVEILGRRTAAQRAEIRRAYASLYKESLLTRLHGELSG
HFQKAMVLLATDPAERDAKLAREALGRRGDDRDAWVLIEAACAAAPDHLVAVRRAYRSLHGSSLEEDVA
ACSAFQEPLRKLLVSLVRSYRCAEESVDMDVAKLEAAQLAEAIRRKKQPHAGEVVRIVSTRSKPQLAATF
RCYKEQHGSDIEEDMKQYSSSQFARMLKIAVWCLTSPEKHFAEVIRYSILGLGTDEDALTRAIVSRADIDM
KKIKQEYRVRFKTTVTDDVVGDTSGYYMDILLALVGKE
>TaAnn8-A
MQQFLAAQPSSNGSTAAAAAAAPPSSPSPLRSRPPRREQQQQQSIVKEATMADEHQELTKAFSGMGGLGV
EETALVSALGRWRKQPEKRAQFRRGFPGFFAPAAAGAGAGAIERCSDDYVRHLKTEFARFKTLMVLWAM
HPWERDARWAHRALHKKHHPVAVLVELACTRAADELLGARRAYHALYHRSLEEDVAYRVKDAAANRLL
LGLVTAYRYEGPRVDEELAKEEAAALSGAGAKAAQSELVVRVLATRSKPQLRATFRLYRELHGKPLEEEFG
GEAPCLREAVRCLESPARYFGEVIDGAFKEGADKQAKAALTRVVVSRSDADMEEIKEAYLKQHGDKLVDA
VAKNTHGHYRDALLAMIGK
>TaAnn8-D
MQQFLAAQPSSNGSTAAAAAPPPSPSPLRSRPPRRDQQQQSIKEATMADEHQELTKAFSGMGGLGVEETA
LVSALGRWRKQPEKRAQFRRGFPGFFEPAAAGAIERCSDDYVRHLKTEFARFKTMVLWAMHPWERDAR
WAHRALHKKHHPASVLVELACTRAADELLGARRAYHALYHRSLEEDVAYRVKDAAANRLLLGLVTAYRY
EGPRVDEELAREEAAALSAGPGAGAKAQSQSELVVRVLATRSKPQLRATFRLYRELHGKPLEEEFGGDAP
CLREAVRCLESPAKYFGEVIDGAFKEGADKQAKAALTRVVVSRSDADMEEIKEAYLKQHGAKLVDAVAK
NTHGHYRDALLAMIGK
>TaAnn9-B
MASLTLPPAPPNPRQDAIDLHKAFKGFGCDSTTVSNILAHRDSMQRGYIQQEYKTMYSEELSRRISSELSG
NHKKAMSLWILDPAGRDATVLKEALSAESLDLKAATDIICSRTPSQLQIMKQTYYAKFGTYLEHDISQQAS
GDHQKILLAYVGIPRYEGPEVDPTIVTHDAKDLYKAGEKKLGTDEKTFIRIFTERSWAHMAAVASAYHHM
YDRSLQKVVKNETSGNFEVALLTILRCAENPAKYFAKVLRKSMKGLGTDDKTLVRVVVTRTEIDMQYIKA
EYYKKYKKPLADAIHSETSGGYRTFLLSLVGSH
>TaAnn9-D
MASLTLPPAPPNPRQDAIDLHKAFKGFGCDSTTVSNILSHRDSMQRGYIQQEYKTMYSEELSHRISSELSGN
HKKALSLWILDPAGRDATVLKEALSAESLDLKAATDIICSRTPSQLQIMKQTYYAKFGTYLEHDISQQASG
DHQKILLAYVGIPRYEGPEVDPTIVTHDAKDLYKAGEKKLGTDEKTFIRIFTERSWAHMAAVASAYRHMY
DRSLQKVVKNETSGNFEVALLTILRCAENPAKYFAKVLRKSMKGLGTDDKTLVRVVVTRTEIDMQYIKAE
YYKKYKKPLADAIHSETSGGYRTFLLSLVGSH
>TaAnn10-A
MAARSPAAAGFENECREIHGACDEPRRLSRLLAHRSASERQQIKATYRAMFGEDLAGRLHKTLTANQDNE
LCNLLYLWMLDLAERDAIMARDAIESGAAADYRALVEVFTRRKQDQLFFTKQAYLARFKKNLEQDMVT
DPSHPYQRLLVALATSHKSHHDEPSQHIAKCDARRLYDAKNGGGTGSVDEAAILEMFSKRSIPQLRLALCS
YKHIYGHDFTKALKKNLARDFEESLRVVVKCIYTPSKYYCKLLQRSMQRPEADKRLVTRAILGSDDVGIN
EIKLAFKSNFGKNLADFIHESLPQSDYREFLWLWQGGQCLVAS
>TaAnn10-B
MAARSPAATGFENECREIHGACDEPRRLSRLLARRSTSERQQIKATYRAMFGEDLAGRLHKTLTANQDNE
SQHGLPLQLCNLLYLWMLDLAERDAIMARDAIESGAAADYRALVEVFTRRKQDQLFFTKQAYLARFKKN

LEQDMVTDPSHPYQRLLIALATSHKSHHDEPSQHIAKCDARRLYDAKNGGGTGSVDEAAVLEMFSKRSIP
QLRLALCSYKHIYGHDFTKALKKNLARDFEESLRVVVKCIYTPSKYYCKLLQRSMQRPEADKRLVTRAIL
GSGDVGINEIKLAFKSNFGKNLADFIHESLPQSDYREFLWLWQGGQCPVAS
>TaAnn10-D
MAARSPAAAGFENECREIHGACDEPRRLSRLLAHRSASERQQIKATYRAMFGEDLAGRLHKTLTANQDNE
SQHGLPLQLCNLLYLWMLDLAERDAIMARDAIESGAAADYRALVEVFTRRKQDQLFFTKQAYLARFKKN
LEQDMVTDPSHPYQRLLVALATSHKSHHDEPSRHIAKCDARRLYDAKNGGGTGSVDEAAILEMFSKRSIP
QLRLALCSYKHIYGHDFTKALKKNLAGDFEESLRVVVKCIYTPSKYYCKLLQRSMQRPEADKRLVTRAIL
GSDDVGINEIKLAFKSNFGKNLADFIHESLPQSDYREFLWLWQGGQCLVAS
>TaAnn11-A
MATLSVPAAVPAVAEDCEQLRKAFAGWGTNERLIVSILAHRDAAQRRAIRRAYAEAYGEELLRAIGDEIHG
KFERAVIQWTLDPAERDAELANEEAKKWQPGGRALVEIACARTPAQLFAAKQAYHDRFKRSLEEDVAAH
VTGDFRKLLVPLVSAYRYDGPEVNTSLAHSEAKLLNGKINEKAYSDDEVVRILTTRSKAQLLATFNSYNDQ
FSHPITKDLKEDPKNEFLSTLRAIIRCFTCPDRYFEKVVRLALGGVGTDEDTLTRVITTRAEVDLKVIKEAY
QKRNSVPLEKAVSKETSRDYEDMMLALLGAEY
>TaAnn11-B
MATLSVPAAVPAVAEDCEQLRKAFAGWGTNERLIVSILAHRDAAQRRAIRRAYAEAYGEELLRAIGDEIHG
KFERAVAQWTLDPAERDAELAGEEAKKWQPGGRALVEIACARTPAQLFAARQAYHDRFKRSLEEDVAAH
VTGEFRKLLVPLVSAYRYDGPEVNTSLAHSEAKILNGKINEKAYSDDEIIRILTTRSKAQLLATFNSYNDQFS
HPITKDLKEDPKNEFLSTLRAIIRCFTCPDRYFEKIIRLALGGVGTDENTLTRVITTRAEVDLKVIKEAYQKR
NSVPLDKAVSKETSRDYEDMMLALLGAEY
>TaAnn11-D
MATLSVPAAVPAVAEDCEQLRKAFAGWGTNERLIVSILAHRDAAQRRAIRRAYAEAYGEELLRAIGDEIHG
KFERAVTQWTLDPAERDAELAGEEAKKWQPGGRALVEIACARTPAQLFAAKQAYHDRFKRSLEEDVAAH
VTGDFRKLLVPLVSAYRYDGPEVNTSLAHSEAKILNGKINEKAYSDDEIVRILTTRSKAQLLATFNSYNDQF
SHPITKDLKEDPKNEFLSTLRAIIRCFTCPDRYFEKVVRLALGGVGTDENTLTRVITTRAEVDLKVIKEAYQ
KRNSVPLEKAVSKETSRDYEDMMLALLGAEY
>TaAnn12-A
MATLKVPSNVPALADDCDNLRKAFQGWGTNEALIISILGHRDASQRRAIRKHYADTYGEELRSITDEISGD
FERAVILWTLDPAERDAVLANETARKWHPGNPVLVEIACARGSKQLFAVRQAYHRFKRSLEEDVASHVTG
DFRKLLVPLVSSHRYEGPEVNTRLAHSEAKLLHEKIEHKAYADDEVIRILTTRSKAQLLATFNNYNDTFGHP
ITKDLKADPKDEFLKTLRAVIRCFTCPDRYFEKVARVAIAGNGTDENSLTRVITTRAEVDLKLIKEAYQKRN
SVPLEKAVAGDTSGDYESMLLALLGKE
>TaAnn12-D
MATLKVPSNVPALADDCDNLRKAFQGWGTNEALIISILGHRDAAQRRAIRKHYADTYGEELLRSITDEISG
DFERAVILWTLDPAERDAVLANETAKKWHPGNPVLVEIACARGSKQLFAARQAYHDRFKRSLEEDVAAHV
TGDFRKLLVPLVSSHRYEGPEVNTRLAHSEAKLLHEKIEHKAYGDDEVIRILTTRSKAQLLATFNNYNDTF
GHPITKDLKADPKDEFLKTLRAVIRCFTCPDRYFEKVARLAIAGNGTDENSLTRVITTRAEVDLKLIKEAYQ
KRNSVPLEKAVAGDTSGDYESMLLALLGKE

>TaAnn1-A
ATGGCGAGCCTGAGCGTGCCTCCGGTGCTCACGCCTCCGCGTGATGACGCCGTCGCTCTCCACAAGGC
CTTCAAAGGGTTCGGCTGCGACAGCACGACGGTGACTAACATACTTGCTCACCGCGACTCGGCGCAG
CGCGCGCTCATCCTGCACGAGTACAAAGCCATATACCATCAGGATCTCTACCATCGCCTAGCAACTGAA
CTGAGTGGAAACCACAAGTGCTCCCGAAACGAACCTGCATGGCTGTGGTTGCAGAATGCTATGCTGCT

TTGGGTCCTCGACCCCGTGGGGCGTGACGCGACCATACTGAACCAGGCTCTCAACAGCGACATCACC
GACCTGAGAGCCGCCACGGAGGTAATCTGCTCCAGGACGCCGTCGCAGCTGCAGATCATGAAGCAAA
CCTACCGCGCGAGGTTCGGTTGCTACCTCGAGCATGACATCACCGAACGCACCTACGGTGATCATCAG
AAGCTTTTGCTTGCGTACCTGGGACTGTATCAAGCCGGCGAGAAGAGGGTGGGCACCGATGAGCGGG
CCTTCATCCGCATCTTCAGCGAACGCAGCTGGGCGCACATGGTGTCCGTGGCCAACGCTTACCAGCAC
ATGTACGCCCGGTCCCTGGAGAAGGCCGTGAAGAGTGAGACGACAGGGAACTTCCAGTTCGGGCTGC
TGACCATCCTCAGGTGCGCCGACACTCCGGCAAAGTACTTTGCCAAGGTCCTGCACAAGGCGATGAA
AGGCCTGGGCACCAGCAACGCGGCGCTCACACGGGTGGCGGTGACGAGGACCGAGGTTGACATGAA
GTACATCAAGGCAGAGTACCACAACAAGTACAAGGGGTCGCTGGCTGAAGCTATCCACTCCGAGACG
TCGGGGAACTACCGAACCTTCCTCCTCTCACTCGTCGGCTGGGACCGCTAA

>TaAnn1-D

ATGGCGAGCCTGAGCGTGCCTCCGGTGCTCACGCCCCCGCGCGATGACGCCGTCGCTCTCCACAAGG
CCTTCAAAGGTTTCGGCTGCGACAGCACGACGGTGACCAACATACTTGCTCACCGCGACTCGGCGCA
GCGCGCGCTCATCCTGCACGAGTACAAAGCCATGTACCATCAGGATCTCTACCATCGCCTAGCAACCG
AGCTGAGTGGAAACCACAAGAATGCTATGCTGCTTTGGGTCCTCGACCCTGCTGGGCGTGATGCGACC
ATACTGAACCAAGCTCTCAACGGCGACATCACCGACCTGAGAGCCGCCACCGAGGTTATCTGCTCCAG
GACGCCGTCGCAGCTGCAGATCATGAAGCAAACCTACCGTGCGAGGTTCGGTTGCTACCTCGAGCATG
ACATCACCGAACGCACCTATGGAGATCATCAGAAGATTTTGCTTGCGTACCTGGGAGTCCCACGCAAC
GAAGGCCCGGAAGTTGATCCTTCGGCGGTGACAGACGACGCGAGGGAGCTGTATAGGGCCGGCGAG
AAGAGGGTGGGCACCGATGAGCGGGCCTTCATCCGCATCTTCACCGAAAGCAGCAGGGCACACCTGG
TGTCCATCGCCAACGCTTACCAGCACATGTACGCCCGGTCCCTGGAGAAGACCCTGAAGAGTGAGAC
GACAGGGAACTTCCAATTCGGGCTGCTGACCATCCTCAGGTGCGCCGACACTCCGGCAAAGTACTTTG
CCAAGGTCCTGCACAAGGCGATGAAAGGCCTGGGCACCAGCAACGCGGCGCTCATAAGGGTGGCGG
TGACGAGGACCGAGGTCGACATGAAGTACATC
AAGGCAGAGTACCACAACAAGTACAAGGGGTCGCTGGCTGAAGCTATCCACTCCGAGACGTCAGGGA
ACTACCGAACCTTCCTCCTCTCGCTCGTCGGCCGGGACCGCTAA

>TaAnn2-A

ATGTGTGGCTGGTGCGGCTGCCTCGAGTGCATCCATAACATCCCTCCCCTCAATCTCCTCTTCCTCCAC
TTCTCCTCGGCTGCACGGACGGGAAGGGAGGGAGAAGAAGGAGCGCGAACGCTCAGCCCCATGGCC
TCCATCTCGGTCCCGGACCCCGCCCCTTCCCCGACCGAGGATGCAGAGAGCATCAGAAAAGCAGTGC
AAGGGTGGGGGACGGACGAGAACGCGCTGATCGAGATCCTGGGCCACCGGACGGCGGCGCAGCGCG
CGGAGATCGCCGTCGCCTACGAGGGCCTCTACGACAAGCCCCTCCTCCGCACGCTCCAGGACGAGCT
CTCCAGCCACTTCAAGGGCGCGATGACGCTGTGGGCGATGGACCCGGCGGCGCGGGACGCCAAGCTG
GCCTACAAGGCCCTCAGGAAGAAGGGCGGCGACCGGCACGCCTGGGTGCTCATCGAGGTCGCCTGCG
CGTCGTCGCCGGACCACCTCGTCGCCGTCAGGAAGGCCTACTGCTCCGCCTACGACTCGTCGCTCGAG
GAGGACGTCGCCGCCTGCCCGCTCTACAAGGACCCCCTCAAGCAGTTCTTGGTGCGCCTGGTGAGCT
CGTACCGCCACGGCGGCGAGCACGTCGACGGCGAGCTGGCGAGGGCCGAGGCGGCCGAGCTGCACG
GCGCGGTGGCGGCCAAGAAGCAGCCGCTGCACGGGGACGTGGTCCGCATCGTCAGCTCCAGGAGCA
AGCCGCAGCTCAAGGCGACGTTCCAGCACTACAAGCGAGAGCACGGCAAGGCCATCGACGAGGTTC
TCGAGGGCAATCGCAACGACAAGCTGTCGGCGATGCTGAAAACTGCGGTCTGGTGCCTGACATCGCC
GGAGAAGCACTTCGCAGAGGTGATCCGGAGCTCGATCATCGGGCTCGGCACCGACGAGGAATCCCTG
ACCAGAGCGATTGTCTCGCGCGCCGAGGTCGACTTGAAGAAAGTGAAGGAGGAATACAAGGTGAGGT
ACAAGACGACGGTGACCAAGGATGTCGTCGGAGACACCTCCGGGTACTACCAGGGCATCCTGCTCAC
CCTCATCGGGGCCGAGTAG

>TaAnn2-B

ATGTGTGGCTGGTGCTGCTGCCTCGAGTGCATCCATAACATCCCTCCCCTCAATCTCCTCTTCCTCCACT
TCTCCTCGGCTCCACGGACGGGAAGGGACGGAGAAGGAGCACGAACCCTCAGCCCCATGGCCTCCAT
CTCGGTCCCGGACCCTGCCTCTTCCCCAACCGAGGATGCAGAGAACATAAGGAAAGCAGTACAAGGG
TGGGGGACGGACGAGAACGCGCTGATCGAGATCCTGGGCCACCGGACGGCGGCGCAGCGCGCGGAG
ATCGCCGTGGCCTACGAGGGCCTCTACGACAAGCCCCTCCTCCGCACGCTCCAAGACGAGCTCTCCAG
CCACTTCAAGGGCGCGATGACGCTGTGGACGATGGACCCGGCGGCGCGGGACGCCAAGCTGGCCTAC
AAGGCGCTCAGGAAGAAGGGCGGCGACCGGCACGCCTGGGTGCTCATCGAGGTCGCCTGCGCGTCG
TCGCCGGACCACCTCGTCGCCGTCAGGAAGGCCTACTGCTCCGCCTATGACTCGTCGCTCGAGGAGGA
CGTCGCCGCCTGCCCGCTCTACAAGGAGCCCCTCAAGCAGTTCTTGGTGCGCCTGGTGAGCTCGTACC
GCGACGGTGGCGACCACGTCGACGGCGAGCTGGCGAGGGCGGAGGCGGCCGAGCTGCACGGCGCGG
TGGCGGCCAAGAAGCAGCCGCTGCACGGGGACGTGGTCCGCATCGTCAGCTCCAGGAGCAAGCCGC
AGCTCAAGGCGACGTTCCAGCACTACAAGCGAGAGCACGGCAAGGCCATCGACGAGGTTCTCGAGG
GGAATCGCAACGACAAGCTCTCGGCGATGCTGAAAACTGCGGTCTGGTGCCTGACATCGCCGGAGAA
GCACTTCGCAGAGGTGATCCGGAGCTCAATCATCGGGCTCGGCACCGACGAGGAATCCCTGACCAGA
GCGATCGTCTCGCGCGCCGAGGTCGACTTGAAGAAAGTCAAGGAGGAATACAAGGTGAGGTACAAG
ACGACGGTGACCAAGGATGTCGTCGGCGACACTTCCGGCTACTACCAGGGCATCCTGCTCACCCTCAT
CGGGGCCGAGTAG

>TaAnn3-A

ATGGCCGACGAAGTTCAGGCGCTCACCAAGGCCTTCTCAGGTCTGGGAGGTCTGGGCGTGGACGAGC
CGACGATGGTGGCGGCGCTGGCGAACTGGCGGAGGCAGCCGGAGAAGCGGTCCGGGTTCCGAAAGA
GCTTCCCGGGGCTGTTCAAGGAGCACGGCGTGATCGAGCGGTGCGAGGACGAGTACATGCTGCACCT
GGCCGCCGAGTTCTCCCGCTTCAAGAACCTCATGGTGCTGTGGGCGATGCACCCGTGGGAGCGCGAC
GCCCGCCTCGCGCACCACGTCCTCCACCAGGCCCACCCGGCCGCCATCGTCGTGGAGGTCGCCTGCA
CCCGCTCCGCCGAGGAGCTCCTCGGCGCGCGCAAGGCCTACATGGCGCTCTTCCACCACTCCCTCGAG
GAGGACGTCGCCTACCGCGCCAAGGACAAGCCATACTGCAGCCTGCTGGTGGGGCTGGTGAGCGCGT
ACCGGTACGAAGGGCCCAAGGTGAGCGACGACACGGCCAAGGCGGAGGCCAAGGCGCTGGGCGCG
GCGGTGAAGAGCGCCGCCGGCGGCAAGCTGGTGGAGAACGACGAGGTGGTCAGGATCCTCACCACC
AGGAGCAAGCCTCACCTCGTCCAGACCTTCAAGTACTACAAGGAGCTGCACGGCAAGCACATCGAGG
AGGATCTGGGAAGCGAGGAGGCTCTGAGGGAGACCGTGCAGTGCCTGGCGACGCCGGAGAGGTACT
TCAGCCAGGTGATGGCGGCGGCGCTGAGGGAGGGCGCGGACCACCACGGGAAGGAGGCCCTGTCGC
GGGTGGCGGTGACGCGGTCGGACGTGGACATGGACGGCATCAGGGCGGCCTACCAGGAGCAGTTCG
GGGCCAAGCTCGAGGACGCCGTCGCCGGCAGCGCGCACGGGCACTTCAAGGACGCGCTCGTCTCGCT
CATCGTCGGCAAGTAA

>TaAnn3-B

ATGGCCGACGAAGTTCAGGCGCTCACCAAGGCCTTCTCAGGTCTGGGAGGTCTGGGCGTGGACGAGC
CGACGATGGTGGCGGCGCTGGCGAACTGGCGGAAGCAGCCGGAGAAGCGGTCCGGGTTCCGGAAGA
GCTTCCCGGGGCTGTTCAAGGAGCACGGCGTGATCGAGCGGTGCGAGGACGAGTACATGCTGCACCT
GGCCGCCGAGTTCTCCCGCTTCAAGAACCTCATGGTGCTGTGGGCGATGCACCCGTGGGAGCGCGAC
GCCCGCCTCGCGCACCACGTCCTCCACCAGGCCCACCCGTCCGCCATCGTCGTGGAGATCGCCTGCAC
CCGCTCCGCCGAGGAGCTCCTCGGCGCGCGCAAGGCCTACATGGCGCTCTTCCACCACTCCCTCGAG
GAGGACGTCGCCTACCGCGCCAAGGACAAGCCCTACTGCAGCCTGCTGGTGGGGCTGGTGAGCGCGT
ACCGATACGAAGGGCCCAAGGTGAGCGACGACACGGCCAAGGCGGAGGCCAAGGCGCTGGGCGCGG
CGGTGAAGAGCGCCGCCGGCGGCAAGCTGGTGGAGAACGACGAGGTGGTCAGGATCCTCACCACCA
GGAGCAAGCCTCACCTCGTCCAGACCTTCAAGTACTACAAGGAGCTGCACGGCAAGCACATCGAGGA

GGATCTGGGAAGCGAGGAGGCTCTGAGGGAGACCGTGCAGTGCCTGGCGACGCCGGAGAGGTACTT
CAGCCAGGTGATGGCGGCGGCGCTGAGGGAGGGCGCAGACCACCACGGGAAGGAGGCCCTGTCGCG
GGTGGCGGTGACGCGGTCGGACGTGGACATGGACGGCATCAGGGCGGCCTACCAGGAGCAGTTCGG
GGCCAAGCTCGAGGACGCCGTCGCCGGCAGCGCGCACGGGCACTTCAAGGACGCGCTCGTCTCGCTC
ATCGTCGGCAAGTAA

>TaAnn4-A
ATGACCACGATCACAGTTCCTCAAGTTGTCCCTTCTCCGGTTGAAGATGCTGAGGCGCTCATGAAGGC
CTTCCAAGGATGGGGCACCGACGAGCAGGCAGTGATCTACATACTAGCTCACCGGGACGCGGCCCAG
AGGAAGCTGATCCGGCAGGCGTACGAAGAGAAGTACAACGAGCTCCTCACCGACCGGCTCCAGTCTG
AACTTTCCGGCGATCTTCAGACAGCGATGGGCTACTGGGTACTCGACCCCACGGAGAGGCAGGCCGT
GATCGCCAACGCCGCCACGAAGTGCATCGACGAGGAGTACCCCGTGATCGTGGAGATCGCGTGCGCC
AACTCGCCCGCCGAGCTCCTGGAAGTGAAGCAGGCCTACCACGCCATCTACAAGCGCTCGCTGGAGG
AAGACGTCGCCGCTGGTGCCACGGGAAACCTCCGTAACCTCTTGGTCGCCCTGGTGAGCACCTACAG
GTACGACGGCGACGAGGTGAACGGCGGGCTGGCGAGATCGGAGGCGAAGATCATTCAAGCGGACGT
GAAGAACGGCGTCACGGCGGACCACGGGGAGCTCATTAGGGTCCTGGGCACCAGGAGCAGAGCCCA
GCTCGGCGCCACGTTCAACTGCTTCAGAGATGAGCACGGCACCACCGTCACCAAGGTCCTGAGGAAC
GCGATGCACAAGTCGGGGACCGACGAGGAGAGCCTGCTCCGGGTGGTGGTGATGCACGCGGAGAAG
GACCTCAGGGCCATCAAAGACGAGTTCCACAGGAGGGCGTCGGTTGCCTTGGAGCAAGCCATCGCCA
AGGAAACCTCCGGTGACTTCAAGACCTTCATCATGGCTCTTGTTGGGACTAGCCAATAA
>TaAnn4-D
ATGGCCACGATCAAAGTTCCTCAAGTTGTCCCTTCTCCGGTTGAAGATGCTGAGGCGCTCATGAAGGC
CTTCCAAGGATGGGGCACTGACGAGCAGGCAGTGATCTACATACTAGCTCACCGGGACGCGGCGCAG
AGGAAGCTGATCCGGCTGGCGTACGAAGAGAAATACGACGAGCGCCTCACCGACCGGCTCGAGTCAG
AGCTCTCCGGCGATCTTCAGACGGCGATGGGCTACTGGGTACTCGACCCCACGGAGAGGCAGGCCGT
GATCGCCAACGCCGCCACGAAATGCATCGACGAGGAGTACCCCGTGATCGTGGAGATCGCCTGCGCC
AACTCGCCCGCCGAGCTCCTGGAAGTGAAGCGGGCCTACCACGCCATCTACAAGCGCTCGCTGGAGG
AAGACGTCGCCGCTGGCGCCACGGGAAACCTCCGCAATCTCTTGGTCGCTCTGGTGAGCGCCTACAG
GTACGACGGCGATGAGGTGAACGGCGGGCTAGCGAGGTCGGAGGCGAAGATCATTCAAGCGGACGT
GAAAAACGGCGTCACGGCGGATCACGGGGAGCTCATCAGGGTCCTAGGCACCAGGAGCAGAGCCCA
GCTCGGCGCCACGTTCAACTGCTTCAGAGATGAGCACGGCACCACCGTCACCAAGGCCCTGCTGCAT
GCCCCCGACACGACGGGCTACGCGCGTGCGCTGCGGACCGCGGCGAGGTGCATCGCCGACACAGAC
AAGTACTTCGCCAAGGTCCTGAGGAACGCGATGCACAAGTCGGGGACCGACGAGGAGAGCCTGCTCC
GGGTGGTGGTGATGCGCGCGGAGAAGGACCTCAGGGGCATCAAAGACGAGTTCCACAAGAGGGCGT
CGGTTGCCCTGGAGCAAGCCATCGCCAAGGAAGCCTCCGGTGACTTCAAGACCTTCATCACGGCTCTT
GTTGGGACT
>TaAnn5-B
ATGTCCGCCATCGTCGTCCCCAGCCCCGTGCCGTCACCCTCCGACGACGCCGAGGGCATCAGGAAGG
CGCTCCAAGGATGGCGGGCGGACAAGGAGGCCCTGGTGCGCATACTGGCGGGGCCGCTCCTCAACTC
CTTCCGCCAGCGGCTCTCCCGCCAGTACTGCCCCGTCACCGTCGACTTCTGGAAGGCGATCATCCTGT
GGACGATGGACCCGGCGGAGCGGGACGCGAACCTGGTGCACGGCGCGCTGAGGCGGCGAGGGGACG
GCGACCACCTCGCCGTGCTGGTGGAGGTCTCGTGCGCGTCCGACCCCGACCACCTCGTGGCCGTCCG
GCGTGCCTACCGCTCCCTCTTCGGCTGCTCCGTCGAGGAGGACCTCGCGTCCTGCGCCGCGCTCCAGC
AGCCGCTCAGGAAGATGCTGGTGAGCCTCGTGAGCTCCTACCGCTACGGCGGTGACCGCGTCGACGC
GGACGTGGCGAAGCTGGAGGCGTCGCAGCTCTCGGAGGCCGTCCGGAAGAAGCAGCCGCACCACGA

CGAGGTGGTGCAGATCCTCGGCACCCGGAGCAAGCCCCAGCTCAGGGCCACGCTCCGGCGGTACAGG
GAGGATCACGGCACGGACATCGTCGAGGACATCGACAGCCGCTGCAGCAGCCAGTTCGCCAGGACGC
TGAAGAGCGCAGTCTGGTGCCTGACCTCGCCGGAGAAGCACTTCGCGGAGATGATCAGGGAGTCGGT
GGTGGGGCTGGGGACCTACGAGGACATGCTGACGAGGGTGGTCGTGTCGCGGGCGGAGATCGACATG
AAGCAGATCAAGGAGGAGTACAGGGCCAGGTTCAAGACCACCGTGACCTGCGACGTCGTCGACGAC
ACCTCTTTCGGCTACAAAGACATCCTGCTGGCCTTGGTTGGCTGCGAGGAAGAGTGA
>TaAnn6-A
ATGGCGAGCCTGAGCGTGCCTCCGGTGCTCACGTCCCCGCGCCATGACGCCATCGCCCTCCACAGGGC
CTTCAAAGGTTTTGGGTGCGACAGCACGACGGTGATCAACATCCTGGCGCACCGCGACGCGGTGCAG
CGCGGGCTCATCATGCAGGAGTACAGGGCCATATACCACCAGGACCTCTACCATCGCCTATCGACCGA
GCTCAGCGGGAACCACAAGAAGGCGATGCTGCTGTGGGTGCTGGACCCCGTGGGCCGCGACGCCAC
GATACTGAACCAGTCCCTCAACGGCGACATCACCGACCTGCGCGCCGCCACGGAGGTCATCTGCTCCC
GGACGCCGTCGCAGCTGCAGATCATGAAGCAGACCTACCGCGCCCGCTTCGGCTGCTACCTCGAGCA
GGACATCACCGAGCGCACCTACGGCGACCACCACAAGCTTCTGCTGGCGTACTTGGGGATCCCGCGGT
ACGAGGGCCCGGAGGTGGACCCGGCGGCGGCGGCGCGCGACGCGAGGGAGCTGTACCGCGCCGGCG
AGAAGCGGCTCGGCACCGACGAGCGCGCCTTCATCCGCGTCTTCACCGAGCGCAGCTGGGCGCACCT
CGCCGCCGTCGCCGGCGCCTACCACCACCTGTACGCGCGCTCCCTGGAGAAGGCCGTGAAGAGCGAG
ACGTCGGGGACCTTCGAGTTCGGGCTGCTGACGATCCTCCGGTGCGCCGAGAGCCCGGCGAGGTACT
TCGCCAAGGCGCTGCACAAGGCGATGAAGGGGCTGGGCACCAGCGACACGACGCTGATACGGGTGG
TGGTCACCAGGGCGGAGGTGGACATGCAGTACATCAAGGCCGAGTACCACAAGAAGTACAAGCGCTC
GCTCGCCGACGCCATCCACTCCGAGACCTCCGGCAACTACCGCACCTTCCTCCTCTCCCTCGTCGGCC
GCGACCGCTAA
>TaAnn6-B
ATGGCGAGCCTCAGCGTGCCCCCGGTGCTCACGTCCCCGCGCCACGACGCCATCGCACTCCACAGGG
CCTTCAAAGGTTTCGGGTGCGACAGCACGACGGTGATCAACATCCTGGCGCACCGGGACGCGGTGCA
GCGCGCGCTCATCATGCAGGAGTACCGGGCCATGTACCGGCAGGACCTCTACCACCGGCTGTCCACCG
AGCTCAGCGGGAACCACAAGAAGGCGATGCTGCTGTGGGTGCTGGACCCCGTGGGCCGCGACGCGA
CGATACTGAACCAGTCCCTCAACGGCGACATCACCGACCTGCGAGCCGCCACGGAGGTGATCTGCTC
CCGGACGCCGTCGCAGCTGCAGATCATGAAGCAGGCCTACCGCGCCCGCTTCGGCTGCTACCTCGAGC
AGGACATCACCGAGCGCACCTACGGCGACCACCAGAAGCTTCTGCTGGCGTACCTGGGGATCCCGCG
GTACGAGGGCCCGGAGGTGGACCCGGCGGCGGCGGCGCGCGACGCAAGGGAGCTGTACCGCGCCGG
CGAGAGGCGGCTGGGCACCGACGAGCGCGCCTTCATCCGCGTCTTCACCGAGCGCAGCTGGGCGCAC
CTCGCCGCCGTCGCCGGCGCCTACCACCACCTGTACGCGCGCTCTTTGGAAAAGGCCGTGAAGAGCG
AGACGTCGGGGACTTTCGAGTTGGGGCTGCTGACGATCCTCCGGTGCGCCGAGAGCCCGGCGAGGTA
CTTCGCCAAGGCGCTGCACAAGGCGATGAAGGGGCTGGGCACCAGCGACACGACGCTGATACGGGT
GGTGGTCACCAGGGCGGAGGTGGACATGCAGTACATCAAGGCCGAGTACCACAAGAAGTACAAGCG
CTCGCTCGCCGACGCCATCCACTCCGAGACCTCCGGCAACTACCGCACCTTCCTCCTCTCCCTCGTCG
GCCGCGACCGCTAA
>TaAnn7-B
ATGGCGACCATCGCCGTCCCGACGCCCCTGCCGTCCCCGGCCGCCGACGCGGAGAGCCTCAGGAATG
CCGTGCAAGGCTGGGGCACGGACGAGAAGGCCCTGGTGGAGATCCTCGGCCGGCGAACCGCCGCGC
AGCGCGCCGAGATCCGCCGGGCCTACGCCAGCCTCTACAAGGAGTCCCTCCTCACCCGCCTCCACGA
CGAGCTCTCCGGCCACTTCCAGAAAGCGATGGTGCTGCTGGCGACGGACCCGGCGGAGAGGGACGC
CAAGCTGGTGAGGGAGGCCCTGGGCAGGCGGGGCGACGACAGGGACGCGTGGGTGCTCATCGAGGC
CGCATGCGCCGCCACGCCGGAACACCTCGTCGCCGTCCGCCGCGCCTACCGGTCCCTCCATGGATCCT

CCCTGGAGGAGGACGTCGCCGCCTGCTCCGCGTTCCAAGAACCGCTCAGGAAGCTGCTGGTGAGCCT
GGTGAGGTCGTACCGGTGCGCGGAGGAGAGCGTGGACATGGACGTGGCCAAGCTGGAGGCCGCGCA
GCTGGCGGAGGCCGTCAGGAGGAAGAA
GCAGCCGCACGGCGGCGAGGTCGTCCGGATCGTGAGCACCAGGAGCAAGTCGCAACTCGCAGCCAC
GTTCCGGTGCTACAAGGAACAACATGGCAGCGACATCGAAGAGGACATGAAGCAGTACAGCAGCAGC
CAGTTTGCAAGGATGCTCAAGATTGCTGTCTGGTGCCTCACATCTCCTGAAAAGCACTATGCAGAGGT
TATCAGGTACTCCATCTTAGGACTTGGAACGGACGAGGACACACTCACCAGGGCAATCGTGTCCAGGG
CCGACATCGACATGGAGAAGATCAAGCAAGAGTACAGGGTCAGATTCAAGACGACCGTAACAGACGA
CGTCGTCGGTGATACCTCGCGGTATTACATGGATATCTTGTTGGCCTTGGTAGGGAAGGAGTGA
>TaAnn7-D
ATGGCGACCATCGACGTCCCGACGCCCGTGCCGTCCCCGGCCGCCGACGCGGATAGCCTCAGGAATG
CCGTGCAAGGCTGGGGCACGGACGAGAAGGCGCTGGTGGAGATCCTCGGCCGGCGAACCGCCGCGC
AGCGCGCCGAGATCCGCCGGGCCTACGCGAGCCTCTACAAGGAGTCCCTCCTCACCCGCCTCCACGG
CGAGCTCTCCGGCCACTTCCAGAAAGCGATGGTGCTGCTGGCGACGGACCCGGCGGAGAGGGACGC
CAAGCTGGCGAGGGAGGCCCTGGGCAGGCGGGGCGACGACAGGGACGCGTGGGTGCTCATCGAGGC
CGCCTGCGCCGCCGCGCCGGACCACCTCGTCGCCGTCCGCCGCGCGTACCGGTCCCTCCATGGATCCT
CGCTGGAGGAGGACGTCGCCGCCTGCTCCGCGTTCCAAGAACCGCTCAGGAAGCTGCTGGTGAGCCT
GGTGAGGTCGTACCGGTGCGCGGAGGAGAGCGTGGACATGGACGTGGCGAAGCTGGAGGCCGCGCA
GCTGGCGGAGGCCATCCGGAGGAAGAA
GCAGCCGCACGCCGGCGAGGTCGTCCGGATCGTGAGCACAAGGAGCAAGCCGCAGCTCGCCGCCAC
GTTCCGGTGCTACAAGGAACAACATGGCAGCGACATCGAAGAGGACATGAAGCAGTACAGCAGCAGC
CAGTTTGCAAGGATGCTCAAGATTGCTGTCTGGTGCCTCACATCTCCTGAAAAGCACTTCGCAGAGGT
TATCAGGTACTCCATCTTAGGGCTTGGAACGGACGAGGACGCACTCACCAGGGCAATCGTGTCCAGGG
CCGACATCGACATGAAAAAGATCAAGCAAGAGTACAGGGTCAGATTCAAGACGACCGTAACAGACGA
TGTCGTCGGCGATACCTCGGGGTATTACATGGATATCTTGTTGGCCTTAGTAGGGAAGGAGTGA
>TaAnn8-A
ATGCAGCAGTTTCTTGCAGCACAGCCGAGCAGTAACGGCAGCACTGCAGCAGCAGCAGCAGCAGCTC
CACCGTCGTCGCCGTCGCCCCTGCGTTCGAGGCCACCCAGGAGAGAGCAGCAGCAGCAGCAGAGCAT
CGTCAAGGAGGCCACCATGGCCGACGAGCACCAGGAACTCACCAAGGCCTTCTCAGGGATGGGAGG
TCTGGGCGTGGAGGAGACGGCGCTGGTGTCGGCGCTGGGGCGGTGGAGGAAGCAGCCGGAGAAGCG
GGCGCAGTTCCGCAGGGGCTTCCCGGGCTTCTTCGCGCCGGCGGCGGCGGGGGCGGGGGCGGGGGC
GATCGAGCGCTGCTCCGACGACTACGTGCGGCACCTCAAGACGGAGTTCGCCCGGTTCAAGACCCTC
ATGGTGCTGTGGGCGATGCACCCGTGGGAGCGCGACGCGCGCTGGGCGCACCGCGCGCTGCACAAGA
AGCACCACCCGGTGGCCGTCCTCGTCGAGCTCGCCTGCACGCGCGCCGCCGACGAGCTCCTCGGCGC
CCGCCGCGCCTACCACGCGCTCTACCA
CCGCTCCCTCGAGGAGGACGTCGCGTACCGCGTCAAGGACGCCGCCGCCAACCGCCTGCTGCTCGGG
CTCGTTACCGCCTACCGCTACGAGGGCCCGCGCGTCGACGAGGAGCTCGCCAAGGAGGAGGCCGCGG
CGCTCTCCGGCGCCGGAGCCAAGGCGGCCCAGAGCGAGCTCGTGGTGCGGGTGCTCGCCACCAGGA
GCAAGCCGCAGCTCCGGGCGACCTTCAGGCTGTACAGGGAGCTCCACGGCAAGCCGCTGGAGGAGG
AGTTCGGCGGCGAGGCGCCGTGCCTGCGGGAGGCCGTGAGGTGCCTCGAGTCGCCGGCCAGGTACTT
CGGCGAGGTGATCGACGGGGCGTTCAAGGAGGGGGCGGACAAGCAGGCCAAGGCGGCGCTGACCCG
CGTCGTCGTGTCCCGGTCCGACGCCGACATGGAGGAGATCAAGGAGGCCTACCTGAAACAGCACGGG
GACAAGCTCGTGGACGCCGTCGCCAAGAACACCCACGGACATTACAGGGACGCGCTGCTCGCCATGA
TCGGGAAGTGA
>TaAnn8-D

ATGCAGCAGTTTCTTGCAGCACAGCCGAGCAGTAACGGCAGCACTGCAGCAGCAGCAGCTCCACCGC
CGTCGCCGTCGCCCCTGCGTTCGAGGCCACCCAGGAGAGACCAGCAGCAGCAGAGCATCAAGGAGG
CGACCATGGCCGACGAGCACCAGGAGCTCACCAAGGCCTTCTCAGGGATGGGCGGCCTGGGCGTGGA
GGAGACGGCGTTGGTGTCGGCGCTGGGGCGGTGGAGGAAGCAGCCGGAGAAGCGGGCGCAGTTCCG
GAGGGGCTTCCCGGGATTCTTCGAGCCGGCGGCGGCGGGGGCGATCGAGCGCTGCTCCGACGACTAC
GTGCGCCACCTCAAGACGGAGTTCGCCCGGTTCAAGACCCTCATGGTGCTGTGGGCGATGCACCCGT
GGGAGCGCGACGCGCGCTGGGCGCACCGCGCGCTGCACAAGAAGCACCACCCGGCGTCCGTCCTCG
TCGAGCTCGCCTGCACGCGCGCCGCCGACGAGCTCCTCGGCGCCCGCCGCGCCTACCACGCGCTCTA
CCACCGCTCCCTCGAGGAGGACGTGGCGTACCGCGTCAAGGACGCCGCCGCCAACCGCCTGCTGCTC
GGGCTCGTCACCGCCTACCGCTACGAGGGCCCGCGCGTCGACGAGGAGCTCGCCAGGGAGGAGGCC
GCGGCGCTCTCTGCCGGCCCAGGCGCCGGCGCCAAGGCCCAGAGTCAGAGCGAGCTCGTGGTGCGG
GTGCTCGCCACCAGGAGCAAGCCGCAGCTCCGGGCGACCTTCCGGTTGTACAGAGAGCTCCACGGCA
AGCCGCTGGAGGAGGAGTTCGGCGGCGACGCGCCGTGCCTGCGGGAGGCGGTGAGGTGCCTCGAGT
CGCCGGCCAAGTACTTCGGCGAGGTGATCGACGGGGCGTTCAAGGAGGGGGCGGACAAGCAGGCCA
AGGCCGCCTTGACCCGCGTCGTCGTGTCCCGGTCCGACGCCGACATGGAGGAGATCAAGGAGGCCTA
CCTGAAACAGCACGGGGCCAAGCTCGTGGACGCCGTCGCCAAGAACACCCACGGACATTACAGGGA
CGCGCTGCTCGCCATGATCGGAAAGTGA
>TaAnn9-B
ATGGCGAGCCTCACCTTGCCGCCGGCGCCCCCGAACCCCCGCCAGGACGCCATCGACCTCCACAAGG
CCTTCAAAGGGTTTGGCTGTGATAGTACAACAGTTTCAAATATACTTGCTCACCGTGACTCAATGCAAC
GTGGATACATTCAACAGGAATACAAAACTATGTATTCTGAGGAACTTTCACGTCGTATATCGTCTGAAC
TCAGTGGAAACCACAAGAAAGCAATGTCGCTCTGGATTCTTGATCCTGCTGGACGTGATGCGACTGTT
CTGAAAGAAGCTCTAAGTGCTGAAAGTCTTGATCTGAAAGCAGCTACTGATATAATATGTTCCAGGAC
ACCATCACAGCTGCAAATAATGAAACAGACATATTATGCAAAGTTTGGTACTTATCTTGAGCATGACATT
AGTCAGCAAGCATCCGGCGATCATCAGAAGATCTTACTAGCCTATGTTGGCATTCCACGCTACGAAGGC
CCGGAGGTTGATCCCACTATAGTGACACATGATGCGAAGGACCTCTACAAAGCTGGTGAGAAAAAGC
TGGGCACAGATGAGAAAACCTTCATCCGCATCTTCACTGAACGCAGCTGGGCACACATGGCAGCTGTT
GCTTCTGCTTACCATCACATGTATGATCGGTCGTTACAGAAGGTTGTGAAGAATGAAACATCTGGAAAC
TTTGAAGTTGCTCTGTTAACTATCCTCAGATGTGCTGAGAATCCAGCGAAGTATTTTGCTAAGGTGTTG
AGGAAGTCCATGAAAGGTCTAGGTACTGATGACAAGACACTTGTAAGGGTTGTGGTAACAAGGACTG
AGATTGATATGCAGTATATCAAGGCAGAATATTACAAGAAATACAAAAAGCCTTTAGCTGATGCTATCCA
TTCCGAAACATCAGGCGGTTATCGGACATTCCTGCTTTCTCTTGTTGGTAGCCATTAG
>TaAnn9-D
ATGGCGAGCCTCACCTTGCCGCCGGCGCCCCCGAACCCGCGCCAGGACGCCATCGACCTCCACAAGG
CCTTCAAAGGGTTTGGCTGTGATAGTACAACAGTTTCAAATATACTTTCTCACCGTGACTCAATGCAAC
GTGGATACATTCAACAGGAATACAAAACTATGTATTCTGAGGAACTTTCACATCGTATATCGTCTGAACT
CAGTGGAAACCACAAGAAAGCACTGTCGCTCTGGATTCTTGATCCTGCTGGACGTGATGCGACTGTTC
TGAAAGAAGCTCTAAGTGCTGAAAGTCTTGATCTGAAAGCAGCTACTGATATAATTTGTTCCAGGACA
CCATCACAGCTGCAAATAATGAAACAGACATATTATGCAAAGTTTGGTACTTATCTTGAGCATGACATTA
GTCAGCAAGCATCCGGCGATCATCAGAAGATCTTACTAGCCTATGTTGGCATTCCACGCTACGAAGGCC
CGGAGGTTGATCCCACTATAGTGACACATGATGCGAAGGACCTCTACAAAGCTGGTGAGAAAAAGCT
GGGCACAGATGAGAAAACCTTCATCCGCATCTTCACTGAGCGCAGCTGGGCACACATGGCAGCTGTT
GCTTCTGCTTACCGTCACATGTATGATCGGTCGTTACAGAAGGTTGTGAAGAATGAAACATCTGGAAA
CTTTGAAGTTGCTCTGTTAACTATCCTCAGATGTGCTGAGAATCCAGCGAAGTATTTTGCTAAGGTGTT
GAGGAAGTCCATGAAAGGTCTAGGTACTGATGACAAGACACTTGTAAGGGTTGTGGTAACAAGGACT

GAGATTGATATGCAGTATATCAAGGCAGAATATTACAAGAAATACAAAAAGCCGTTGGCTGATGCTATC
CATTCCGAAACATCAGGCGGTTATCGGACGTTCCTGCTTTCTCTTGTTGGTAGCCATTAG
>TaAnn10-A
ATGGCTGCCCGTTCTCCTGCCGCCGCAGGGTTCGAGAACGAGTGCAGAGAGATCCACGGAGCATGCG
ACGAGCCGCGCCGCCTGAGCCGCCTCCTGGCTCACCGGAGCGCGTCGGAGAGGCAGCAGATCAAGG
CGACGTACCGCGCAATGTTCGGCGAGGACCTCGCCGGCAGGCTCCATAAAACCCTCACGGCCAATCA
GGACAACGAGCTCTGCAACCTGCTCTACCTGTGGATGCTTGACCTGGCGGAGCGAGACGCGATCATG
GCGAGGGACGCCATCGAGAGCGGCGCGGCGGCCGATTACCGCGCCCTTGTCGAGGTCTTCACGCGGC
GGAAGCAGGACCAGCTCTTCTTCACCAAGCAGGCGTACCTGGCCAGGTTCAAGAAGAACCTGGAGCA
GGACATGGTCACCGACCCCTCGCACCCTTATCAGAGACTATTGGTAGCTCTGGCAACCTCCCACAAGT
CGCACCACGATGAACCCAGCCAGCACATCGCGAAATGCGACGCCAGGCGATTGTACGACGCCAAGAA
CGGCGGCGGCACGGGATCGGTCGACGAGGCCGCCATCCTCGAGATGTTCAGCAAGAGGAGCATCCCA
CAGCTCAGGCTGGCGCTCTGCAGTTACAAGCACATATATGGGCATGACTTCACCAAAGCACTGAAGAA
GAATTTGGCTCGTGATTTTGAAGAGTCTCTGAGAGTTGTTGTCAAGTGCATCTACACTCCTTCCAAGTA
TTACTGCAAGTTACTGCAGAGAAGCATGCAACGCCCAGAAGCCGATAAAAGGTTGGTTACGAGGGCT
ATCTTGGGCAGCGACGACGTCGGCATAAACGAGATCAAGTTGGCGTTTAAGAGTAACTTCGGAAAGA
ACCTTGCAGATTTCATCCATGAAAGCTTACCACAGAGTGATTACAGAGAGTTTCTTTGGTTATGGCAAG
GGGGCCAGTGCCTAGTAGCATCATAA
>TaAnn10-B
ATGGCTCTCTCTCCCTCTCTCCCTTATGCATCTCTGCACTGGTTCACACATCGAAAGCCTCAGCACGGC
GCTGAAGTGCCGAGCATGGCCGCCCGTTCTCCTGCCGCCACAGGGTTCGAAAACGAGTGCAGAGAGA
TCCATGGCGCCTGCGACGAGCCGCGCCGCCTGAGCCGCCTCCTGGCTCGCCGGAGCACATCGGAGAG
GCAGCAGATCAAGGCGACGTACCGCGCAATGTTCGGCGAGGACCTCGCCGGCAGGCTCCATAAAACC
CTCACGGCCAATCAGGACAACGAGTCTCAGCATGGCCTCCCTCTCCAGCTCTGCAATCTGCTCTACCT
GTGGATGCTTGACCTGGCGGAGCGAGACGCGATCATGGCGAGGGACGCCATCGAGAGCGGCGCGGCG
GCCGATTACCGCGCCCTTGTCGAGGTCTTCACGCGGCGGAAGCAGGACCAGCTCTTTTTCACCAAGCA
GGCGTACCTGGCCAGGTTCAAGAAGAACCTGGAGCAGGACATGGTCACCGACCCCTCGCACCCTTAT
CAGAGACTATTGATAGCTCTGGCAACCTCCCACAAGTCGCACCACGATGAACCCAGCCAGCACATCGC
GAAATGCGACGCCAGGCGATTGTACGACGCCAAGAACGGCGGCGGCACGGGATCGGTCGACGAGGC
CGCCGTCCTCGAGATGTTCAGCAAGAGGAGCATCCCACAGCTCAGGCTGGCGCTCTGCAGTTACAAG
CACATATATGGGCATGACTTCACCAAAGCACTGAAGAAGAATTTGGCTCGTGATTTTGAAGAGTCTCT
GAGAGTTGTTGTCAAGTGCATCTACACTCCTTCCAAGTATTACTGCAAGTTACTGCAGAGAAGCATGC
AACGCCCAGAAGCCGATAAAAGGTTGGTTACGAGGGCTATCTTGGGCAGCGGCGACGTCGGCATAAA
CGAGATCAAGTTGGCGTTTAAGAGTAACTTCGGAAAGAACCTTGCAGATTTCATCCATGAAAGCTTAC
CACAGAGTGATTACAGAGAGTTTCTTTGGTTATGGCAAGGGGGCCAGTGCCCAGTAGCATCATAA
>TaAnn10-D
ATGGCTGCCCGTTCTCCTGCCGCCGCAGGGTTCGAGAACGAGTGCAGAGAGATCCACGGAGCATGCG
ACGAGCCGCGCCGCCTGAGCCGCCTCCTGGCTCACCGGAGCGCGTCGGAGAGGCAGCAGATCAAGG
CGACGTACCGCGCAATGTTCGGCGAGGACCTCGCCGGCAGGCTCCATAAAACCCTCACGGCCAATCA
GGACAACGAGTCTCAGCATGGCCTCCCTCTCCAGCTCTGCAACCTGCTCTACCTGTGGATGCTTGACC
TGGCGGAGCGAGACGCGATCATGGCAAGGGACGCCATCGAGAGCGGCGCGGCGGCCGATTACCGCGC
CCTTGTCGAGGTCTTCACGCGGCGGAAGCAGGACCAGCTCTTCTTCACCAAGCAGGCGTACCTGGCC
AGGTTCAAGAAGAACCTGGAGCAGGACATGGTCACCGACCCCTCGCACCCTTATCAGAGACTATTGGT
AGCTCTGGCAACCTCCCACAAGTCGCACCACGATGAACCCAGCCGGCACATCGCGAAATGCGACGCC
AGGCGATTGTACGACGCCAAGAACGGCGGCGGCACGGGATCGGTCGACGAGGCCGCCATCCTCGAGA

TGTTCAGCAAGAGGAGCATCCCACAGCTCAGGCTGGCGCTCTGCAGCTACAAGCACATATATGGGCAT
GACTTCACCAAAGCACTGAAGAAGAATTTGGCTGGCGATTTTGAAGAGTCTCTGAGAGTTGTTGTCA
AGTGCATCTACACTCCTTCCAAGTATTACTGTAAGTTACTGCAGAGGAGTATGCAACGCCCAGAAGCT
GATAAAAGGTTGGTTACGAGGGCTATCTTGGGCAGCGACGACGTCGGCATAAACGAGATCAAGTTGGC
GTTCAAGAGTAACTTTGGAAAGAACCTCGCAGATTTCATCCATGAAAGCTTACCACAGAGTGATTACA
GAGAGTTTCTTTGGTTATGGCAAGGGGGCCAGTGCCTAGTAGCATCATAA
>TaAnn11-A
ATGGCGACGCTCTCCGTCCCCGCCGCCGTCCCGGCCGTCGCCGAGGACTGCGAGCAGCTGCGCAAGG
CGTTCGCGGGGTGGGGCACCAATGAGCGCCTCATCGTCTCCATCCTGGCGCACCGCGACGCCGCGCA
GCGCCGCGCCATCCGCCGCGCCTACGCCGAGGCCTACGGCGAGGAGCTGCTCCGCGCCATCGGCGAC
GAGATCCACGGCAAGTTCGAGAGGGCGGTGATCCAGTGGACGCTGGACCCGGCGGAGCGGGACGCG
GAGCTGGCCAACGAGGAGGCCAAGAAGTGGCAGCCGGGGGGCCGCGCGCTCGTCGAGATCGCCTGC
GCGCGCACCCCCGCGCAGCTCTTCGCCGCCAAGCAGGCCTACCACGACCGCTTCAAGCGCTCGCTCG
AGGAGGACGTCGCCGCGCACGTCACCGGCGACTTCCGCAAGCTTTTGGTGCCTCTTGTAAGCGCATAC
CGCTACGATGGCCCGGAGGTGAACACATCGTTGGCACACTCTGAAGCCAAATTACTCAACGGAAAGA
TCAACGAGAAGGCCTACAGCGATGATGAGGTCGTCAGGATTCTCACCACAAGGAGCAAGGCTCAGCT
ACTTGCAACATTCAACAGCTACAATGATCAGTTCAGTCACCCCATCACCAAGGACCTCAAGGAAGACC
CAAAGAACGAGTTCCTGTCAACCCTCAGGGCGATCATAAGGTGTTTCACCTGTCCTGACAGATACTTT
GAGAAGGTCGTCCGGTTGGCCCTTGGAGGCGTCGGCACAGACGAGGACACCCTTACGAGGGTCATAA
CCACTCGCGCTGAGGTGGACCTGAAGGTGATAAAGGAGGCGTACCAGAAGAGGAACAGCGTCCCGC
TGGAGAAGGCTGTTTCCAAGGAAACAAGCAGAGACTATGAAGATATGATGCTTGCACTTCTGGGGGC
AGAGTACTGA
>TaAnn11-B
ATGGCGACGCTCTCCGTCCCCGCCGCCGTCCCGGCCGTCGCCGAGGACTGCGAGCAGCTGCGCAAGG
CGTTCGCGGGGTGGGGCACCAACGAGCGCCTCATCGTCTCCATCCTGGCGCACCGCGACGCCGCGCA
GCGCCGCGCCATCCGCCGCGCCTACGCCGAGGCCTACGGCGAGGAGCTGCTCCGCGCCATCGGCGAC
GAGATCCACGGCAAGTTCGAGAGGGCGGTGGCCCAGTGGACGCTGGACCCGGCGGAGCGGGACGCA
GAGCTGGCCGGCGAGGAGGCCAAGAAGTGGCAGCCGGGGGGCCGCGCGCTCGTGGAGATCGCCTGC
GCGCGCACCCCCGCGCAGCTCTTCGCCGCCAGGCAGGCCTACCACGACCGCTTCAAGCGCTCGCTCG
AGGAGGACGTCGCCGCGCACGTCACCGGCGAATTCCGCAAGCTTTTGGTGCCTCTTGTAAGCGCATAC
CGCTACGATGGCCCGGAGGTGAACACATCGTTGGCACACTCTGAAGCCAAAATACTCAATGGAAAGAT
CAACGAGAAGGCCTACAGTGATGATGAGATCATCAGGATTCTCACTACAAGGAGCAAAGCTCAGCTAC
TTGCAACATTCAACAGCTACAATGATCAGTTCAGTCACCCCATCACTAAGGACCTCAAGGAAGACCCC
AAGAACGAGTTCCTCTCAACCCTGAGGGCGATCATAAGGTGTTTCACTTGCCCTGACAGATACTTTGA
GAAGATCATCCGGTTGGCCCTTGGAGGCGTCGGCACAGACGAGAACACCCTTACGAGGGTCATAACC
ACTCGCGCCGAGGTGGACCTGAAGGTGATAAAGGAGGCGTACCAGAAGAGGAACAGCGTCCCGCTG
GACAAGGCTGTTTCTAAGGAAACAAGCAGAGACTACGAGGACATGATGCTTGCACTTCTGGGGGCAG
AGTACTGA
>TaAnn11-D
ATGGCGACGCTCTCCGTCCCCGCCGCCGTCCCGGCCGTCGCCGAGGACTGCGAGCAGCTGCGCAAGG
CGTTCGCGGGGTGGGGCACCAACGAGCGCCTCATCGTCTCCATCCTGGCGCACCGCGACGCCGCGCA
GCGCCGCGCCATCCGCCGCGCCTACGCCGAGGCCTACGGCGAGGAGCTGCTCCGCGCCATCGGCGAC
GAGATCCACGGCAAGTTCGAGAGGGCGGTGACCCAGTGGACGCTGGACCCGGCGGAGCGGGACGCG
GAGCTGGCCGGCGAGGAGGCCAAGAAGTGGCAGCCGGGGGGCCGCGCGCTCGTCGAGATCGCCTGC
GCGCGCACCCCCGCGCAGCTCTTCGCCGCCAAGCAGGCCTACCACGACCGCTTCAAGCGCTCGCTCG

AGGAGGACGTCGCCGCGCACGTCACCGGCGACTTCCGCAAGCTTCTGGTGCCTCTTGTAAGCGCATAC
CGCTACGATGGTCCGGAGGTGAACACATCGTTGGCACACTCTGAAGCCAAAATACTCAATGGAAAGAT
CAACGAGAAGGCCTACAGTGATGATGAGATCGTCAGGATTCTCACTACAAGGAGCAAGGCTCAGCTA
CTTGCAACATTCAACAGCTACAATGATCAGTTCAGTCATCCCATCACTAAGGACCTCAAGGAAGACCC
AAAGAACGAGTTCCTCTCAACCCTGAGGGCGATCATAAGGTGTTTCACTTGCCCTGACAGATACTTTG
AGAAGGTCGTCCGGTTGGCCCTTGGAGGCGTCGGCACAGACGAGAACACCCTTACGAGGGTCATAAC
CACTCGCGCCGAGGTGGACCTGAAGGTGATAAAGGAGGCGTACCAGAAGAGGAACAGCGTCCCGCT
GGAGAAGGCTGTTTCCAAGGAAACAAGCAGAGACTATGAAGATATGATGCTCGCACTTCTGGGGGCA
GAGTATTGA
>TaAnn12-A
ATGGCGACGCTCAAGGTCCCCTCCAACGTCCCGGCCCTCGCCGACGACTGCGACAACCTCCGCAAGG
CCTTCCAAGGGTGGGGCACGAACGAGGCGCTCATCATCTCCATCCTCGGCCACCGCGACGCGTCGCA
GCGCCGCGCCATCCGCAAGCACTACGCCGACACCTACGGCGAGGAGCTGCTCCGGAGCATCACCGAC
GAGATCTCCGGCGACTTCGAGAGGGCCGTGATCCTGTGGACGCTGGACCCGGCGGAGCGGGACGCGG
TGCTCGCCAACGAGACCGCCAGGAAGTGGCACCCCGGGAACCCCGTGCTGGTCGAGATCGCCTGCGC
GCGCGGCTCCAAGCAGCTCTTCGCCGTCAGGCAGGCCTACCATGACCGCTTCAAGCGCTCGCTCGAG
GAGGACGTCGCTTCCCACGTCACCGGCGACTTCCGCAAGCTGTTGGTGCCACTTGTAAGCTCACACC
GCTACGAGGGACCAGAGGTCAACACAAGGTTGGCTCATTCAGAAGCCAAATTACTACACGAGAAGAT
TGAGCATAAGGCTTATGCTGATGATGAGGTCATCAGAATTCTCACCACCAGGAGCAAAGCTCAGCTGC
TTGCAACATTCAATAATTACAATGATACATTTGGTCACCCGATCACTAAGGATCTGAAGGCTGACCCCA
AGGATGAGTTCCTCAAGACCCTGCGGGCGGTCATCCGGTGCTTCACCTGCCCTGATAGGTACTTTGAG
AAGGTGGCCAGGGTGGCCATTGCAGGGAATGGAACCGATGAGAACTCCCTCACTAGGGTCATCACCA
CCCGCGCCGAGGTGGACCTGAAGCTGATCAAAGAGGCGTACCAGAAGAGGAACAGTGTTCCCCTGG
AGAAGGCCGTCGCAGGCGACACCTCCGGCGACTACGAGAGCATGCTCCTTGCCCTCCTGGGGAAGGA
GTGA
>TaAnn12-D
ATGGCGACGCTCAAGGTCCCCTCCAACGTCCCGGCCCTCGCCGACGACTGCGACAACCTCCGCAAGG
CCTTCCAAGGGTGGGGCACGAACGAGGCGCTCATCATCTCCATCCTGGGCCACCGCGACGCCGCGCA
GCGCCGCGCCATCCGCAAGCACTACGCCGACACCTACGGCGAGGAGCTGCTCCGGAGCATCACCGAC
GAGATCTCCGGCGACTTCGAGAGGGCCGTGATCCTGTGGACGCTGGACCCGGCGGAGCGGGACGCGG
TGCTCGCCAACGAGACCGCCAAGAAGTGGCACCCCGGGAACCCCGTGCTGGTCGAGATCGCCTGCGC
GCGCGGCTCCAAGCAGCTCTTCGCTGCCAGGCAGGCCTACCATGACCGCTTCAAGCGCTCGCTCGAG
GAGGACGTCGCTGCACACGTCACCGGCGACTTCCGCAAGCTGTTGGTGCCACTTGTAAGCTCACACC
GCTATGAGGGACCAGAGGTCAACACAAGGTTGGCTCATTCAGAGGCCAAATTACTGCATGAGAAGATT
GAGCATAAGGCTTATGGTGATGATGAGGTCATCAGAATTCTCACCACTAGGAGCAAAGCTCAGCTGCT
TGCAACATTCAATAATTACAATGATACATTTGGTCACCCAATCACTAAGGATCTGAAGGCTGACCCCAA
GGATGAGTTCCTCAAGACCCTGCGGGCTGTTATCCGGTGCTTCACCTGCCCTGATAGGTACTTTGAGA
AGGTGGCCAGGCTGGCCATTGCAGGGAATGGAACAGATGAGAACTCCCTCACTAGGGTCATCACCAC
CCGTGCCGAGGTGGACCTGAAGCTGATCAAAGAGGCGTACCAGAAGAGGAACAGTGTTCCCCTGGA
GAAGGCCGTCGCAGGCGACACTTCCGGCGACTATGAGAGCATGCTCCTTGCCCTCCTGGGGAAGGAG
TAG

(2). barely (*H.vulgare*)
>HvAnn1 MLOC_15770.2
MASLSVPPVLTPPREDAIALHKAFKGLGCDTTMVINILAHRDTAQRVLIQQEYKAIYHEDLYHRLATELSG

NHKNAMLLWVLDPVGRDATILNQALNGDITDLRAATEVICSRTPSQLQIMKQTYRARFGCYLEHDITERT
YGDHQKLLLAYLGVPRNEGPEVDPSVVTDDARELYRTGEKRVGTDERAFIRIFSERSWAHLASVAKAYQH
MYARSLEKAVKSETAGNFQFGLLTILRCADTPAKYFAKVLHKAMKGLGTSNAALIRVVVTRTEVDMKYIK
VEYHNKYKGSLAEAIHSETSGNYRTFLLSLVGQDR
>HvAnn2 MLOC_54933.3
MCGWCCCLDLECIHNIPPLSLLFLHFSSAPSTGREGIAERAPSMASISVPDPVPSPTEDAENIRKAVQGWGT
DENALIEILGHRTAAQRAEIAVAYEGLNDKTLLRTLQDELSGHFKGAMTLWAMDPVARDAKLAYKALRKK
GGDRHAWVLIEVACASSPDHLVAVRKAYCSAYDSSLEEDVAACSLYKEPLKQFLVRLVSSYRYAGDLVDGE
LARAEAAELHGAVAAKKQPLHGDVVRIVSSRSKPQLKATFEHYKRQHGKPIHEVLEGNRNDQLSAMLKT
AVWCLTSPEKHFAEVIRTSIIGLGTDEESLTRAIVSRAEVDMKKVKEEYKVRYKTTVTKDVVGDTSGYYQ
GILLTLIGPE
>HvAnn3 MLOC_54932.1
MADEVQALTKAFSGLGGLGVDERTMVAALANWRKQPEKRSGFRKSFPGLFKEHGGVMERCEDEYMLHL
AAEFSRFKNLMVLWAMHPWERDARLAHHVLHQAHPSAIVVEIACTRSAEELLGARKAYMALFHHSLEED
VAYRAKDKPYCSLLVGLVSAYRYEGPKVSDDTAKAEAKALGAAVKSADGGKLVENDEVVRILTTRSKPHL
VQTFKYYKELHGKHIEEDLGSEEALREAVQCLATPERYFSQVMAAALREGADHHGKEALSRVAVTRSDV
DMDGIRAAYQEQFGAKLEDAVAGSAHGHFKDALVSLIVGK
>HvAnn5 MLOC_15543.3
MSAIAVPSPVPSPSDDAEGIRKALQGWRADKEALVRILARRTAAQRSAIRRAYAFLFREPLLNSFRQRLSRQ
YCPVTVDFWKAIILWTMDPAERDANLVHGALRRRGDGDHLAVLVEVSCASDPDHLVAVRRAYRSLFGCS
VEEDLASCPALQQPLRKMLVSLVSSYRYGGDRVDADVAKLEASQLSEAVRKKQPHHDEVVRILSTRSKPQ
LRATFRRYREDHGTDIVDDIDSRCSSQFARTLKSAVWCLTSPEKHFAEMIRESVVGLGTYEDMLTRVVVSR
AEVDMEQIKEEYRARFKTTVTCDVVDDTSFGYKDILLALVGCEEE
>HvAnn6 MLOC_55134.1
MRTAASPSIGDPPTGKPGFSRFKTLNPIVPVRTQHLHCTTSGSHRHRQPTHVRLLRSMASLSVPPVLTSPRH
DAIALHRAFKGFGCDSTTVINILAHRDATQRALIMQEYRAIYHQDLYHRLSTELSGNHKKAMLLWVLDPV
GRDAAILNQSLNGDITDLRAATEVICSRTPSQLHIMRQAYRARFGCYLEHDVAERTYGDHQKLLLAYLGVP
RYEGPEVDPAAAARDARDLYRAGEKRLGTDERAFIRVFTERSWAHLAAVAGAYRHLYARSLDKAVKSETS
GTFELGLLTILRCAESPARHFAKVLHKAMKGLGTSDTALIRVVVTRAEVDMQYIKAEYHKKYKRSLADAI
HSETSGNYRTFLLSLVGRDR
>HvAnn7 MLOC_3056.1
MLPPTKKTLQFLPPPLPPSTESSSSSVRASESREQSAGEMATIAVPTPVPSPAADAETLRNAVQGWGTDEKA
LVEILGRRTAAQRAEIRRAYASLYKESLLARLHGELSGHFQKAMVLLATEPAERDAKLAREALGRRRGDD
RDAWMLIETSCAAAPDHLVAVRRAYRSLHGSSLEEDVAACPAFQEPLRKLLVSLVRSYRCGEESVDMDVA
RLEAAQLAEAIRRKKQPHGGEVVRIVSTRSKPQLAATLRCYKEQHGSDIEEDMKQYSSSQFARMLKIAVW
CLTSPEKHFAEVIRYSILGLGTDEDALTRAIVSRADIDMKMIKQEYRVRFKTTVTDDVVGDTSGYYMEILL
ALVGKE
>HvAnn8 AK370408
MQQFLAAQASSNGSTAAAAAAPPASASPLRSRPPRREQPSQTTVKDKEATMADEHQELTKAFSGMGGLG
VEETALVSALGRWRKQPEKRAQFRRGFPGFFTPAAAAGAGAIERCSDDYVSHLKTEFARFKSLMVLWAM
HPWERDARWAHRALHKKHHPASVLVELACTRTADELLGARRAYHALYHRSLEEDVAYRVKDADANRLL
VGLVSAYRYEGPRVDEGLAREEAAALAGAKAQSELVARVLATRSKPQLRATFRLYRELHGKPLEEEFGGE
APCLREAVRCLESPARYFGEVIDGAFKEGADKQAKAALTRVVVSRSDADMEEIKDAYLKHHGAKLVDAV
AKNTHGHYRDALLAMIGK
>HvAnn9 AK249691

MASLTLPPAPPNPRQDAIDLQKAFKGFGCDSTTVSNILAHRDSMQRGYIQQEYKTMYSEELSRRISSELSG
NHKKAMSLWILDPAGRDATVLKEALSAESLDLKAATDIICSRTPSQLQIMKQTYYAKFGTYLEHDISQQTS
GDHQKILLAYVGIPRYEGPEVDPTIVTHDAKDLYKAGEKKLGTDEKTFIRIFTERSWAHMAAVASAYQHM
YDRSLQKVVKSETSGNFEVALITILRCAENPAKYFAKVLRKSMKGLGTDDKTLVRVVVTRTEIDMQYIKAE
YYKKYKKPLADAIHSETSGGYRTFLLSLVGSH
>HvAnn10 MLOC_54650.4
MASRSPATAGFESECREIHGACDEPRRLIRYLAHRSAPERQQIKATYRTMFGEDPVGRLHKTLTANQDNEL
CNLLYLWMLDLAERDAIMARDAIESGAAADYRALVEIFTRRKQDQLFFTKQAYLARFKKNLEQDMVTDP
SHPYQRLLVALATSHKSHHDEPSRHIAKCDARRLYDAKNGATGSVDEAAILEMFSKRSIPQLRLALCSYKH
IYGHDFTKALKKNSAGDFVESLKVVVKCIHSPSNYYCKLLQRSMQRPETNKRLVARAILGSDDVGVNEIK
LAFKSNFGKNLEDFIHESLPQSDYRDFLWIWQGGQCPVAS
>HvAnn11 MLOC_51591.2
MATLSVPAAVPAVAEDCEQLRKAFAGWGTNERLIISILAHRDAAQRRAIRKHYADAYGEELLRSITDEIHGK
FERAVIQWTLDPAERDAVLANEEAKKWNPGGRALVEIACARTPAHLFAAKQAYHDRFKRSLEEDVAAHVT
GDFRKLLVPLVSAYRYDGPEVNTSLAHSEAKILHAKISEKAYSDDEVVRILTTRSKAQLLATFNSYNDQFSH
PITKDLKEDPKDEFLSTLRAIIRCFTCPDRYFEKVIRLALGGVGTDEHTLTRVITTRAEVDLKVIKEAYQKRN
SVPLEKAVSKDTTRDYEDMMLALLGAEY
>HvAnn12 AK248515
MATLKVPSNVPALADDCDNLRKAFQGWGTNEALIISILGHRDAAQRRAIRKHYADTYGEELLRSITDEISG
DFERAVILWTLDPAERDAVLANETAKKWHPGNPVLVEIACSRGSAQLFAVRQAYHDRFKRSLEEDVAAHV
TGDFRKLLVPLVSSYRYEGPEVNTRLAHSEAKILHEKIEHKAYGDDEIIRILTTRSKAQLLATLNNYNDTFG
HPITKDLKADPKDEFLKTLRAVIRCFTCPDRYFEKVARLAIAGNGTDENSLTRVITTRAEVDLKLIKEAYQK
RNSVPLEKAVAGDTSGDYETMLLALLGKEN
>HvAnn1 MLOC_15770.2
ATGGCGAGCCTGAGCGTGCCTCCGGTGCTCACGCCTCCGCGCGAGGACGCCATCGCTCTCCACAAGG
CCTTCAAAGGGCTCGGTTGCGACACCACGATGGTGATCAACATACTTGCTCACCGCGACACGGCGCA
GCGCGTGCTCATCCAGCAGGAGTATAAAGCCATATACCATGAGGATCTCTACCATCGCCTAGCAACTGA
GCTGAGTGGAAACCACAAGAATGCTATGCTGCTTTGGGTCCTCGACCCCGTGGGGCGTGACGCCACCA
TATTGAACCAAGCTCTCAATGGCGACATCACCGACCTGAGAGCCGCCACCGAGGTAATCTGCTCCAGG
ACGCCGTCGCAGCTGCAGATCATGAAGCAAACCTACCGCGCGAGGTTCGGTTGCTACCTCGAGCATGA
CATCACCGAACGCACCTATGGTGATCATCAGAAGCTTTTGCTTGCGTACCTTGGAGTCCCGCGAAACG
AAGGCCCGGAAGTAGACCCTTCGGTGGTGACAGACGACGCGAGGGAGCTGTATCGAACCGGCGAGA
AGAGGGTGGGCACCGATGAGCGGGCCTTCATCCGCATCTTCAGCGAACGCAGCTGGGCGCACCTGGC
GTCCGTTGCCAAAGCTTACCAGCACATGTACGCCCGGTCCCTGGAGAAGGCAGTGAAGAGTGAGACA
GCAGGGAACTTCCAGTTCGGGCTGCTGACCATCCTCAGGTGCGCCGACACTCCGGCGAAGTACTTTGC
CAAGGTCCTGCACAAGGCGATGAAAGGCCTGGGCACCAGCAACGCGGCACTCATACGGGTGGTGGTG
ACGAGGACCGAGGTCGACATGAAGTACATCAAAGTAGAGTACCACAACAAGTACAAGGGGTCGCTGG
CCGAAGCTATACACTCAGAGACGTCAGGGAACTACAGAACCTTCCTCCTCTCGCTCGTCGGCCAGGAC
CGC
>HvAnn2 MLOC_54933.3
ATGTGTGGCTGGTGCTGCTGCCTCGACCTCGAGTGCATCCATAACATCCCGCCCCTCAGTCTCCTCTTC
CTCCACTTCTCCTCGGCTCCATCAACGGGAAGGGAGGGGATAGCAGAACGTGCTCCCTCCATGGCCTC
CATCTCGGTCCCGGACCCCGTCCCTTCCCCGACCGAGGACGCAGAGAACATAAGAAAGGCGGTACAA
GGGTGGGGGACGGACGAGAATGCGCTGATCGAGATCCTAGGCCACCGGACGGCGGCGCAGCGCGCG
GAGATCGCCGTGGCCTACGAGGGCCTCAACGACAAGACCCTCCTCCGCACGCTCCAAGACGAGCTCT

CCGGCCACTTCAAGGGCGCCATGACGCTGTGGGCGATGGACCCGGTGGCGCGGGACGCCAAGCTGGC
CTACAAGGCCCTGAGGAAGAAGGGCGGCGACCGGCACGCCTGGGTGCTCATCGAGGTGGCCTGCGC
GTCGTCGCCGGACCACCTCGTCGCCGTCAGGAAGGCCTACTGCTCCGCCTACGACTCGTCCCTCGAGG
AGGACGTCGCCGCCTGCTCGCTCTACAAGGAACCCCTCAAGCAGTTCCTGGTGCGGTTGGTGAGCTC
GTACCGCTACGCCGGCGACCTCGTCGACGGCGAGCTGGCGAGGGCGGAGGCGGCCGAGCTGCACGG
CGCGGTGGCGGCCAAGAAGCAGCCGCTGCACGGGGACGTGGTGCGCATCGTCAGCTCCAGGAGCAA
GCCGCAGCTCAAGGCCACGTTCGAGCACTACAAGCGACAGCACGGCAAGCCCATCCACGAGGTTCTC
GAGGGGAACCGGAATGACCAGCTCTCGGCCATGCTGAAAACTGCGGTTTGGTGCTTGACATCGCCGG
AGAAACATTTCGCAGAGGTGATCCGGACCTCGATCATAGGGCTCGGCACCGACGAGGAGTCGCTGAC
CAGAGCCATCGTCTCGCGCGCGGAGGTCGACATGAAGAAAGTCAAGGAGGAATACAAGGTGAGGTAC
AAGACGACGGTGACCAAGGACGTCGTCGGCGACACTTCCGGGTACTACCAGGGCATCCTGCTCACCC
TCATCGGGCCAGAG
>HvAnn3 MLOC_54932.1
ATGGCTGACGAAGTTCAGGCGCTCACCAAGGCCTTCTCAGGTCTGGGGGGTCTGGGCGTGGACGAGC
GGACGATGGTGGCGGCGCTGGCGAACTGGCGGAAGCAGCCGGAGAAGCGGTCGGGGTTCCGGAAGA
GCTTCCCGGGGCTGTTCAAGGAGCACGGCGGCGTGATGGAGCGGTGCGAGGACGAGTACATGCTGCA
CCTGGCCGCCGAGTTCTCCCGGTTCAAGAACCTGATGGTGCTGTGGGCGATGCACCCGTGGGAGCGC
GACGCCCGCCTCGCGCACCACGTCCTCCACCAGGCCCACCCCTCCGCCATCGTCGTGGAGATCGCCTG
CACCCGCTCCGCCGAGGAGCTCCTCGGCGCGCGCAAGGCCTACATGGCGCTCTTCCACCACTCCCTCG
AGGAGGACGTCGCCTACCGCGCCAAGGACAAGCCCTACTGCAGCCTGCTGGTGGGGCTGGTGAGCGC
GTACCGGTACGAAGGGCCCAAGGTGAGCGACGACACGGCCAAGGCGGAGGCCAAGGCGCTGGGAGC
GGCGGTGAAGAGCGCCGACGGCGGCAAGCTGGTGGAGAACGACGAGGTGGTAAGGATCCTCACCAC
CAGGAGCAAGCCTCACCTCGTCCAGACCTTCAAGTACTACAAGGAGCTGCACGGCAAGCACATCGAG
GAGGATCTCGGAAGCGAGGAGGCTCTGAGGGAGGCCGTGCAGTGCCTGGCGACGCCGGAGAGGTAC
TTCAGCCAGGTGATGGCGGCGGCGCTGAGGGAGGGCGCGGACCACCACGGCAAGGAGGCCCTGTCG
CGGGTGGCGGTGACGCGGTCGGACGTGGACATGGACGGCATCAGGGCGGCCTACCAGGAGCAGTTCG
GGGCCAAGCTCGAGGACGCCGTCGCCGGCAGCGCGCACGGCCACTTCAAGGACGCGCTCGTCTCGCT
CATCGTCGGCAAG
>HvAnn5 MLOC_15543.3
ATGTCCGCCATCGCCGTCCCCAGCCCCGTGCCGTCTCCCTCCGACGACGCCGAGGGCATCAGGAAGGC
GCTCCAAGGATGGCGGGCGGACAAGGAGGCCCTGGTGCGCATACTGGCGCGGAGGACGGCGGCGCA
GCGGTCGGCGATCCGCCGCGCCTACGCCTTCCTCTTCCGGGAGCCCCTCCTCAACTCCTTCCGCCAGC
GGCTCTCCCGCCAGTACTGCCCCGTCACCGTCGACTTCTGGAAGGCGATCATCCTGTGGACGATGGAC
CCGGCGGAGCGGGACGCGAACCTGGTGCACGGCGCGCTGCGGCGGCGAGGGGACGGCGACCACCTC
GCCGTGCTGGTGGAGGTCTCGTGCGCGTCCGACCCCGACCACCTCGTGGCCGTCCGGCGCGCCTACC
GCTCCCTCTTCGGCTGCTCCGTGGAGGAGGACCTCGCCTCCTGCCCCGCGCTCCAGCAGCCGCTCCGG
AAGATGCTGGTGAGCCTCGTGAGCTCCTACCGCTACGGCGGTGACCGCGTCGACGCCGACGTGGCGA
AGCTGGAGGCATCGCAGCTCTCGGAGGCCGTCAGGAAGAAGCAGCCGCACCACGACGAGGTGGTGC
GGATCCTCAGCACCCGGAGCAAGCCCCAGCTCAGGGCCACGTTCCGGCGGTACAGGGAGGACCATGG
CACCGACATCGTCGACGACATCGACAGCCGCTGCAGCAGCCAGTTCGCCAGGACGCTGAAGAGCGCC
GTCTGGTGCCTGACCTCGCCGGAGAAGCACTTCGCGGAGATGATCAGGGAGTCGGTGGTGGGGCTGG
GGACGTACGAGGACATGCTGACCAGGGTGGTGGTGTCGCGGGCGGAGGTCGACATGGAGCAGATCAA
GGAGGAGTATAGGGCCAGGTTCAAGACCACGGTCACCTGCGACGTCGTCGACGACACCTCCTTCGGC
TACAAGGACATCCTGCTGGCCTTGGTTGGCTGCGAGGAAGAG
>HvAnn6 MLOC_55134.1

ATGCGCACGGCGGCGTCGCCATCCATCGGTGATCCCCCGACGGGAAAGCCCGGCTTCTCCCGCTTTAA
AACCCTCAACCCGATCGTCCCCGTACGCACTCAGCACCTGCACTGCACCACGAGCGGCAGCCACAGG
CACAGGCAGCCCACGCACGTACGCCTCCTCCGATCAATGGCGAGCCTGAGCGTGCCGCCGGTGCTCA
CGTCCCCGCGCCACGACGCCATCGCCCTCCACAGGGCCTTCAAAGGTTTTGGTTGCGACAGCACGAC
GGTGATCAACATCCTGGCGCACCGGGACGCGACGCAGCGGGCGCTCATCATGCAGGAGTACAGGGCC
ATCTACCACCAGGACCTGTACCACCGCCTCTCCACGGAGCTGAGCGGGAACCACAAGAAGGCGATGC
TGCTGTGGGTGCTGGACCCCGTGGGCCGCGACGCCGCCATCCTCAACCAGTCCCTCAACGGCGACAT
CACCGACCTGCGCGCCGCCACGGAGGTCATCTGCTCCCGGACGCCCTCGCAGCTGCACATCATGAGG
CAGGCCTACCGCGCCCGCTTCGGCTGCTACCTCGAGCACGACGTGGCCGAGCGCACCTACGGCGACC
ACCAGAAGCTCCTGCTGGCGTACCTGGGGGTCCCCCGGTACGAGGGCCCGGAGGTGGACCCGGCGGC
GGCGGCGCGCGACGCCAGGGACCTGTACCGGGCCGGCGAGAAGCGGCTGGGCACCGACGAGCGGGC
CTTCATCCGCGTCTTCACCGAGCGCAGCTGGGCGCACCTCGCCGCCGTCGCCGGCGCATACCGCCACC
TCTACGCGCGCTCCCTCGACAAGGCCGTGAAGAGCGAGACGTCGGGGACGTTCGAGCTCGGGCTGCT
CACCATCCTCCGCTGCGCCGAGAGCCCCGCGAGGCACTTCGCCAAGGTGCTGCACAAGGCGATGAAG
GGGCTGGGCACCAGCGACACGGCGCTCATACGGGTGGTGGTCACCAGGGCCGAGGTCGACATGCAGT
ACATCAAGGCCGAGTACCACAAGAAGTACAAGCGCTCGCTCGCCGACGCCATACACTCCGAGACGTC
GGGGAACTACCGGACCTTCCTCCTCTCCCTCGTCGGCCGCGACCGC

>HvAnn7 MLOC_3056.1

ATGCTCCCTCCCACCAAGAAAACGCTGCAATTCCTTCCTCCTCCCCTCCCTCCGTCCACTGAATCATCA
TCGTCCTCTGTGCGTGCGTCTGAGAGCAGAGAGCAGAGCGCGGGAGAGATGGCGACCATCGCCGTCC
CGACACCCGTGCCGTCCCCGGCCGCCGACGCCGAAACCCTCAGGAACGCCGTGCAAGGCTGGGGCA
CGGACGAGAAGGCCCTGGTGGAGATCCTCGGCCGGCGAACCGCCGCGCAGCGCGCCGAGATACGCC
GCGCCTACGCCAGCCTCTACAAGGAGTCCCTCCTCGCCCGCCTCCACGGCGAGCTCTCCGGCCACTTC
CAGAAAGCGATGGTGCTGCTGGCGACGGAGCCGGCGGAGAGGGACGCGAAGCTGGCGAGGGAGGC
GCTAGGCCGGCGGCGTGGCGACGACAGGGACGCGTGGATGCTCATCGAGACCTCCTGCGCCGCCGCG
CCGGACCACCTCGTCGCCGTCCGCCGCGCGTACCGGTCCCTCCATGGATCCTCCCTGGAGGAGGACGT
GGCCGCCTGCCCCGCGTTCCAAGAGCCGCTCAGGAAGCTGCTGGTGAGCCTGGTGAGGTCGTACCGG
TGCGGGGAGGAGAGCGTGGACATGGACGTGGCGAGGCTGGAGGCCGCGCAGCTGGCGGAGGCCATC
CGGAGGAAGAAGCAGCCGCACGGCGGCGAGGTCGTCCGGATCGTCAGCACCAGGAGCAAGCCGCAG
CTCGCGGCCACGCTCCGGTGCTACAAGGAACAACACGGCAGCGACATCGAAGAGGACATGAAGCAGT
ACAGCAGCAGCCAGTTTGCAAGGATGCTCAAGATTGCTGTCTGGTGTCTCACGTCTCCTGAAAAGCAC
TTTGCAGAGGTTATCAGGTACTCAATCCTAGGACTTGGAACGGACGAGGACGCACTCACCAGGGCGAT
CGTGTCCAGGGCTGACATCGACATGAAGATGATCAAGCAAGAGTACAGGGTCAGATTCAAGACGACC
GTAACAGACGATGTCGTCGGCGATACCTCGGGGTATTACATGGAGATCTTGCTGGCCTTGGTAGGGAA
GGAG

>HvAnn8 AK370408

ATGCAGCAGTTTCTTGCAGCACAGGCCAGCAGTAACGGCAGCACTGCAGCAGCAGCAGCAGCTCCAC
CGGCGTCGGCGTCGCCCCTGCGTTCCAGGCCGCCGAGGAGGGAGCAGCCAAGCCAGACCACCGTCA
AGGACAAGGAGGCGACCATGGCCGACGAGCACCAGGAACTCACCAAGGCCTTCTCAGGGATGGGCG
GCCTGGGCGTGGAGGAGACGGCGCTGGTGTCGGCCCTGGGCCGGTGGAGGAAGCAGCCGGAGAAGC
GGGCGCAGTTCCGCAGGGGCTTCCCGGGCTTCTTCACGCCGGCGGCGGCGGCGGGGGCGGGGGCGAT
CGAGCGCTGCTCGGACGACTACGTGAGCCACCTCAAGACGGAGTTCGCCCGGTTCAAGAGCCTCATG
GTGCTGTGGGCGATGCACCCGTGGGAGCGCGACGCGCGCTGGGCGCACCGCGCGCTGCACAAGAAG
CACCACCCGGCGTCCGTGCTCGTCGAGCTCGCGTGCACCCGCACCGCCGACGAGCTCCTCGGCGCCC
GCCGCGCCTACCACGCGCTCTACCACCGCTCCCTCGAGGAGGACGTGGCGTACCGCGTCAAGGACGC

CGACGCCAACCGCCTGCTCGTCGGGCTCGTCAGCGCCTACCGCTACGAGGGCCCCCGCGTCGACGAG
GGGCTCGCCAGGGAGGAGGCCGCGGCGCTCGCCGGCGCCAAGGCCCAGAGCGAGCTCGTGGCGCGG
GTGCTCGCCACCAGGAGCAAGCCGCAGCTCCGGGCGACCTTCAGGCTGTACAGGGAGCTCCACGGCA
AGCCGCTGGAGGAGGAGTTTGGCGGCGAGGCGCCGTGCTTGCGGGAGGCCGTGAGGTGCCTCGAGT
CCCCGGCGAGGTACTTCGGCGAGGTGATCGACGGGGCGTTCAAGGAGGGCGCGGACAAGCAGGCCA
AGGCCGCCTTGACCCGCGTCGTCGTGTCCCGGTCCGACGCCGACATGGAGGAGATCAAGGACGCCTA
CCTGAAACACCACGGGGCCAAGCTCGTGGACGCCGTCGCCAAGAACACCCACGGACATTACAGGGA
CGCGCTGCTCGCCATGATCGGAAAG

>HvAnn9 AK249691

ATGGCGAGCCTCACCTTGCCGCCGGCGCCCCCGAACCCCCGCCAGGACGCCATCGACCTCCAAAAGG
CCTTCAAAGGGTTTGGCTGTGATAGTACAACAGTTTCAAATATACTTGCTCACCGTGACTCAATGCAAC
GTGGATACATTCAACAGGAATACAAAACTATGTATTCCGAGGAACTTTCGCGTCGTATATCGTCTGAAC
TCAGTGGAAACCACAAGAAAGCAATGTCGCTCTGGATTCTTGATCCTGCTGGACGTGATGCGACTGTT
CTGAAAGAAGCTCTCAGTGCTGAAAGTCTTGATCTGAAAGCAGCTACTGATATAATATGTTCGAGGAC
ACCATCACAGCTGCAAATAATGAAACAGACATATTATGCAAAGTTTGGTACTTATCTTGAGCATGACATT
AGTCAGCAAACATCCGGCGATCATCAGAAGATCTTACTAGCCTATGTGGGCATTCCACGCTACGAAGG
TCCAGAGGTTGATCCCACTATAGTGACACATGATGCGAAGGACCTCTACAAAGCTGGTGAGAAGAAG
CTGGGCACAGATGAGAAAACCTTCATCCGCATCTTCACTGAACGCAGCTGGGCACACATGGCAGCTGT
TGCTTCTGCTTACCAGCACATGTATGATCGGTCATTACAGAAGGTTGTGAAGAGTGAAACATCTGGAA
ACTTTGAAGTTGCTCTGATAACTATCCTCAGATGTGCTGAGAATCCAGCTAAGTATTTTGCTAAGGTGTT
AAGGAAGTCCATGAAAGGTCTAGGTACTGATGACAAGACACTTGTAAGGGTTGTGGTAACGAGGACT
GAGATTGATATGCAGTATATCAAGGCAGAATACTACAAGAAATACAAAAAGCCGTTAGCTGATGCTATC
CATTCAGAAACATCAGGAGGTTATCGGACGTTCCTGCTTTCTCTTGTTGGTAGCCATTAG

>HvAnn10 MLOC_54650.4

ATGGCCTCCCGTTCTCCTGCCACCGCAGGGTTCGAAAGCGAGTGCAGAGAGATCCATGGCGCGTGCG
ATGAGCCCCGCCGTCTGATCCGCTACCTGGCTCACCGGAGCGCGCCGGAGAGGCAGCAGATCAAGGC
AACTTACCGCACAATGTTCGGCGAGGACCCCGTCGGCAGGCTTCATAAAACCCTCACGGCCAATCAGG
ACAATGAGCTCTGCAATCTGCTCTACCTGTGGATGCTTGATCTGGCGGAGCGAGACGCGATCATGGCG
AGGGACGCCATCGAGAGCGGCGCGGCGGCCGATTACCGCGCCCTTGTCGAGATCTTCACGCGGCGGA
AGCAGGACCAGCTCTTCTTCACCAAGCAGGCGTACCTGGCTAGGTTCAAGAAGAACCTGGAGCAGGA
CATGGTCACCGACCCCTCGCACCCTTATCAGAGACTGTTGGTAGCTCTGGCAACCTCCCACAAGTCGC
ACCACGATGAACCCAGCAGGCACATCGCGAAATGCGACGCCAGGCGATTGTACGACGCCAAGAACGG
CGCCACGGGGTCGGTCGACGAGGCCGCTATTCTCGAGATGTTCAGCAAGAGGAGCATCCCACAGCTC
AGGCTGGCGCTCTGCAGTTACAAGCACATATATGGGCATGACTTCACCAAAGCACTGAAGAAGAATTC
GGCTGGTGATTTTGTAGAGTCTCTGAAAGTTGTTGTCAAGTGCATCCACAGTCCTTCCAATTATTACTG
CAAGTTACTGCAGAGAAGTATGCAACGCCCAGAAACCAATAAAAGGTTGGTTGCGAGGGCTATCTTG
GGCAGCGATGACGTCGGTGTGAACGAGATCAAGTTAGCGTTCAAGAGTAACTTCGGAAAGAACTTAG
AAGATTTCATCCATGAAAGCTTACCACAGAGTGATTACAGAGACTTTCTTTGGATATGGCAAGGGGGG
CAGTGCCCAGTGGCATCA

>HvAnn11 MLOC_51591.2

ATGGCGACGCTCTCCGTCCCCGCCGCCGTCCCGGCCGTGGCCGAGGACTGCGAGCAGCTCCGCAAGG
CGTTCGCCGGGTGGGGCACCAACGAGCGGCTCATCATCTCCATCCTGGCCCACCGCGACGCCGCGCA
GCGCCGCGCCATCCGCAAGCACTACGCCGACGCCTACGGCGAGGAGCTGCTCCGCTCCATCACCGAC
GAGATCCACGGCAAGTTCGAGAGGGCGGTGATCCAGTGGACGCTGGACCCGGCGGAGCGGGACGCG
GTGCTGGCCAACGAGGAGGCCAAGAAGTGGAACCCGGGGGGCCGCGCGCTCGTCGAGATCGCCTGC

GCGCGCACCCCCGCGCACCTCTTCGCCGCCAAGCAGGCCTACCACGACCGCTTCAAGCGCTCGCTCG
AGGAGGACGTCGCCGCGCACGTCACCGGCGACTTCCGCAAGCTTTTGGTGCCTCTTGTAAGCGCGTA
CCGCTATGATGGCCCGGAGGTGAATACGTCGTTGGCGCACTCTGAAGCCAAGATACTCCATGCGAAGA
TCAGCGAGAAGGCCTACAGTGATGATGAGGTCGTCAGGATTCTCACTACAAGGAGCAAAGCTCAGCT
ACTTGCAACATTCAACAGCTACAATGATCAGTTCAGTCACCCTATCACCAAGGACCTCAAGGAAGATC
CCAAGGACGAGTTCCTCTCGACCCTGAGGGCGATCATAAGGTGTTTCACTTGCCCCGACAGATACTTT
GAGAAGGTCATCCGGTTGGCTCTTGGTGGTGTCGGTACAGACGAGCACACCCTTACTAGGGTGATAAC
CACTCGTGCCGAGGTGGACCTGAAGGTGATAAAGGAGGCGTACCAGAAGAGGAACAGCGTCCCCCT
GGAGAAGGCTGTTTCCAAGGACACAACCAGAGACTATGAAGATATGATGCTTGCACTTCTGGGAGCA
GAGTAC
>HvAnn12 AK248515
ATGGCAACGCTCAAGGTCCCCTCCAACGTCCCCGCCCTCGCCGACGACTGCGACAACCTCCGCAAGG
CCTTCCAAGGGTGGGGCACGAACGAGGCGCTCATCATCTCCATCCTGGGCCACCGCGACGCCGCGCA
GCGCCGCGCCATCCGCAAGCACTACGCCGACACCTACGGCGAGGAGCTGCTCCGGAGCATCACCGAC
GAGATCTCAGGCGACTTCGAGAGGGCCGTGATCCTGTGGACGCTGGACCCCGCGGAGCGGGACGCGG
TGCTCGCCAACGAGACCGCCAAGAAGTGGCACCCGGGGAACCCCGTGCTCGTCGAGATCGCCTGCTC
GCGCGGCTCCGCGCAGCTCTTCGCCGTCAGGCAGGCCTACCATGACCGCTTCAAGCGCTCGCTCGAG
GAGGACGTCGCTGCGCACGTCACCGGCGACTTCCGCAAGCTCTTGGTGCCACTTGTAAGCTCATACCG
CTATGAGGGACCAGAGGTCAACACAAGGTTGGCACATTCAGAGGCCAAAATACTGCATGAGAAGATC
GAGCACAAGGCTTACGGTGATGATGAGATCATCAGAATCCTCACCACCAGGAGCAAAGCTCAGCTGCT
TGCAACACTCAATAACTACAATGACACATTTGGTCACCCAATCACCAAGGATCTGAAGGCTGACCCCA
AGGATGAGTTCCTCAAAACCCTGCGGGCGGTTATCCGGTGCTTCACCTGCCCTGATAGGTACTTTGAG
AAGGTTGCCAGGTTGGCCATTGCAGGGAATGGAACCGATGAGAACTCCCTCACTAGGGTCATAACGA
CCCGCGCCGAGGTGGACCTGAAGCTGATCAAAGAGGCATACCAGAAGAGGAACAGTGTCCCCTTGGA
GAAGGCCGTCGCAGGCGACACCTCCGGCGACTACGAGACCATGCTCCTTGCCCTCCTGGGGAAGGAG
TGA

3). *T.urartu*

>TuAnn1 TRIUR3_22191
MASLSVPPVLTPPRDDAVALHKAFKGFGCDSTTNAMLLWVLDPVGRDATILNQALNGDITDLRAATELLL
AYLGVRRNEGPEVDPSAVTDDARELYQAGEKRVGTDERAFIRIFSERSWAHMVSVANAYQHMYARSLEKA
VKSETTGNFQFGLLTILRCADTPAKYFAKVLHKAMKGLGTSNAALTRVAVTRTEVDMKYIKAEYHNKVIM
EGTDKEAEHPVPIYTDLGLSVPARTRSVAAAKAGRGSQWREGGEVEVRHGRGVAEASHGTGTAFVFALVE
WEWRE
>TuAnn2 TRIUR3_11010
MASISVPDPAPSPTEDAESIRKAVQGWGTDENALIEILGHRTAAQRAEIAVAYEGLYDKPLLRTLQDELSSHF
KGAMTLWAMDPAARDAKLAYKALRKKGGDRHAWVLIEVACASSPDHLVAVRKAYCSAYDSSLEEDVAA
CPLYKDPLKQARPCRSWHSPSFVAAVGPFLALAVAVLGAPGELAAELHGAVAAKKQPLHGDVVRIVSSRS
KPQLKATFQHYKREHGKAIDEVLEGNRNDKLSAMLKTAVWCLTSPEKHFAEVIRSSIIGLGTDEESLTRAIV
SRAEVDLKKVKEEYKVRYKTTVTKDVVGDTSGYYQGILLTLIGAE
>TuAnn3 TRIUR3_11009
MADEVQALTKAFSGLGGLGVDEPTMVAALANWRRQPEKRSGFRRSFPGLFKEQGVIERCEDEYMLRLAA
EFSRFKNLMVLWAMHPWERDARLAHHVLHQAHPAAILVEVACTRSAEELLGARKAYMALFHHSLEEDVA
YRAKDKPYCSLLVGLVSAYRYEGPKVSDDTAKAEAKALGAAVKGAAGGRLVENEEVVRILTTRSKPHLV

QTFKYYKELHGKHIQEDLGSEEALREAVRCLATPERYFSQVMAAALREGADHHGKEALARVAVTRSDVD
MDAIRAAYQEQFGAKLEDAVAGSAHGHFKDALVSLIVGK
>TuAnn6 TRIUR3_18622
MAEHTRISLRFLSTSLLAIVVCCTPGLLWLRRLVARTGPAWRRRMASLSVPPVLTSPRHDAIALHRAFKGFG
CDSTTVINILAHRDAKAMLLWVLDPVGRDATILNQSLNGDITDLRAATEVICSRTPSQLQIMKQAYRARFG
CYLEQDVTERTYGDHQKLLLAYLGIPRWAHLAAVADAYHHLYARSLDKAVKSETSGTFELGLLTILRCAES
PARYFAKALHKAMKGLGTSDTTRGGVIRVGVTRAEVDMQYIKAEYHKKYKRSLADAIHSETSGNYRTFL
LSLVGRDR
>TuAnn9 TRIUR3_32496
MASLTLPPAPPNPRQDAIDLHKAFKGFGCDTTTVSNILAHRDSMQRGYIQQEYKTMYSEELSRRISSELSG
NHKVWKIVEKSLLHDKSNYCTAILAEEKALSLWILDPAGRDATVLKEALSAESLDLKAATDIICSRTPSQLQ
IMKQTYYAKFGTYLEHDISQQTSGDHQKILLAYVGIPRYEGPEVDPTIVTHDAKDLYKAGEKKLGTDEKTF
IRIFTERSWAHMAAVASAYHHMYDRSLQKVVKNETSGNFEVALLTILRCAENPAKYFAKVLRKSMKGLGT
DDKTLVRVVVTRTEIDMQYIKAEYYKKYKKPLADAIHSETSGGYRTFLLSLVGSH
>TuAnn10 TRIUR3_04012
MAARSPAAAGFENECREIHGACDEPRRLSRLLAHRSASERQQIKATYRAMFGEDLAGRLHKTLTANQDNE
LCNLLYLWMLDLAERDAIMARDAIESGAAADYRALVEVFTRRKQDQLFFTKQAYLSRFKKNLEQDMDTD
PSHPYQRQLLVALATSHKSHHDEPSRHIAKCDARRLYDAKNGGGTGSVDEAAILEMFSKRSIPQLRLALCS
YKHIYGHDFTKALKKNLAGDFEESLRLVVKCIYSPSKYYCKLLQRSMQRPEAHKRLVTRAILGSDDVGIN
EIKLAFKSNFGKNFANFIHESLPQSDYREFLWLWQGGRCPVAS
>TuAnn11 TRIUR3_16577
MATLSVPAAVPAVAEDCEQLRKAFAGWGTNERLIVSILAHRDAAQRRAIRRAYAEAYGEELLRAIGDEIHG
KAVIQWTLDPAERDAELANEEAKKWQPGGRALVEIACARTPAQLFAARQAYHDRFKRSLEEDVAAHVTG
DFRKLLVPLVSAYRYDGPEVNTSLAHSEAKLLNGKINEKAYSDDEVVRILTTRSKAQLLATFNSYNDQFSH
PITKDLKEDPKNEYLSTLRAIIRCFTCPDRYFEKVVRLALGGVGTDEDTLTRVITTRAEVDLKVIKEAYQKR
NSVPLEKAVSKETSRDYEDMMLALLGAEY
>TuAnn12 TRIUR3_03306
MATLKVPSNVPALADDCDNLRKAFQGWGTNEALIISILGHRDASQRRAIRKHYADTYGEELLRSITDEISG
DFERAVILWTLDPAERDAVLANETARKWHPGNPVLVEIACARGSKQLFAVRQAYHDRFKRSLEEDVASHV
TGDFRKLLVPLVSSHRYEGPEVNTRLAHSEAKLLHEKIEHKAYADDEVIRILTTRSKAQLLATFNNYNDTFG
HPITKDLKADPKDEFLKTLRAVIRCFTCPDRYFEKVARVAIAGNGTDENSLTRVITTRAEVDLKLIKEAYQK
RNSVPLEKAVAGDTSGDYESMLLALLGKE
>TuAnn1 TRIUR3_22191
ATGGCGAGCCTGAGCGTGCCTCCGGTGCTCACGCCTCCGCGTGATGACGCCGTCGCTCTCCACAAGGC
CTTCAAAGGGTTCGGCTGCGACAGCACGACGAATGCTATGCTGCTTTGGGTCCTCGACCCCGTGGGGC
GTGACGCGACCATACTGAACCAGGCTCTCAACGGCGACATCACCGACCTGAGAGCCGCCACGGAGCT
TTTGCTTGCGTACCTGGGAGTCCGGCGCAACGAAGGCCCGGAAGTTGATCCTTCGGCGGTGACAGAC
GACGCGAGGGAGCTGTATCAAGCCGGCGAGAAGAGGGTGGGCACCGATGAGCGGGCCTTCATCCGCA
TCTTCAGCGAACGCAGCTGGGCGCACATGGTGTCCGTGGCCAACGCTTACCAGCACATGTACGCCCGG
TCCCTGGAGAAGGCCGTGAAGAGTGAGACGACAGGGAACTTCCAGTTCGGGCTGCTGACCATCCTCA
GGTGCGCCGACACTCCGGCAAAGTACTTTGCCAAGGTCCTGCACAAGGCGATGAAAGGCCTGGGCAC
CAGCAACGCGGCGCTCACACGGGTGGCGGTGACGAGGACCGAGGTTGACATGAAGTACATCAAGGC
AGAGTACCACAACAAGGTGATCATGGAGGGCACCGACAAGGAAGCAGAGCATCCTGTGCCAATCTAC
ACCGACCTAGGGCTGAGCGTCCCGGCGCGGACGCGCTCCGTGGCCGCGGCAAAGGCGGGTCGGGGG
TCCCAGTGGCGTGAAGGCGGCGAGGTGGAAGTGCGGCATGGGCGTGGTGTAGCAGAGGCGAGCCAT

GGGACTGGCACGGCCTTCGTGTTCGCGCTGGTGGAGTGGGAGTGGCGAGAG

>TuAnn1 TRIUR3_11010

ATGGCCTCCATCTCGGTCCCGGACCCCGCCCCTTCCCCGACCGAGGATGC
AGAGAGCATCAGAAAAGCAGTGCAAGGGTGGGGGACGGACGAGAACGCGCTGATCGAGATCCTGGG
CCACCGGACGGCGGCGCAGCGCGCGGAGATCGCCGTCGCCTACGAGGGCCTCTACGACAAGCCCCTC
CTCCGCACGCTCCAGGACGAGCTCTCCAGCCACTTCAAGGGCGCGATGACGCTGTGGGCGATGGACC
CGGCGGCGCGGGACGCCAAGCTGGCCTACAAGGCCCTCAGGAAGAAGGGCGGCGACCGGCACGCCT
GGGTGCTCATCGAGGTCGCCTGCGCGTCGTCGCCGGACCACCTCGTCGCCGTCAGGAAGGCCTACTG
CTCCGCCTACGACTCGTCGCTCGAGGAGGACGTCGCCGCCTGCCCGCTCTACAAGGACCCCCTCAAG
CAGGCAAGGCCGTGCCGATCCTGGCATTCTCCTTCTTTCGTCGCCGCCGTGGGTCCATTTCTTGCTCTT
GCCGTTGCCGTTCTTGGTGCGCCTGGTGAGCTCGCGGCCGAGCTGCACGGCGCGGTGGCGGCCAAGA
AGCAGCCGCTGCACGGGGACGTGGTCCGCATCGTCAGCTCCAGGAGCAAGCCGCAGCTCAAGGCGA
CGTTCCAGCACTACAAGCGAGAGCACGGCAAGGCCATCGACGAGGTTCTCGAGGGCAATCGCAACGA
CAAGCTGTCGGCGATGCTGAAAACTGCGGTCTGGTGCCTGACATCGCCGGAGAAGCACTTCGCAGAG
GTGATCCGGAGCTCGATCATCGGGCTCGGCACCGACGAGGAATCCCTGACCAGAGCGATTGTCTCGCG
CGCCGAGGTCGACTTGAAGAAAGTGAAGGAGGAATACAAGGTGAGGTACAAGACGACGGTGACCAA
GGATGTCGTCGGAGACACCTCCGGGTACTACCAGGGCATCCTGCTCACCCTCATCGGGGCCGAGTAG

>TuAnn3 TRIUR3_11009

ATGGCCGACGAAGTTCAGGCGCTCACCAAGGCCTTCTCAGGTCTGGGAGGCCTGGGCGTGGACGAGC
CGACGATGGTGGCGGCGCTGGCGAACTGGCGGAGGCAGCCGGAGAAGCGGTCCGGGTTCCGGAGGA
GCTTCCCGGGGCTGTTCAAGGAGCAGGGCGTGATCGAGCGGTGCGAGGACGAGTACATGCTGCGCCT
GGCCGCCGAGTTCTCCCGCTTCAAGAACCTCATGGTGCTGTGGGCGATGCACCCGTGGGAGCGCGAC
GCCCGCCTCGCGCACCACGTCCTCCACCAGGCCCACCCGGCCGCCATCCTCGTGGAGGTCGCCTGCA
CCCGCTCCGCCGAGGAGCTCCTCGGCGCGCGCAAGGCCTACATGGCGCTCTTCCACCACTCCCTCGAG
GAGGACGTCGCCTACCGCGCCAAGGACAAGCCCTACTGCAGCCTGCTGGTGGGGCTGGTGAGCGCGT
ACCGGTACGAAGGGCCCAAGGTGAGCGACGACACGGCCAAGGCGGAGGCCAAGGCGCTGGGCGCG
GCGGTGAAGGGCGCCGCCGGCGGCAGGCTGGTGGAGAACGAGGAGGTGGTCAGGATCCTCACCACC
AGGAGCAAGCCGCACCTCGTCCAGACCTTCAAGTACTACAAGGAGCTGCACGGCAAGCACATCCAGG
AGGATCTGGGAAGCGAGGAGGCTCTGAGGGAGGCCGTGCGGTGCCTGGCGACGCCGGAGAGGTACT
TCAGCCAGGTGATGGCGGCGGCGCTGAGGGAGGGCGCGGACCACCACGGCAAGGAGGCCCTGGCGC
GGGTGGCGGTGACGCGGTCGGACGTGGACATGGACGCCATCCGGGCGGCCTACCAGGAGCAGTTCGG
GGCCAAGCTCGAGGACGCCGTCGCCGGCAGCGCGCACGGCCACTTCAAGGACGCGCTCGTCTCCCTC
ATCGTCGGCAAGTAA

>TuAnn6 TRIUR3_18622

ATGGCGGAGCACACCCGCATCTCCCTGAGATTCCTGAGCACGTCGCTGCTAGCCATCGTGGTGTGCTG
CACGCCGGGGTTGCTGTGGCTCCGGCGCCTGGTGGCACGCACCGGCCCCGCGTGGAGGCGAAGAATG
GCGAGCCTGAGCGTGCCTCCGGTGCTCACGTCCCCGCGCCATGACGCCATCGCCCTGCACAGGGCCTT
CAAAGGTTTTGGGTGCGACAGCACGACGGTGATCAACATCCTGGCGCACCGCGACGCGAAGGCGATG
CTGCTGTGGGTGCTGGACCCCGTGGGCCGCGACGCCACGATACTGAACCAGTCCCTCAACGGCGACA
TCACCGACCTGCGCGCCGCCACGGAGGTGATCTGCTCCCGGACGCCGTCGCAGCTGCAGATCATGAA
GCAGGCCTACCGCGCCCGCTTCGGTTGCTACCTCGAGCAGGACGTCACCGAGCGCACCTACGGCGAC
CACCAGAAGCTTCTGCTGGCCTACCTGGGCATCCCGCGCTGGGCGCACCTCGCCGCCGTCGCCGACG
CCTACCACCACCTCTACGCGCGCTCCCTGGACAAGGCTGTGAAGAGCGAGACGTCGGGCACCTTCGA
GTTGGGGCTGCTGACGATCCTCCGGTGCGCCGAGAGCCCGGCGAGGTACTTCGCCAAGGCGCTGCAC
AAGGCGATGAAGGGGCTGGGCACAAGCGACACGACGCGGGGAGGCGTGATACGGGTGGGGGTCACC

AGGGCGGAGGTGGACATGCAGTACATCAAGGCCGAGTACCACAAGAAGTACAAGCGCTCGCTGGCCG
ACGCCATCCACTCCGAGACCTCCGGCAACTACCGGACCTTCCTCCTCTCCCTCGTCGGCCGCGACCGC
TAA
>TuAnn9 TRIUR3_32496
ATGGCGAGCCTCACCTTGCCGCCGGCGCCCCCTAACCCCCGCCAGGACGCCATCGACCTCCACAAGG
CCTTCAAAGGGTTTGGCTGTGATACTACAACAGTTTCAAATATACTTGCTCACCGTGACTCAATGCAAC
GTGGATACATTCAACAGGAATACAAAACTATGTATTCTGAGGAACTTTCACGTCGTATATCGTCTGAAC
TCAGTGGAAACCACAAGGTATGGAAAATTGTAGAAAAATCTTTATTGCATGATAAATCAAACTATTGCA
CAGCTATACTGGCTGAAGAGAAAGCACTGTCGCTCTGGATTCTTGATCCTGCTGGACGTGATGCGACT
GTTCTGAAAGAAGCTCTAAGCGCTGAAAGTCTTGATCTGAAAGCAGCTACTGATATAATTTGTTCCAG
GACACCATCACAGCTGCAAATAATGAAACAGACATATTATGCAAAGTTTGGTACTTATCTTGAGCATGA
CATTAGTCAGCAAACATCCGGCGATCATCAGAAGATCTTACTAGCCTATGTTGGCATTCCACGCTACGA
AGGCCCGGAGGTTGATCCCACTATAGTGACACATGATGCGAAGGACCTCTACAAAGCTGGTGAGAAA
AAGCTGGGCACAGATGAGAAAACCTTCATCCGCATCTTCACTGAACGCAGCTGGGCACACATGGCAG
CTGTTGCTTCTGCTTACCATCACATGTATGATCGGTCGTTACAGAAGGTTGTGAAGAATGAAACATCTG
GAAACTTTGAAGTTGCTCTGTTAACTATCCTCAGATGTGCTGAGAATCCAGCGAAGTATTTTGCTAAGG
TGTTGAGGAAGTCCATGAAAGGTCTAGGTACTGATGACAAGACACTTGTAAGGGTTGTGGTGACAAG
GACTGAGATTGATATGCAGTATATCAAGGCAGAATATTACAAGAAATACAAAAAGCCGTTAGCTGATGC
TATCCATTCCGAAACATCAGGCGGTTATCGGACGTTCCTGCTTTCTCTTGTTGGTAGCCATTAG
>TuAnn10 TRIUR3_04012
ATGGCCGCCCGTTCTCCTGCCGCCGCAGGGTTCGAAAACGAGTGCAGAGAGATCCATGGCGCGTGCG
ACGAGCCGCGCCGCCTGAGCCGCCTCCTGGCTCACCGGAGCGCGTCGGAGAGGCAGCAGATCAAGG
CGACGTACCGTGCAATGTTCGGCGAGGACCTCGCCGGCAGGCTCCACAAAACCCTCACGGCCAATCA
GGACAATGAGCTCTGCAACCTGCTCTACCTGTGGATGCTTGACCTGGCGGAGCGAGACGCGATCATGG
CGAGGGACGCCATCGAGAGCGGCGCGGCGGCCGATTACCGCGCCCTTGTCGAGGTCTTCACGCGGCG
GAAGCAGGACCAGCTCTTCTTCACCAAGCAGGCGTACCTGTCCAGGTTCAAGAAGAACCTGGAGCAG
GACATGGACACCGACCCCTCGCACCCTTATCAGAGACAGCTATTGGTAGCTCTGGCAACCTCCCACAA
GTCGCACCACGATGAACCCAGCCGGCACATCGCGAAGTGCGACGCCAGGCGATTGTACGACGCCAAG
AACGGTGGCGGCACGGGATCGGTCGACGAGGCCGCCATCCTCGAGATGTTCAGCAAGAGGAGCATCC
CACAGCTCAGGCTGGCGCTCTGCAGTTACAAGCATATATATGGGCATGACTTCACCAAAGCACTGAAG
AAGAATTTGGCTGGTGATTTTGAAGAGTCTCTGAGACTTGTTGTCAAGTGCATCTACAGTCCTTCCAA
GTATTACTGCAAGTTACTGCAGAGAAGCATGCAACGCCCAGAAGCCCATAAAAGGTTGGTTACGAGG
GCTATCTTGGGCAGCGACGACGTCGGCATAAACGAGATCAAGTTGGCGTTCAAGAGTAACTTCGGAA
AGAACTTCGCAAATTTCATCCATGAAAGCTTACCACAGAGTGATTACAGAGAGTTTCTTTGGTTGTGG
CAAGGGGGCCGGTGCCCAGTAGCATCATAA
>TuAnn11 TRIUR3_16577
ATGGCGACGCTCTCCGTCCCCGCCGCCGTCCCGGCCGTCGCCGAGGACTGCGAGCAGCTGCGCAAGG
CGTTCGCGGGGTGGGGCACCAACGAGCGCCTCATCGTCTCCATCCTGGCGCACCGCGACGCCGCGCA
GCGCCGCGCCATCCGCCGCGCCTACGCCGAGGCCTACGGCGAGGAGCTGCTCCGCGCCATCGGCGAC
GAGATCCACGGCAAGGCGGTGATCCAGTGGACGCTGGACCCGGCGGAGCGGGACGCGGAGCTGGCC
AACGAGGAGGCCAAGAAGTGGCAGCCGGGGGGCCGCGCGCTCGTCGAGATCGCCTGCGCGCGCACC
CCCGCGCAGCTCTTCGCCGCTAGGCAGGCCTACCACGACCGCTTCAAGCGCTCGCTCGAGGAGGACG
TCGCCGCGCACGTCACCGGCGACTTCCGCAAGCTTTTGGTGCCTCTTGTAAGCGCATACCGCTACGAT
GGCCCGGAGGTGAACACATCGTTGGCACACTCTGAAGCCAAATTACTCAACGGAAAGATCAACGAGA
AGGCCTACAGCGATGATGAGGTCGTCAGGATTCTCACTACAAGGAGCAAGGCTCAGCTACTCGCAACA

TTCAACAGCTACAATGATCAGTTCAGTCACCCCATCACCAAGGACCTCAAGGAAGACCCCAAGAACG
AGTACCTCTCAACCCTGAGGGCGATCATAAGGTGTTTCACCTGCCCTGACAGATACTTTGAGAAGGTC
GTCCGGTTGGCCCTTGGAGGCGTCGGCACAGACGAGGACACCCTTACGAGGGTCATAACCACTCGCG
CTGAGGTGGACCTGAAGGTGATAAAGGAGGCGTACCAGAAGAGGAACAGCGTCCCGCTGGAGAAGG
CTGTTTCCAAGGAAACAAGCAGAGACTATGAAGATATGATGCTTGCACTTCTGGGGGCAGAGTACTGA
>TuAnn12 TRIUR3_03306
ATGGCGACGCTCAAGGTCCCCTCCAACGTCCCGGCCCTCGCCGACGACTGCGACAACCTCCGCAAGG
CCTTCCAAGGGTGGGGCACGAACGAGGCGCTCATCATCTCCATCCTCGGCCACCGCGACGCGTCGCA
GCGCCGCGCCATCCGCAAGCACTACGCCGACACCTACGGCGAGGAGCTGCTCCGGAGCATCACCGAC
GAGATCTCCGGCGACTTCGAGAGGGCCGTGATCCTGTGGACGCTGGACCCGGCGGAGCGGGACGCGG
TGCTCGCCAACGAGACCGCCAGGAAGTGGCACCCCGGGAACCCCGTGCTGGTCGAGATCGCCTGCGC
GCGCGGCTCCAAGCAGCTCTTCGCCGTCAGGCAGGCCTACCATGACCGCTTCAAGCGCTCGCTCGAG
GAGGACGTCGCTTCCCACGTCACCGGCGACTTCCGCAAGCTGTTGGTGCCACTTGTAAGCTCACACC
GCTACGAGGGACCAGAGGTCAACACAAGGTTGGCTCATTCAGAAGCCAAATTACTACACGAGAAGAT
TGAGCATAAGGCTTATGCTGATGATGAGGTCATCAGAATTCTCACCACCAGGAGCAAAGCTCAGCTGC
TTGCAACATTCAATAATTACAATGATACATTTGGTCACCCGATCACTAAGGATCTGAAGGCTGACCCCA
AGGATGAGTTCCTCAAGACCCTGCGGGCGGTCATCCGGTGCTTCACCTGCCCTGATAGGTACTTTGAG
AAGGTGGCCAGGGTGGCCATTGCAGGGAATGGAACCGATGAGAACTCCCTCACTAGGGTCATCACCA
CCCGCGCCGAGGTGGACCTGAAGCTGATCAAAGAGGCGTACCAGAAGAGGAACAGTGTTCCCCTGG
AGAAGGCCGTCGCAGGCGACACCTCCGGCGACTACGAGAGCATGCTCCTTGCCCTCCTGGGGAAGGA
GTGA

4). *A.tauschii*

>AeAnn1 AEGTA07604
MASLSVPPVLTPPRDDAVALHKAFKGFGCDSTTQNAMLLWVLDPAGRDATILNQALNGDITDLRTATEVIC
SRTPSQLQIMKQTYRARFGCYLEHDITERTYGDHQKILLAYLGVPRNEGPEVDPSAVTNDARELYRAGEKR
VGTDERAFIRIFTESSRAHLVSIANAYQHMYARSLEKTLKSETTGNFQFGLLTILRCADTPAKYFAKVLHKA
MKGLGTSNAALIRVAVTRTEVDMKYIKAEYHNKYKGSLAEAIHSETSGNYRTFLLSLVGRDR
>AeAnn2 AEGTA15612
MASISVPDPVPSPTEDAESIRKAVQGWGTDENALIEILGHRTAAQRAEIAVAYEGLYDKPLLRTLQDELSSH
FKGAMTLWAMDPAARDAKLAYKALRKKGGDRHAWVLIEVACASSPDHLVAVRKAYCSAYGSSLEEDVA
ACPLYKEPLKQARPCGSWHSPLFFWSPPWVHFLLLPLLFLVRLVSSYRYGGEHVDGELARAEAADLHGAV
AAKKQPLHGDVVRIVSSRSKPQLKATFQHYKQEHGKAIDEVLEGNRNDKLSAMLKTAVWCLTSPEKHFA
EVIRSSIIGLGTDEESLTRAIVSRAEVDMKKVKEEYKVRYKTTVTKDVVGDTSGYYQGILLTLIGAE
>AeAnn3 AEGTA29052
MADEVQALTKAFSGLGGLGVDEPTMVAALANWRRQPEKRSGFRKSFPGLFKQHGVIERCEDEYMLHLA
AEFSRFKNLMVLWAMHPWERDARLAHHVLHQAHPAAIVVEVACTRSAEELLGARKAYMALFHHSLEED
VAYRAKDKPYCSLLVGLVSAYRYEGPKVSDDTAKAEAKALGAAVKSAGGGKLVENEEVVRILTTRSKPHL
VQTFKYYKELHGKYIEEDLGSEEALREAVQCLATPERYFSQVMAAALREGADHHGKEALARVAVTRSDV
DMDGIRAAYQEQFGAKLEDAVAGSAHGHFKDALVSLIVGK
>AeAnn4 AEGTA21287 (genebank: EMT22984.1)
MATITVPQVVPSPVEDAEALMKAFQGWGTDEQAVIYILAHRDAAQRKLIRLAYEEKYDERLTDRLESELS
GDLQTAMGYWVLDPTERQAVIANAATKCIDEEYPVIVEIACANSPAELLEVKRAYHAIYKRSLEEDVAAGA
TGNLRNLLVALVSAYRYDGDEVNGGLARSEAKIIQADVKNGVTADHGELIRVLGTRSRAQLGATFNCFRD

EHGTTVTKALLHAPDTTGYARALRTAARCIADTDKYFAKVLRNAMHKSGTDEESLLRVVVMRAEKDLR
GIKDEFHKRASVALEQAIAKETSGDFKTFITALVGTSQ
>AeAnn5 AEGTA42363
MPTGTAAAEAGHGTPYVDPEDGEEGEWGARWRADKEALVRILARRTAAQRSAIRRAYAFLFREPLLNSFR
QRLSRQYCPVTVDFWVRQRTHASLRRRSSCGRWTRRSGTRTWCTARCGGEGTATTSPCWWRSRARPDPT
PAGXAYRSLFGCSVEEDLASCAALQQPLRKMLVSLVSSYRYGGDRVDADVAKLEASQLSEAVRRKQPHH
DEVVRILSTRSKPQLRATLRRYREDHGTDIVEDIDSRCSSQFARTLKSAVWCLTSPEKHFAEMIRESVVGLG
TYEDMLTRVVVSRAEIDMRQIKEEYRARFKTTVTCDVVDDTSFGYKDILLALVGGEEE
>AeAnn6 AEGTA16509
MASLSVPPVLTSPRHDAIALHRAFKGFGCDSTTVINILAHRDAKAMLLWVLDPVGRDAAILNQSLNGDIT
DLRAATEVICSRTPSQLQIMKQTYRARFGCYLEQDITERTYGDHHKLLLAYLGIPRAEVDMQYIKAEYHK
KYKRSLADAIHSETSGNYRTFVLSLVGRDR
>AeAnn7 AEGTA11977
MPCMVTCVHDEGGGADEKALVEILGRRTAAQRAEIRRAYASLYKESLLTRLHGELSGHFQKAMVLLATDP
AERDAKLAREALGRRGDDRDAWVLIEAACAAAPDHLVAVRRAYRSLHGSSLEEDVAACSAFQEPLRKVT
TPGEEQSNPFQSILLVSLVRSYRCAEESVDMDVAKLEAAQLAEAIRRKKQPHAGEVVRIVSTRSKPQLAAT
FRCYKEQHGSDIEEDMKQYSSSQFARMLKIAVWCLTSPEKHFAEVIRYSILGLGTDEDALTRAIVSRADIDM
KKIKQEYRVRFKTTVTDDVVGDTSGYYMDILLALVGKE
>AeAnn9 AEGTA10144
MASLTLPPAPPNPRQDAIDLHKAFKGFGCDSTTVSNILSHRDSMQRGYIQQEYKTMYSEELSHRISSELSGN
HKVWKIVEKSLLHDKSNYCTAILAEEKALSLWILDPAGRDATVLKEALSAESLDLKAATDIICSRTPSQLQI
MKQTYYAKFGTYLEHDISQQASGDHQKILLAYVGIPRYEGPEVDPTIVTHDAKDLYKAGEKKLGTDEKTFI
RIFTERSWAHMAAVASAYRHMYDRSLQKVVKNETSGNFEVALLTILRCAENPAKYFAKVLRKSMKGLGT
DDKTLVRVVVTRTEIDMQYIKAEYYKKYKKPLADAIHSETSGGYRTFLLSLVGSH
>AeAnn10 AEGTA28110
MAARSPAAAGFENECREIHGACDEPRRLSRLLAHRSASERQQIKATYRAMFGEDLAGRLHKTLTANQDNE
SQHGLPLQLCNLLYLWMLDLAERDAIMARDAIESGAAADYRALVEVFTRRKQDQLFFTKQAYLARFKKN
LEQDMVTDPSHPYQRLLVALATSHKSHHDEPSRHIAKCDARRLYDAKNGGGTGSVDEAAILEMFSKRSIP
QLRLALCSYKHIYGHDFTKALKKNLAGDFEESLRVVVKCIYTPSKYYCKLLQRSMQRPEADKRLVTRAIL
GSDDVGINEIKLAFKSNFGKNLADFIHESLPQSDYREFLWLWQGGQCLVAS
>AeAnn11 AEGTA31729
MATLSVPAAVPAVAEDCEQLRKAFAGWGTNERLIVSILAHRDAAQRRAIRRAYAEAYGEELLRAIGDEIHG
KFERAVTQWTLDPAERDAELAGEEAKKWQPGGRALVEIACARTPAQLFAAKQAYHDRFKRSLEEDVAAH
VTGDFRKLLVPLVSAYRYDGPEVNTSLAHSEAKILNGKINEKAYSDDEIVRILTTRSKAQLLATFNSYNDQF
SHPITKDLKEDPKNEFLSTLRAIIRCFTCPDRYFEKVVRLALGGVGTDENTLTRVITTRAEVDLKVIKEAYQ
KRNSVPLEKAVSKETSRDYEDMMLALLGAEY
>AeAnn12 AEGTA27331
MATLKVPSNDPALADDCDNLRKAFQGWGTNEALIISILGHRDAAQRRAIRKHYADTYGEELLRSITDEISG
DFERAVILWTLDPAERDAVLANETAKKWHPGNPVLVEIACARGSKQLFAARQAYHDRFKRSLEEDVAAHV
TGDFRKYLIPLGYIQIKFSCDGGFCYLVPGHFLILELLVPLVSSHRYEGPEVNTRLAHSEAKLLHEKIEHKAY
GDDEVIRILTTRSKAQLLATFNNYNDTFGHPITKDLKADPKDEFLKTLRAVIRCFTCPDRYFEKVARLAIAG
NGTDENSLTRVITTRAEVDLKLIKEAYQKRNSVPLEKAVAGDTSGDYESMLLALLGKE
>AeAnn1 AEGTA07604
ATGGCGAGCCTGAGCGTGCCTCCGGTGCTCACGCCCCCGCGCGATGACGCCGTCGCTCTCCACAAGG
CCTTCAAAGGTTTCGGCTGCGACAGCACGACGCAGAATGCTATGCTGCTTTGGGTCCTCGACCCTGCT

GGGCGTGATGCGACCATACTGAACCAAGCTCTCAACGGCGACATCACCGACCTGAGAACCGCCACCG
AGGTTATCTGCTCCAGGACGCCGTCGCAGCTGCAGATCATGAAGCAAACCTACCGTGCGAGGTTCGGT
TGCTACCTCGAGCATGACATCACCGAACGCACCTATGGAGATCATCAGAAGATTTTGCTTGCGTACCTG
GGAGTCCCACGCAACGAAGGCCCGGAAGTTGATCCTTCGGCGGTGACAAACGACGCGAGGGAGCTG
TATAGGGCCGGCGAGAAGAGGGTGGGCACCGATGAGCGGGCCTTCATCCGCATCTTCACCGAAAGCA
GCAGGGCACACCTGGTGTCCATCGCCAACGCTTACCAGCACATGTACGCCCGGTCCCTGGAGAAGAC
CCTGAAGAGTGAGACGACAGGGAACTTCCAATTCGGGCTGCTGACCATCCTCAGGTGCGCCGACACT
CCGGCAAAGTACTTTGCCAAGGTCCTGCACAAGGCGATGAAAGGCCTGGGCACCAGCAACGCGGCG
CTCATAAGGGTGGCGGTGACGAGGACCGAGGTCGACATGAAGTACATCAAGGCAGAGTACCACAACA
AGTACAAGGGGTCGCTGGCTGAAGCTATCCACTCCGAGACGTCAGGGAACTACCGAACCTTCCTCCTC
TCGCTCGTCGGCCGGGACCGCTAA
>AeAnn2 AEGTA15612
ATGGCCTCCATCTCGGTCCCGGACCCCGTCCCTTCCCCAACCGAGGACGCAGAGAGCATAAGAAAAG
CAGTACAAGGGTGGGGGACGGACGAGAACGCGCTGATCGAGATCCTCGGCCACCGGACGGCGGCGC
AGCGCGCGGAGATCGCCGTGGCCTACGAGGGCCTCTACGACAAGCCCCTCCTCCGCACGCTCCAAGA
CGAGCTCTCCAGCCACTTCAAGGGCGCGATGACGCTGTGGGCGATGGACCCGGCGGCGCGGGACGCC
AAGCTGGCCTACAAGGCCCTCAGGAAGAAGGGCGGGGACCGGCACGCCTGGGTGCTCATCGAGGTC
GCCTGCGCGTCGTCGCCGGACCACCTCGTCGCCGTCAGGAAGGCCTACTGCTCCGCCTACGGCTCGTC
GCTCGAGGAGGACGTCGCCGCCTGCCCGCTCTACAAGGAGCCCCTCAAGCAGGCAAGGCCGTGCGGA
TCCTGGCATTCTCCTCTCTTTTTTTGGTCGCCGCCGTGGGTTCATTTCTTGCTGTTGCCGTTGCTGTTCT
TGGTGCGCCTGGTGAGCTCGTACCGCTACGGCGGCGAGCACGTCGATGGCGAGCTGGCGAGGGCGGA
GGCGGCCGATCTGCACGGCGCGGTGGCGGCCAAGAAGCAGCCGCTGCACGGCGACGTGGTCCGCATC
GTCAGCTCCAGGAGCAAGCCGCAGCTCAAGGCGACGTTCCAGCACTACAAGCAGGAGCACGGCAAG
GCCATCGACGAGGTTCTCGAGGGCAATCGCAACGACAAGCTCTCCGCGATGCTGAAAACCGCGGTCT
GGTGCCTGACATCGCCCGAGAAGCATTTCGCAGAGGTGATCCGGAGCTCAATCATCGGGCTCGGCACC
GACGAGGAGTCCCTGACCAGAGCGATCGTCTCGCGCGCCGAGGTCGACATGAAGAAAGTCAAGGAG
GAATACAAGGTGAGGTACAAGACGACGGTGACCAAGGACGTCGTCGGCGACACTTCCGGGTACTACC
AGGGCATCCTGCTCACCCTCATCGGGGCCGAGTAG
>AeAnn3 AEGTA29052
ATGGCCGACGAAGTTCAGGCTCTCACCAAGGCCTTCTCAGGTCTGGGAGGCCTGGGCGTGGACGAGC
CGACGATGGTGGCAGCGCTGGCGAACTGGCGGAGGCAGCCGGAGAAGCGGTCCGGGTTCCGGAAGA
GCTTCCCGGGGCTGTTCAAGCAGCACGGGGTGATCGAGCGGTGCGAGGACGAGTACATGCTGCACCT
GGCCGCCGAGTTCTCCCGCTTCAAGAACCTCATGGTGCTGTGGGCGATGCACCCGTGGGAGCGCGAC
GCCCGCCTCGCGCACCACGTCCTCCACCAGGCCCACCCGGCCGCCATCGTCGTGGAGGTCGCCTGCA
CCCGCTCCGCCGAGGAGCTCCTCGGCGCGCGCAAGGCCTACATGGCGCTCTTCCACCACTCCCTCGAG
GAGGACGTCGCCTACCGCGCCAAGGACAAGCCATACTGCAGCCTGCTGGTGGGGCTGGTGAGCGCGT
ACCGGTACGAAGGGCCCAAGGTGAGCGACGACACGGCCAAGGCGGAGGCCAAGGCGCTGGGCGCG
GCGGTGAAGAGCGCCGGCGGCGGCAAGCTGGTGGAGAACGAGGAGGTGGTCAGGATCCTCACCACC
AGGAGCAAGCCTCACCTCGTCCAGACCTTCAAGTACTACAAGGAGCTGCACGGCAAGTACATCGAGG
AGGATCTCGGAAGCGAGGAGGCTCTGAGGGAGGCCGTGCAGTGCCTGGCGACGCCGGAGAGGTACT
TCAGCCAGGTGATGGCGGCGGCGCTGAGGGAGGGCGCGGACCACCACGGGAAGGAGGCCCTGGCGC
GGGTGGCGGTGACGCGGTCGGACGTGGACATGGACGGCATCCGGGCGGCCTACCAGGAGCAGTTCGG
GGCCAAGCTCGAGGACGCCGTCGCCGGCAGCGCGCACGGGCACTTCAAGGACGCGCTCGTCTCGCTC
ATCGTCGGCAAGTAA
>AeAnn4 AEGTA21287

ATGGCCACGATCACAGTTCCTCAAGTTGTCCCTTCTCCGGTTGAAGATGCTGAGGCGCTCATGAAGGC
CTTCCAAGGATGGGGCACTGACGAGCAGGCAGTGATCTACATACTAGCTCACCGGGACGCGGCGCAG
AGGAAGCTGATCCGGCTGGCGTACGAAGAGAAATACGACGAGCGCCTCACCGACCGGCTCGAATCAG
AGCTCTCCGGCGATCTTCAGACGGCGATGGGCTACTGGGTACTCGACCCCACGGAGAGGCAGGCCGT
GATCGCCAACGCCGCCACGAAATGCATCGACGAGGAGTACCCCGTGATCGTGGAGATCGCCTGCGCC
AACTCGCCCGCCGAGCTCCTGGAAGTGAAGCGGGCCTACCACGCCATCTACAAGCGCTCGCTGGAGG
AAGACGTCGCCGCTGGCGCCACGGGAAACCTCCGCAATCTCTTGGTCGCTCTGGTGAGCGCCTACAG
GTACGACGGCGATGAGGTGAACGGCGGGCTAGCGAGGTCGGAGGCGAAGATCATTCAAGCGGACGT
GAAAAACGGCGTCACGGCGGATCACGGGGAGCTCATCAGGGTCCTAGGCACCAGGAGCAGAGCCCA
GCTCGGCGCCACGTTCAACTGCTTCAGAGATGAGCACGGCACCACCGTCACCAAGGCCCTGCTGCAT
GCCCCCGACACGACGGGCTACGCGCGTGCGCTGCGGACCGCGGCGAGGTGCATCGCCGACACAGAC
AAGTACTTCGCCAAGGTCCTGAGGAACGCGATGCACAAGTCGGGGACCGACGAGGAGAGCCTGCTCC
GGGTGGTGGTGATGCGCGCGGAGAAGGACCTCAGGGGCATCAAAGACGAGTTCCACAAGAGGGCGT
CGGTTGCCCTGGAGCAAGCCATCGCCAAGGAAACCTCCGGTGACTTCAAGACCTTCATCACGGCTCTT
GTTGGGACTAGCCAATAA
>AeAnn5 AEGTA42363
ATGCCAACAGGGACAGCTGCAGCCGAGGCCGGACATGGGACGCCTTATGTGGATCCGGAAGATGGGG
AGGAAGGAGAGTGGGGCGCGAGATGGCGGGCGGACAAGGAGGCCCTGGTGCGCATACTGGCACGGA
GGACGGCGGCGCAGCGGTCGGCGATCCGCCGCGCCTACGCCTTCCTCTTCCGGGAGCCCCTCCTCAA
CTCCTTCCGCCAGCGGCTCTCCCGCCAGTACTGCCCCGTCACCGTCGACTTCTGGGTACGGCAGCGCA
CGCATGCATCTCTCCGAAGGCGGTCATCCTGTGGACGATGGACCCGGCGGAGCGGGACGCGAACCTG
GTGCACGGCGCGCTGCGGCGGCGAGGGGACGGCGACCACCTCGCCGTGCTGGTGGAGGTCTCGTGC
GCGGCCGGACCCGACCCCCGCCGGGNGCGCCTACCGCTCCCTCTTCGGCTGCTCCGTGGAGGAGGAC
CTCGCCTCCTGCGCCGCGCTCCAGCAGCCGCTCAGGAAGATGCTGGTGAGCCTCGTGAGCTCCTACCG
CTACGGCGGTGACCGCGTCGACGCGGACGTGGCGAAGCTGGAGGCGTCGCAGCTCTCGGAGGCCGT
CCGGAGGAAGCAGCCGCACCACGACGAGGTGGTGCGGATCCTCAGCACCCGGAGCAAGCCCCAGCT
CAGGGCGACGCTCCGGCGGTACAGGGAGGACCACGGCACCGACATCGTCGAGGACATCGACAGCCG
CTGCAGCAGCCAGTTCGCCAGGACGCTGAAGAGCGCCGTCTGGTGCCTGACCTCGCCGGAGAAACAC
TTCGCGGAGATGATCAGGGAGTCGGTGGTGGGGCTGGGGACTTACGAGGACATGCTGACGAGGGTGG
TGGTGTCGCGGGCGGAGATCGACATGAGGCAGATCAAGGAGGAGTACAGGGCCAGGTTCAAGACCA
CCGTGACCTGCGACGTCGTTGACGACACCTCCTTCGGCTACAAGGACATCCTGCTGGCCCTGGTTGGC
GGCGAGGAAGAGTGA
>AeAnn6 AEGTA16509
ATGGCGAGCCTGAGCGTGCCTCCGGTGCTCACGTCCCCGCGCCACGACGCCATCGCCCTCCACAGGG
CCTTCAAAGGGTTCGGGTGCGACAGCACGACGGTGATCAACATCCTGGCGCACCGCGACGCGAAGGC
GATGCTGCTGTGGGTGCTGGACCCCGTGGGCCGCGACGCCGCCATCCTGAACCAGTCCCTCAACGGC
GACATCACCGACCTGCGCGCCGCCACGGAGGTCATCTGCTCCCGGACGCCGTCGCAGCTGCAGATCAT
GAAGCAGACCTACCGCGCCCGCTTCGGCTGCTACCTCGAGCAGGACATCACCGAGCGCACCTACGGC
GACCACCACAAGCTTCTGCTGGCGTACTTGGGGATCCCGCGGGCAGAGGTGGACATGCAGTACATCA
AGGCCGAGTACCACAAGAAGTACAAGCGCTCGCTGGCCGACGCCATCCACTCCGAGACCTCCGGGAA
CTACCGGACCTTCGTCCTCTCCCTCGTCGGCCGTGACCGCTAA
>AeAnn7 AEGTA11977
ATGCCATGCATGGTAACATGCGTACATGACGAAGGCGGGGGCGCGGACGAGAAGGCGCTGGTGGAGA
TCCTCGGCCGGCGAACCGCCGCGCAGCGCGCCGAGATCCGCCGGGCCTACGCGAGCCTCTACAAGGA
GTCCCTCCTCACCCGCCTCCACGGCGAGCTCTCCGGCCACTTCCAGAAAGCGATGGTGCTGCTGGCGA

CGGACCCGGCGGAGAGGGACGCCAAGCTGGCGAGGGAGGCCCTGGGCAGGCGGGGCGACGACAGG
GACGCGTGGGTGCTCATCGAGGCCGCCTGCGCCGCCGCGCCGGACCACCTCGTCGCCGTCCGCCGCG
CGTACCGGTCCCTCCATGGATCCTCGCTGGAGGAGGACGTCGCCGCCTGCTCCGCGTTCCAAGAACCG
CTCAGGAAGGTGACTACTCCAGGAGAAGAACAGAGCAATCCATTCCAGAGTATACTGCTGGTGAGCC
TGGTGAGGTCGTACCGGTGCGCGGAGGAGAGCGTGGACATGGACGTGGCGAAGCTGGAGGCCGCGC
AGCTGGCGGAGGCCATCCGGAGGAAGAAGCAGCCGCACGCCGGCGAGGTCGTCCGGATCGTGAGCA
CAAGGAGCAAGCCGCAGCTCGCCGCCACGTTCCGGTGCTACAAGGAACAACATGGCAGCGACATCGA
AGAGGACATGAAGCAGTACAGCAGCAGCCAGTTTGCAAGGATGCTCAAGATTGCTGTCTGGTGCCTC
ACATCTCCTGAAAAGCACTTCGCAGAGGTTATCAGGTACTCCATCTTAGGGCTTGGAACGGACGAGGA
CGCACTCACCAGGGCAATCGTGTCCAGGGCCGACATCGACATGAAAAAGATCAAGCAAGAGTACAGG
GTCAGATTCAAGACGACCGTAACAGACGATGTCGTCGGCGATACCTCGGGGTATTACATGGATATCTTG
TTGGCCTTAGTAGGGAAGGAGTGA
>AeAnn9 AEGTA10144
ATGGCGAGCCTCACCTTGCCGCCGGCGCCCCCGAACCCGCGCCAGGACGCCATCGACCTCCACAAGG
CCTTCAAAGGGTTTGGCTGTGATAGTACAACAGTTTCAAATATACTTTCTCACCGTGACTCAATGCAAC
GTGGATACATTCAACAGGAATACAAAACTATGTATTCTGAGGAACTTTCACATCGTATATCGTCTGAACT
CAGTGGAAACCACAAGGTATGGAAAATTGTAGAAAAATCTTTATTGCATGATAAATCAAACTATTGCAC
AGCTATACTAGCTGAAGAGAAAGCACTGTCGCTCTGGATTCTTGATCCTGCTGGACGTGATGCGACTG
TTCTGAAAGAAGCTCTAAGTGCTGAAAGTCTTGATCTGAAAGCAGCTACTGATATAATTTGTTCCAGGA
CACCATCACAGCTGCAAATAATGAAACAGACATATTATGCAAAGTTTGGTACTTATCTTGAGCATGACA
TTAGTCAGCAAGCATCCGGCGATCATCAGAAGATCTTACTAGCCTATGTTGGCATTCCACGCTACGAAG
GCCCGGAGGTTGATCCCACTATAGTGACACATGATGCGAAGGACCTCTACAAAGCTGGTGAGAAAAA
GCTGGGCACAGATGAGAAAACCTTCATCCGCATCTTCACTGAGCGCAGCTGGGCACACATGGCAGCT
GTTGCTTCTGCTTACCGTCACATGTATGATCGGTCGTTACAGAAGGTTGTGAAGAATGAAACATCTGGA
AACTTTGAAGTTGCTCTGTTAACTATCCTCAGATGTGCTGAGAATCCAGCGAAGTATTTTGCTAAGGTG
TTGAGGAAGTCCATGAAAGGTCTAGGTACTGATGACAAGACACTTGTAAGGGTTGTGGTAACAAGGA
CTGAGATTGATATGCAGTATATCAAGGCAGAATATTACAAGAAATACAAAAAGCCGTTGGCTGATGCTA
TCCATTCCGAAACATCAGGCGGTTATCGGACGTTCCTGCTTTCTCTTGTTGGTAGCCATTAG
>AeAnn10 AEGTA28110
ATGGCTGCCCGTTCTCCTGCCGCCGCAGGGTTCGAGAACGAGTGCAGAGAGATCCACGGAGCATGCG
ACGAGCCGCGCCGCCTGAGCCGCCTCCTGGCTCACCGGAGCGCGTCGGAGAGGCAGCAGATCAAGG
CGACGTACCGCGCAATGTTCGGCGAGGACCTCGCCGGCAGGCTCCATAAAACCCTCACGGCCAATCA
GGACAACGAGTCTCAGCATGGCCTCCCTCTCCAGCTCTGCAACCTGCTCTACCTGTGGATGCTTGACC
TGGCGGAGCGAGACGCGATCATGGCGAGGGACGCCATCGAGAGCGGCGCGGCGGCCGATTACCGCGC
CCTTGTCGAGGTCTTCACGCGGCGGAAGCAGGACCAGCTCTTCTTCACCAAGCAGGCGTACCTGGCC
AGGTTCAAGAAGAACCTGGAGCAGGACATGGTCACCGACCCCTCGCACCCTTATCAGAGACTATTGGT
AGCTCTGGCAACCTCCCACAAGTCGCACCACGATGAACCCAGCCGGCACATCGCGAAATGCGACGCC
AGGCGATTGTACGACGCCAAGAACGGCGGCGGCACGGGATCGGTCGACGAGGCCGCCATCCTCGAGA
TGTTCAGCAAGAGGAGCATCCCACAGCTCAGGCTGGCGCTCTGCAGCTACAAGCACATATATGGGCAT
GACTTCACCAAAGCACTGAAGAAGAATTTGGCTGGCGATTTTGAAGAGTCTCTGAGAGTTGTTGTCA
AGTGCATCTACACTCCTTCCAAGTATTACTGTAAGTTACTGCAGAGGAGTATGCAACGCCCAGAAGCT
GATAAAAGGTTGGTTACGAGGGCTATCTTGGGCAGCGACGACGTCGGCATAAACGAGATCAAGTTGGC
GTTCAAGAGTAACTTTGGAAAGAACCTCGCAGATTTCATCCATGAAAGCTTACCACAGAGTGATTACA
GAGAGTTTCTTTGGTTATGGCAAGGGGGCCAGTGCCTAGTAGCATCATAA
>AeAnn11 AEGTA31729

ATGGCGACGCTCTCCGTCCCCGCCGCCGTCCCGGCCGTCGCCGAGGACTGCGAGCAGCTGCGCAAGG
CGTTCGCGGGGTGGGGCACCAACGAGCGCCTCATCGTCTCCATCCTGGCGCACCGCGACGCCGCGCA
GCGCCGCGCCATCCGCCGCGCCTACGCCGAGGCCTACGGCGAGGAGCTGCTCCGCGCCATCGGCGAC
GAGATCCACGGCAAGTTCGAGAGGGCGGTGACCCAGTGGACGCTGGACCCGGCGGAGCGGGACGCG
GAGCTGGCCGGCGAGGAGGCCAAGAAGTGGCAGCCGGGGGGCCGCGCGCTCGTCGAGATCGCCTGC
GCGCGCACCCCCGCGCAGCTCTTCGCCGCCAAGCAGGCCTACCACGACCGCTTCAAGCGCTCGCTCG
AGGAGGACGTCGCCGCGCACGTCACCGGCGACTTCCGCAAGCTTCTGGTGCCTCTTGTAAGCGCATAC
CGCTACGATGGTCCGGAGGTGAACACATCGTTGGCACACTCTGAAGCCAAAATACTCAATGGAAAGAT
CAACGAGAAGGCCTACAGTGATGATGAGATCGTCAGGATTCTCACTACAAGGAGCAAGGCTCAGCTA
CTTGCAACATTCAACAGCTACAATGATCAGTTCAGTCATCCCATCACTAAGGACCTCAAGGAAGACCC
AAAGAACGAGTTCCTCTCAACCCTGAGGGCGATCATAAGGTGTTTCACTTGCCCTGACAGATACTTTG
AGAAGGTCGTCCGGTTGGCCCTTGGAGGCGTCGGCACAGACGAGAACACCCTTACGAGGGTCATAAC
CACTCGCGCCGAGGTGGACCTGAAGGTGATAAAGGAGGCGTACCAGAAGAGGAACAGCGTCCCGCT
GGAGAAGGCTGTTTCCAAGGAAACAAGCAGAGACTATGAAGATATGATGCTCGCACTTCTGGGGGCA
GAGTATTGA
>AeAnn12 AEGTA27331
ATGGCGACGCTCAAGGTCCCCTCCAACGACCCGGCCCTCGCCGACGACTGCGACAACCTCCGCAAGG
CCTTCCAAGGGTGGGGCACGAACGAGGCGCTCATCATCTCCATCCTGGGCCACCGCGACGCCGCGCA
GCGCCGCGCCATCCGCAAGCACTACGCCGACACCTACGGCGAGGAGCTGCTCCGGAGCATCACCGAC
GAGATCTCCGGCGACTTCGAGAGGGCCGTGATCCTGTGGACGCTGGACCCGGCGGAGCGGGACGCGG
TGCTCGCCAACGAGACCGCCAAGAAGTGGCACCCCGGGAACCCCGTGCTGGTCGAGATCGCCTGCGC
GCGCGGCTCCAAGCAGCTCTTCGCTGCCAGGCAGGCCTACCATGACCGCTTCAAGCGCTCGCTCGAG
GAGGACGTCGCTGCACACGTCACCGGCGACTTCCGCAAGTATCTTATCCCTTTGGGCTACATTCAGATC
AAATTCAGTTGCGATGGTGGCTTTTGTTACCTTGTTCCTGGCCATTTTCTGATTTTGGAGCTGTTGGTGC
CACTTGTAAGCTCACACCGCTATGAGGGACCAGAGGTCAACACAAGGTTGGCTCATTCAGAGGCCAA
ATTACTGCATGAGAAGATTGAGCATAAGGCTTATGGTGATGATGAGGTCATCAGAATTCTCACCACTAG
GAGCAAAGCTCAGCTGCTTGCAACATTCAATAATTACAATGATACATTTGGTCACCCAATCACTAAGGA
TCTGAAGGCTGACCCCAAGGATGAGTTCCTCAAGACCCTGCGGGCTGTTATCCGGTGCTTCACCTGCC
CTGATAGGTACTTTGAGAAGGTGGCCAGGCTGGCCATTGCAGGGAATGGAACAGATGAGAACTCCCT
CACTAGGGTCATCACCACCCGTGCCGAGGTGGACCTGAAGCTGATCAAAGAGGCGTACCAGAAGAGG
AACAGTGTTCCCCTGGAGAAGGCCGTCGCAGGCGACACTTCCGGCGACTATGAGAGCATGCTCCTTG
CCCTCCTGGGGAAGGAGTAG
