# Supplementary material for: Comprehensive analyses of the annexin gene family in wheat
Source: BMC Genomics. 2016 May 28;17:415. doi: 10.1186/s12864-016-2750-y (PMC4884362; doi:10.1186/s12864-016-2750-y)
Supplement: Additional file 14: Table S7. — Identifiers for annexin genes on Microarrays in Wheat. (PDF 81 kb) [file 12864_2016_2750_MOESM14_ESM.pdf]

Additional file 14: Table S7. Identifiers for annexin genes on Microarrays in Wheat.

Type	Gene	Identifiers
Wheat 61K Affymetrix microarray	*TaAnn1-A*	*Ta.9484.1.S1_at*
	TaAnn1-B	*Ta.9484.1.S1_at*
	TaAnn1-D	*Ta.9484.1.S1_at*
	TaAnn2-A	*Ta.3378.2.A1_at*
	TaAnn2-B	*TaAffx.123163.1.S1_at*
	TaAnn2-D	*TaAffx.123163.1.S1_at*
	TaAnn3-A	*Ta.25635.1.S1_at*
	TaAnn3-B	*Ta.25635.1.S1_at*
	TaAnn4-A	*Ta.1577.1.S1_at*
	TaAnn4-B	*Ta.1577.1.S1_at*
	TaAnn4-D	*Ta.1577.1.S1_at*
	TaAnn5-B	*Ta.16714.1.S1_s_at*
	TaAnn6-A	*Ta.9484.1.S1_at*
	TaAnn6-B	*Ta.9484.1.S1_at*
	TaAnn7-A	*Ta.4305.2.S1_at*
	TaAnn7-B	*TaAffx.55362.1.S1_at*
	TaAnn7-D	*Ta.4305.1.S1_at*
	TaAnn8-A	*Ta.18318.1.S1_at*
	TaAnn8-B	*Ta.18318.1.S1_at*
	TaAnn8-D	*Ta.18318.1.S1_at*
	TaAnn9-A	*Ta.13966.1.S1_at*
	TaAnn9-B	*Ta.13966.1.S1_at*
	TaAnn9-D	*Ta.13966.1.S1_at*
	TaAnn11-A	*Ta.1577.1.S1_at*
	TaAnn11-D	*Ta.1577.1.S1_at*
	TaAnn12-A	*Ta.14590.2.S1_at*
	TaAnn12-D	*Ta.14590.1.S1_at*
NetAffx™ Analysis Center wheat targets	*TaAnn10-A*	*TaAffx.114559.1.S1_x_at*
	TaAnn10-B	*TaAffx.114559.1.S1_x_at*
	TaAnn10-D	*TaAffx.114559.1.S1_x_at*
