# Supplementary material for: Comprehensive analyses of the annexin gene family in wheat
Source: BMC Genomics. 2016 May 28;17:415. doi: 10.1186/s12864-016-2750-y (PMC4884362; doi:10.1186/s12864-016-2750-y)
Supplement: Additional file 15: Table S8. — Primer pairs used for the expression analysis of wheat annexin genes by quantitative RT-PCR. (PDF 18 kb) [file 12864_2016_2750_MOESM15_ESM.pdf]

Additional file 15: Table S8. Primer pairs used for the expression analysis of wheat annexin genes by quantitative RT-PCR.

Name	Forward primer	Reverse primer
TaAnn1-D	5' GTGCGAGGTTCGGTTGCTAC 3'	5' GCCTTCGTTGCGTGGGAC 3'
TaAnn2-A	5' TGCTTCAGTTCCGATGCTCTTC 3'	5' GTCACCGTCGTCTTGTACCTCA 3'
TaAnn3-B	5' CTCATCGTCGGCAAGTAAACC 3'	5' TCAGCCGTACACGACCTCAG 3'
TaAnn4-A	5' CCAACGCCGCCACGAAG 3'	5' AGGCCTGCTTCACTTCCAGG 3'
TaAnn6-B	5' GCGGAAACATCACGCGATAA 3'	5' CGCAAGGAAGAGCCTGGAC 3'
TaAnn7-B	5' ATGAACCGAGGCAACAGTCTATG 3'	5' CGTAATCTTACCTTCGCACTCTTG 3'
TaAnn8-B	5' GCATCCATAACATCCCTCCCC 3'	5' TCCTTCTTCTCCCTCGCTTCC 3'
TaAnn9-B	5' GCTCTGTCGCATTACTTTACCCTA 3'	5' GCAGAAATAGTTGTCAGCGAAGC 3'
TaAnn10-B	5' ACGCCCAGAAGCCGATAAA 3'	5' CCCTTGCCATAACCAAAGAAAC 3'
TaAnn12-A	5' TCCTGTGGACGCTGGACCC 3'	5' CCTGCCTGACGGCGAAGAG 3'
Actin	5' TACTCCCTCACAACAACCG 3'	5' AGAACCTCCACTGAGAACAA 3'
